# Substandard and Falsified Antibiotics and Medicines against Noncommunicable Diseases in Western Cameroon and Northeastern Democratic Republic of Congo

**DOI:** 10.4269/ajtmh.20-0184

**Published:** 2020-05-11

**Authors:** Simon Schäfermann, Cathrin Hauk, Emmanuel Wemakor, Richard Neci, Georges Mutombo, Edward Ngah Ndze, Tambo Cletus, Fidelis Nyaah, Manyi Pattinora, Dorothee Wistuba, Irina Helmle, Christine Häfele-Abah, Harald Gross, Lutz Heide

**Affiliations:** 1 Pharmaceutical Institute, Eberhard Karls University Tuebingen, Tuebingen, Germany;; 2 Le Dépôt Central Médico-Pharmaceutique de la 8e CEPAC (DCMP), Bukavu, Democratic Republic of Congo;; 3 Cameroon Baptist Convention (CBC), Central Pharmacy, Mutengene, Cameroon;; 4 Presbyterian Church in Cameroon (PCC), Central Pharmacy, Limbe, Cameroon;; 5 Institute of Organic Chemistry, Eberhard Karls University Tuebingen, Tuebingen, Germany;; 6 German Institute for Medical Mission (Difaem), Tuebingen, Germany

## Abstract

Falsified and substandard medicines may undermine the progress toward the Sustainable Development Goals. The present study investigated the quality of 13 essential medicines in Cameroon and the Democratic Republic of Congo (DR Congo). Five hundred six medicine samples were collected from the government and faith-based health facilities, private pharmacies, and informal vendors (total 60 facilities). Collected samples were analyzed according to the U.S. Pharmacopeia (USP) for identity, content, and dissolution of their active pharmaceutical ingredients (APIs) and for uniformity of dosage units. Three samples (0.6%) were identified as falsified. Overall, 8.5% of the samples failed USP specifications for the content of the API and 11.7% failed dissolution testing. Medicines from informal vendors showed a higher out-of-specification rate (28.2%) than other types of drug outlets (12.3%; *P* < 0.0001). All three falsified medicines had been sold by informal vendors. The failure rate of medicines stated to be produced in Europe (5.1%) was lower than that for medicines from Asia (17.7%; *P* = 0.0049) and Africa (22.2%; *P* = 0.0042). Medicines against noncommunicable diseases showed a higher failure rate than antibiotics (25.3% versus 12.1%; *P* = 0.0004). Four hundred fifty-one of the samples were analyzed in Cameroon and the DR Congo with the Global Pharma Health Fund Minilab (thin-layer chromatography and disintegration testing). The three falsified medicines were readily detected in Minilab analysis. However, substandard samples were detected with low sensitivity. A well-enforced ban of medicine sales by informal vendors and increased attention to supplier qualification in the procurement process may reduce the prevalence of substandard and falsified medicines.

## INTRODUCTION

In the past decades, access to medicines in low- and middle-income countries (LMICs) has improved,^1,2^ but the occurrence of substandard and falsified (SF) medicines has been reported frequently and was even described as a “pandemic” by some authors.^3^ Substandard and falsified medicines pose a serious risk to global health, and therefore, access to safe, quality, and affordable medicines has been included in the Sustainable Development Goals of the United Nations as Goal No. 3.8.^4^ Substandard and falsified medicines may cause prolonged illness and treatment failures and can also directly harm patients through toxic effects or adverse reactions.^5,6^ Yet, reliable data about their prevalence are sparse.^7–9^ Following the first international conference on Medicine Quality and Public Health in 2018, researchers from all over the world called for investment, policy change, and action to eliminate SF medical products, and they formulated a research agenda stressing the urgent need for epidemiological evidence on the prevalence of SF medical products in different countries, in different sectors of the health system, and for different categories of medicines.^10^

Although medicine quality problems have been reported to occur worldwide, the burden of SF medicines is heavily concentrated in LMICs.^8^ A review article by the WHO calculated an average prevalence of 10.5% SF medicines in these countries.^7^ A review and meta-analysis by Ozawa et al.^11^ estimated their prevalence in Africa to be 18.7%. Both these reviews emphasized the problem of strong heterogeneity of methods and results across different surveys on SF medicines.^7,11^ The lack of a common terminology further hampered the comparison of data from different studies, until finally the 2017 World Health Assembly agreed on common definitions for “substandard” and “falsified” medicines.^12^ Substandard medicines are now defined as “authorized medical products that fail to meet either their quality standards or specifications or both.” They may result from poor manufacturing, or from inappropriate transport or storage conditions. Falsified medicines are defined as “medical products that deliberately or fraudulently misrepresent their identity, composition, or source.”^12^

In the Democratic Republic of Congo (DR Congo) and in Cameroon, so far only few medicine quality studies have been conducted, mostly focusing on antimalarials, antiretrovirals, and antibiotics. The Quality of Selected Antimalarial Medicines Circulating in Six Countries of Sub-Saharan Africa (QAMSA) study conducted by the WHO in six African countries reported that in Cameroon, 37% of the 41 tested antimalarial samples failed quality testing.^13^ Petersen et al.^14^ investigated 869 medicines from seven African and Asian countries using the Minilab of the Global Pharma Health Fund (GPHF, Giessen, Germany).^15^ For those samples which failed Minilab testing, confirmatory analysis was carried out using high-performance liquid chromatography (HPLC). In Cameroon and in the DR Congo, 7.1% and 2.7% of the samples collected were found to be falsified or substandard, respectively, although the authors noted that a number of substandard medicines may have escaped detection because of the limited sensitivity of the GPHF Minilab.^14^ In 2018, Mufusama et al.^16^ reported the quality of artemether/lumefantrine combination products collected in eight cities of the DR Congo. When analyzed using thin-layer chromatography (TLC) with the GPHF Minilab, four of the 150 investigated samples (2.7%) were found not to contain the declared active pharmaceutical ingredients (APIs), and this was confirmed by HPLC analysis. The failure rate reportedly increased to 46.7% when also quantitative deviations from the declared amount of the APIs were considered. The authors noted that this failure rate was quite high compared with other medicine quality surveys. Schiavetti et al.^17^ investigated the quality of medicines used in children, supplied by private wholesalers in Kinshasa in the DR Congo in 2018. Of the 239 tested samples, representing artemether/lumefantrine and amoxicillin powders for suspension and paracetamol tablets, 27% were of poor quality. By contrast, 35 antiretroviral medicine samples collected in different regions of Cameroon all showed good quality.^18^

As emphasized in the WHO Global Status Report on non-communicable diseases (NCDs) of 2014,^19^ the burden of death and disease resulting from NCDs is heavily concentrated in LMICs. Hunter-Adams et al.^20^ expected that the burden of diabetes in Africa will be more than double in the next decade. Nevertheless, so far, the quality of medicines against NCDs has only been evaluated in few studies. The SEVEN study investigated the quality of seven cardiac medicines from 10 different countries, including the DR Congo,^21^ and 26.7% of the 90 samples collected in the DR Congo were reported to be of poor quality.

Following the aforementioned call for research on the prevalence of SF medicines in different countries, in different sectors of the health system, and for different categories of medicines,^10^ the present study investigated the prevalence of SF medicines among selected medicines against NCDs and antibiotics in government and faith-based health facilities, private pharmacies, and informal vendors of Cameroon and of the DR Congo. Samples were first tested with the GPHF Minilab. Subsequently, all samples, irrespective of the results obtained in the GPHF Minilab analysis, were also tested with the methods of the U.S. Pharmacopeia (USP) for identity, content, and dissolution of the APIs and for uniformity of the dosage units. The use of both Minilab and compendial analysis in the present study allows an evaluation of the sensitivity and specificity of the screening with the GPHF Minilab. Data on the availability, prices, and affordability of the medicines were collected additionally and have been published elsewhere.^22^

To the best of our knowledge, this is the largest and most comprehensive study on medicine quality conducted in Cameroon and the DR Congo so far, and at the same time, the largest investigation of the dissolution of the APIs of medicines on the African market published until now.

## MATERIALS AND METHODS

### Study design and included medicines.

This study was designed observing the recommendations contained in the WHO guidelines on the conduct of surveys of the quality of medicines^23^ and the Medicine Quality Assessment Reporting Guidelines (MEDQUARG guidelines).^24^ Thirteen medicines, that is, seven antibiotics and six medicines against NCDs were included, in dosages for adults. They are listed in Table 1. All of them were selected from the essential medicines lists of the Republic of Cameroon^25^ and the DR Congo.^26^ Medicines were selected for which both a USP-finished pharmaceutical product monograph and a GPHF Minilab method were available for medicine quality analysis. The included medicines were identical in both countries with one exception: in the DR Congo, atenolol tablets were included, but in Cameroon, the local partners and Jingi et al.^27^ reported that atenolol was not frequently used. On request by the local partners, glibenclamide (=glyburide) was included instead of atenolol in Cameroon.

**Table 1:** Limits for compliance/noncompliance, and for moderate and extreme deviations from pharmacopoeial specifications, used in this study.

International nonproprietary names	Dosage form	Content of the API (=assay) (% of declared content)			Dissolution of the API (% of declared content)		
		Complies	Moderate deviation	Extreme deviation	Complies	Moderate deviation	Extreme deviation
Amoxicillin	Tablets	90–120	80 to < 90	< 80 or > 120	≥ 85	< 85 to 60	< 60
Clavulanic acid		90–120	80 to < 90	< 80 or > 120	≥ 80	< 80 to 55	< 55
Amoxicillin	Tablets	90–120	80 to < 90	< 80 or > 120	≥ 75	< 75 to 50	< 50
Amoxicillin	Capsules	90–120	80 to < 90	< 80 or > 120	≥ 80	< 80 to 55	< 55
Ciprofloxacin	Tablets	90–110	80 to < 90 or > 110 to 120	< 80 or > 120	≥ 80	< 80 to 55	< 55
Doxycycline	Tablets/capsules	90–120	80 to < 90	< 80 or > 120	≥ 85	< 85 to 60	< 60
Doxycycline hyclate	Tablets	90–120	80 to < 90	< 80 or > 120	≥ 85	< 85 to 60	< 60
Doxycycline hyclate	Capsules	90–120	80 to < 90	< 80 or > 120	≥ 80	< 80 to 55	< 55
Penicillin V	Tablets	90–120	80 to < 90	< 80 or > 120	≥ 75	< 75 to 50	< 50
Metronidazole	Tablets	90–110	80 to < 90 or > 110 to 120	< 80 or > 120	≥ 85	< 85 to 60	< 60
Sulfamethoxazole	Tablets	93–107	80 to < 93 or > 107 to 120	< 80 or > 120	≥ 70	< 70 to 45	< 45
Trimethoprim		93–107	80 to < 93 or > 107 to 120	< 80 or > 120	≥ 70	< 70 to 45	< 45
Atenolol	Tablets	90–110	80 to < 90 or >110 to 120	< 80 or > 120	≥ 80	< 80 to 55	< 55
Furosemide	Tablets	90–110	80 to < 90 or > 110 to 120	< 80 or > 120	≥ 80	< 80 to 55	< 55
Glibenclamide (glyburide)	Tablets	90–110	80 to < 90 or > 110 to 120	< 80 or > 120	≥ 70	< 70 to 45	< 45
Hydrochlorothiazide	Tablets	90–110	80 to < 90 or > 110 to 120	< 80 or > 120	≥ 60	< 60 to 35	< 35
Metformin	Tablets	95–105	80 to < 95 or > 105 to 120	< 80 or > 120	≥ 70	< 70 to 45	< 45
Salbutamol (albuterol)	Tablets	90–110	80 to < 90 or > 110 to 120	< 80 or > 120	≥ 80	< 80 to 55	< 55

API = active pharmaceutical ingredients. United States Pharmacopeia 41 specifications were used for compliance/non-compliance. Following the suggestion of the QAMSA study by WHO,^13^ extreme deviation was defined as an API content deviating by more than 20% from the declared amount, and/or an average dissolution of the API of the tested units falling more than 25% below the pharmacopoeial Q-value. In this study, all observed assay failures were due to insufficient API content, no sample failed due to excessive API content (see Results section).

### Ethical approval.

This study was approved by the Ministry of Health of the DR Congo (Ref. CAB/Min-Prov/SGFEAHRAP/SK/01/2017) and by the Ministry of Public Health of the Republic of Cameroon, Comité National d’ Ethique de la Recherche pour la Santé Humain (Ref. 243674339).

### Sampling sites.

**Figure 1.**
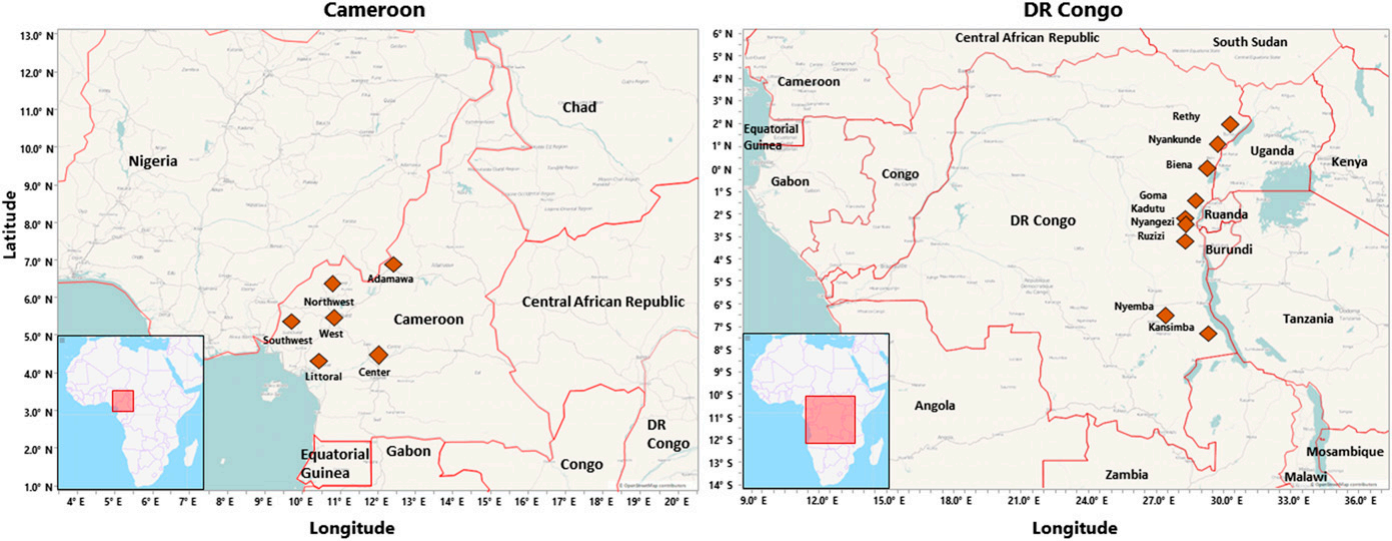
Map of the locations from which samples were collected in Cameroon and the Democratic Republic of Congo (DR Congo). This figure appears in color at www.ajtmh.org.

This study was conducted in the northeast of the DR Congo in the provinces Ituri, North Kivu, South Kivu, and Tanganyika, and in western Cameroon, in the regions Adamawa, Centre, Littoral, Northwest, Southwest, and West (Figure 1) because these were the provinces/regions where the local partners worked. The selection of the sampling sites has been described in the evaluation of the availability and prices of the included medicines.^22^ For the four provinces in the northeast DR Congo, a complete list of the health zones (total 116 zones) was obtained. On consultation with the local partners, 70 of these zones were identified as unsafe for travel by the study personnel and, therefore, had to be excluded from the study. Of the remaining 46 health zones, two from each of the four provinces were randomly selected using the return random number (RAND) function of Microsoft Excel. In addition, Kadutu Health Zone in Bukavu, South Kivu, was added on request by the local partners because it comprised the biggest unlicensed market for medicines and was considered important in the assessment of medicine quality problems in that region. In the DR Congo, the health zone is a set of health centers linked to a hospital.^28^ In each of the selected health zones, the samples were collected first from the main hospital of that zone. When this was a government-operated general referral hospital, medicines were sampled also from the nearest church health center, private pharmacy, and informal vendor of medicines. Correspondingly, if the main hospital was a church-operated centre hospitalier, medicines were sampled also from the nearest governmental health center, private pharmacy, and informal vendor. In Ituri Province, no informal medicine vendors could be found because tight control was enforced by the authorities in that province following a major medicine scandal.^5^ Therefore, in the DR Congo, samples for this study were collected from 34 medicine outlets, located in nine health zones in four provinces.

The structure of the health system of Cameroon has been described in two recent documents.^29,30^ For the present study, a complete list of the 45 church health facilities in the six included regions was obtained. For each region, one church health facility was randomly selected. Samples were collected from this church health facility and from the geographically nearest governmental health facility, private pharmacy, and informal vendor in that region. By chance, the random selection had not included any church health facility operated by the catholic church, and the local partners requested that of the 10 catholic health facilities existing in the six regions, two were randomly selected and included as well. Therefore, in Cameroon, samples were collected from 26 medicine outlets, located in six of Cameroon’s 10 regions.

### Sample collection.

Samples were collected between August 2017 and November 2018. An overt sampling approach was used in public and church health facilities, that is, the investigators identified themselves and explained the purpose of the study. By contrast, a mystery shopper approach was used in informal vendors and private pharmacies, that is, the local investigators acted as customers, stating that they own a small informal medicine outlet. If the medicine outlets had more than one brand of the included medicines in stock, the cheapest brand was collected. For each sample, an amount of 100 dosage units (capsules or tablets) was purchased if available, otherwise less, but samples were only collected if at least 30 dosage units could be obtained. In government and church health facilities, replacements for the sampled medicines were offered by the sample collectors to avoid that stock-outs would result from this study. Replacement medicines were obtained from the medical stores of the local partner organizations. If the visited facilities preferred, the sampled medicines were paid for.

Samples were purchased in their original containers if possible. Preprinted labels with a unique sample number were attached to each sample on collection. Brand name, batch number, manufacturing date, expiry date, name of manufacturer, international nonproprietary names of the APIs, strength, dosage form, package size, and price were recorded as stated on the labels. All samples were transported from the collection sites to the medical stores of the local partner organizations as fast as possible. Shipment to Tuebingen University, Germany, was done by commercial courier services. At Tuebingen University, the samples were stored in an air-conditioned storage room at 21°C until analysis.

### Chemical analysis.

Of all samples consisting of more than 50 units (=tablets or capsules), 25 units were retained by the local partners for GPHF Minilab analysis in the respective country and the remaining units were shipped to Tuebingen University, Germany, for compendial analysis. For three samples, less than 50 units had been collected, and in these cases, all units were sent to Germany.

Global Pharma Health Fund Minilab analysis comprised visual inspection, TLC, and disintegration testing according to the Minilab manual^15^ and was carried out by the local partners in Cameroon and the DR Congo. Results of TLC analysis were recorded by photographs of the developed TLC plates.

Compendial analysis was carried out at the Pharmaceutical Institute of Tuebingen University according to the monographs of the USP 2018 (USP 41) for the respective finished pharmaceutical products. It comprised identification of the declared API by HPLC in comparison with certified reference standards, and quantification of the API (=assay), dissolution testing, and testing for uniformity of dosage units. Certified pharmaceutical secondary reference standards were purchased from Sigma-Aldrich (St. Louis, MO). Using the columns and solvent systems specified by USP 41, HPLC-UV analysis was carried out using an Agilent 1100 HPLC or an Agilent 1260 Infinity II HPLC (Agilent Technologies, Santa Clara, CA). Dissolution tests were performed with a PTWS 610 Dissolution Testing Instrument (Pharma Test Apparatebau AG, Hainburg, Germany) and an Agilent 708-DS Dissolution Apparatus (Agilent Technologies). Uniformity of dosage units was determined using the test for weight variation which, according to USP 41, is applicable if one unit contains at least 25 mg of the API, and the API comprises 25% or more of the whole tablet or the capsule content weight. In this study, this was applicable for the samples containing amoxicillin, ciprofloxacin, doxycycline, penicillin V, metronidazole, sulfamethoxazole, atenolol, furosemide, and metformin, and thereby for 425 of the 506 investigated samples.

Samples that showed unknown substances in LC-UV analysis were further analyzed using LC-HR-MS/MS and, in case of sample QMC266, nuclear magnetic resonance (NMR) analysis was performed for identification of these unknown substances. LC-HR-MS/MS analysis was conducted in the Institute of Organic Chemistry, Tuebingen University, on a Thermo Scientific UltiMate 3000 HPLC System coupled with an ESI-TOF Bruker maXis 4G (Bruker Daltonics, Billerica, MA) in the positive mode and using high resolution. For NMR analysis of sample QMC266, the tablets were ground and the API was dissolved in methanol. The resulting solution was filtered and evaporated to dryness, and the residue was redissolved in *d*_4_-MeOH. One-dimensional and 2D NMR spectra were recorded at the Pharmaceutical Institute, Tuebingen University, with a Bruker Avance III HD 400 MHz NMR spectrometer (Bruker BioSpin GmbH, Rheinstetten, Germany). NMR spectra were calibrated to the residual solvent signals (*d*_4_-MeOH resonances at δ_H_ = 3.31 and δ_C_ = 49.0 ppm) or the internal offset for^15^ N assigned by the instrument manufacturer.

### Definitions of medicine quality.

For the compendial tests, the limits for compliance described in the respective USP 41 monograph were used. As proposed in the QAMSA study by the WHO^13^ and also applied in our previous study in southern Togo,^31^ samples deviating from USP 41 specifications for assay and/or dissolution were further divided into those showing only moderate deviations from the pharmacopoeial limits and those showing extreme deviations. Extreme deviation was defined as an API content deviating by more than 20% from the declared amount and/or an average dissolution of the API of the tested units falling more than 25% below the pharmacopoeial limit (i.e., below the pharmacopoeial Q value minus 25%).^13^ Table 1 shows the limits for compliance given by USP 41 for all investigated types of medicines and the limits for extreme deviations.

For the definition of falsified medicines, the current WHO definitions were used.^12^ Results of GPHF Minilab TLC and disintegration testing were classified as pass/fail following the instructions of the GPHF Minilab manual.^15^

### Statistical calculations.

Statistical evaluations were performed using JMP 14.2 (SAS GmbH, Heidelberg, Germany). The prevalence of SF medicines and the corresponding CIs were determined by distribution analysis. Significance of differences in the prevalence of SF medicines between different groups was calculated using Fisher’s exact test or Pearson’s chi-squared test. Comparisons of Minilab testing results to compendial testing results were calculated with contingency analysis.

### Information of national authorities and stakeholders.

The Laboratoire National de Contrôle de Qualitè de Médicaments de d’Expertise (LANACOME), Cameroon, and the WHO Rapid Alert System were informed immediately about falsified medicines detected in this study. The complete survey results were shared with the national authorities, that is, the Directeur Général de la Santé, Ministère de la Santé Publique, DR Congo; the Direction de la Pharmacie et du Médicament de la Republique du Congo; the Direction de la Pharmacie du Médicament et des Laboratoires, Ministère de la Santé Publique, Cameroon; and the LANACOME, Cameroon; and with the WHO Rapid Alert System. In addition, the findings of this study were presented to representatives of the African national medicine quality control laboratories at the third African Medicines Quality Forum in Abuja, Nigeria, in February 2020.

## RESULTS

### Overview of collected medicine samples.

A total of 502 medicine samples were purchased from 26 sampling sites in Cameroon and 34 sampling sites in the DR Congo. Visual inspection showed that four samples included packages with two different batch numbers instead of representing a uniform sample. These different batches were subsequently treated as separate samples and analyzed for their quality individually. Therefore, the total sample size was 506.

**Figure 2.**
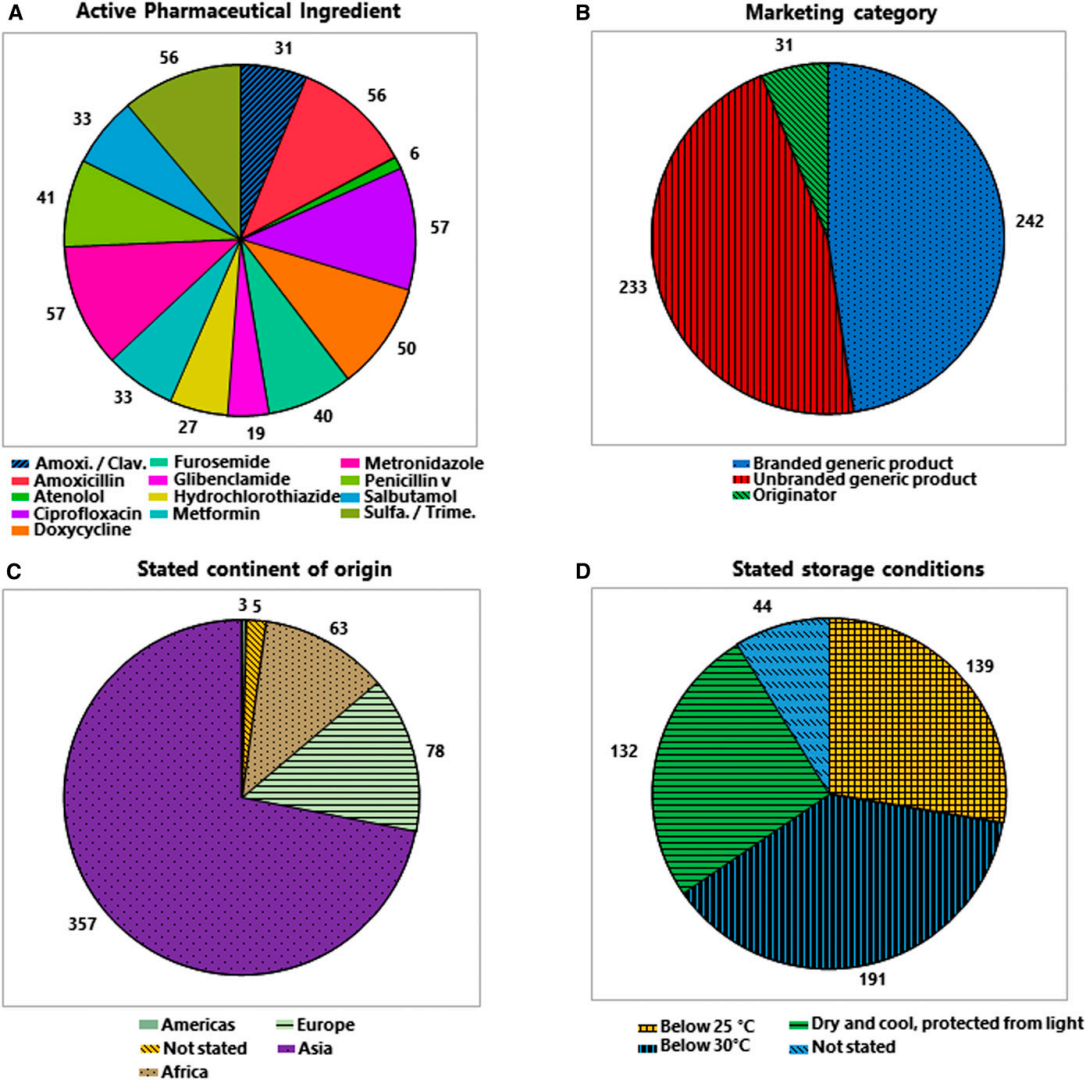
Distribution of all collected samples (*n* = 506) over different categories. In the pie chart in (**A**), the different active pharmaceutical ingredients (APIs) are arranged in clockwise orientation. This figure appears in color at www.ajtmh.org.

The total number of samples collected per type of medicine is depicted in Figure 2A. Obviously, not all medicines were available at all of the 60 sampling sites; therefore, the theoretical number of 60 samples was not reached for any of the included medicines, although ciprofloxacin and metronidazole tablets came close with 57 samples each. A detailed analysis of the availability as well as of prices and affordability of the included medicines has been published in a separate article.^22^

As shown in Figure 2B, originator medicines represented only 6% of the collected samples. The vast majority were generic medicines, either sold under their international nonproprietary name (“unbranded generic products”) or under a brand name decided by the marketing authorization holder (“branded generic products”).

Figure 2C shows the dominance of Asian countries as medicine suppliers to Cameroon and the DR Congo. According to the information stated on the packaging, 357 (71%) of the samples collected were manufactured in Asia, of these 231 in India, 121 in China, and five in other Asian countries. Seventy-eight samples (15%) were stated to be manufactured in Europe and 63 samples (12%) in Africa. With only three samples, the Americas played no significant role in the supply of the investigated medicines.

According to the information stated on the packaging, the collected samples represented 260 different brands (414 different batches), produced by 119 different manufacturers in 26 different countries. A complete list of these manufacturers and countries is given in Supplemental Table S1. The most frequently encountered manufacturer was Medopharm, Chennai, India, representing 42 samples. However, most manufacturers were only represented with very small number of samples (mean = four samples and median = three samples).

**Figure 3.**
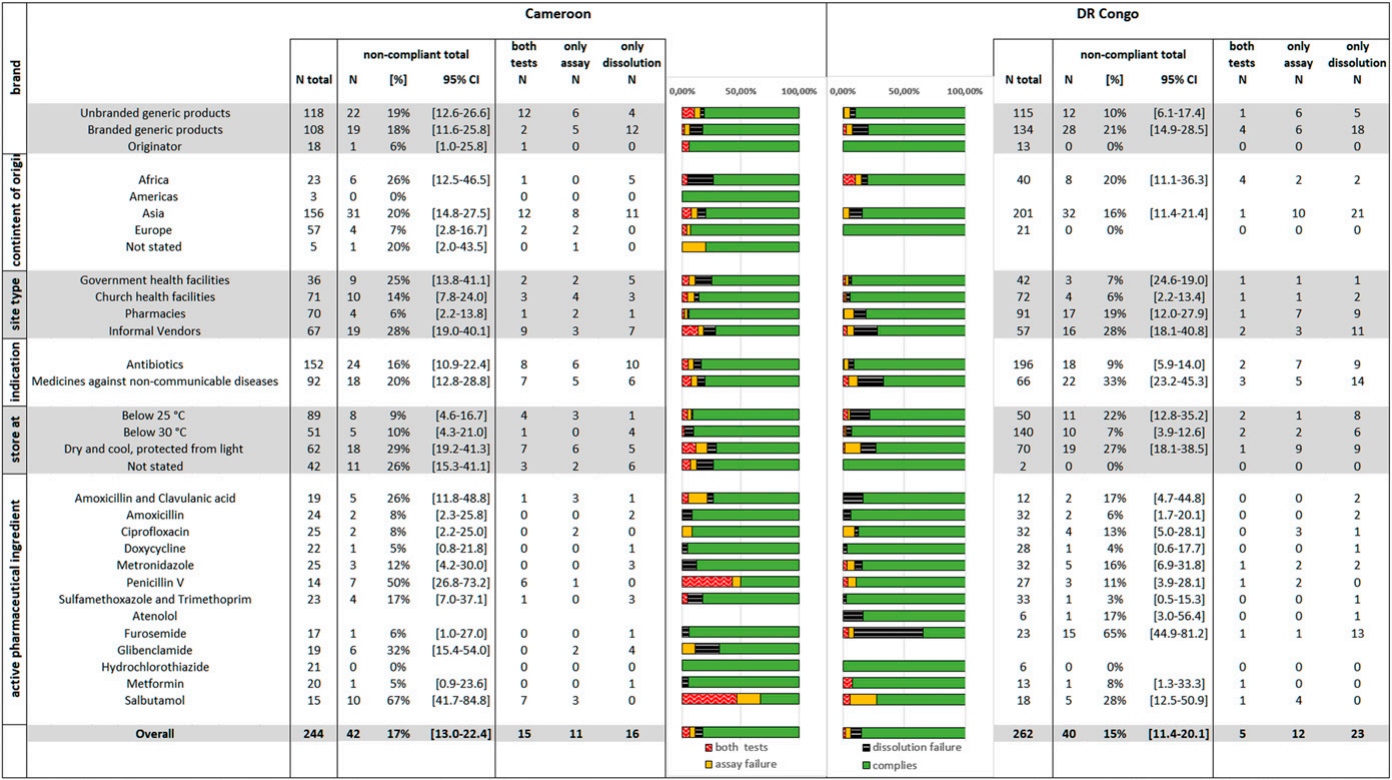
Frequency of noncompliance with pharmacopoeial specifications for assay and dissolution in different subgroups of medicines. This figure appears in color at www.ajtmh.org.

According to current stability testing guidelines for pharmaceuticals,^32–34^ the DR Congo is regarded as climatic zone IVa (hot and humid) and Cameroon as climatic zone IVb (hot and very humid). Medicines intended to be marketed in these two countries should be tested for long-term stability at 30°C/65% relative humidity (DR Congo) or at 30°C/75% relative humidity (Cameroon), respectively. Medicines for which stability has been demonstrated under either of these two conditions should carry the WHO-recommended labeling statement “Do not store above 30°C.”^32–35^ As shown in Figure 2D, however, only 38% of the collected samples indeed showed this statement. Twenty-eight percent of the samples were labeled “Do not store above 25°C,” indicating that they may not have been tested for stability under the appropriate conditions for medicines to be marketed in the DR Congo or in Cameroon. Twenty-six percent of the samples carried less precise, with not WHO-recommended labeling statements such as “Store in a cool and dry place, protected from light,” and 9% had no storage recommendation at all printed on the packaging or leaflet. However, there was a marked difference between the medicines from the two countries (Figure 3). In the DR Congo, 53% of the medicine samples showed the correct labeling statement “Do not store above 30°C,” and only 1% carried no storage recommendation at all. By contrast, in Cameroon, only 21% of the medicine samples showed the correct labeling statement “Do not store above 30°C,” and 17% carried no storage recommendation at all.

Figure 3 furthermore shows the distribution of the samples collected across different marketing categories, stated continents of origin, and types of sampling sites, separately for Cameroon and the DR Congo.

In total, 10 of the 506 samples (2%) were already expired at the time of collection. Although these 10 samples were already expired, they were sold at the point of care to be used in patient treatment. Therefore, also these samples were analyzed for their quality, and the results were included into the overall data analysis. Of these 10 expired samples, two (both representing the same product and batch) were found to deviate from USP specifications in the analysis described in the following paragraphs. They are marked in Supplemental Tables S1 and S3.

Visual inspection showed only a single sample which appeared to be falsified based on its incorrect labeling (penicillin V tablets, described in the next paragraph).

### Falsified medicines.

**Figure 4.**
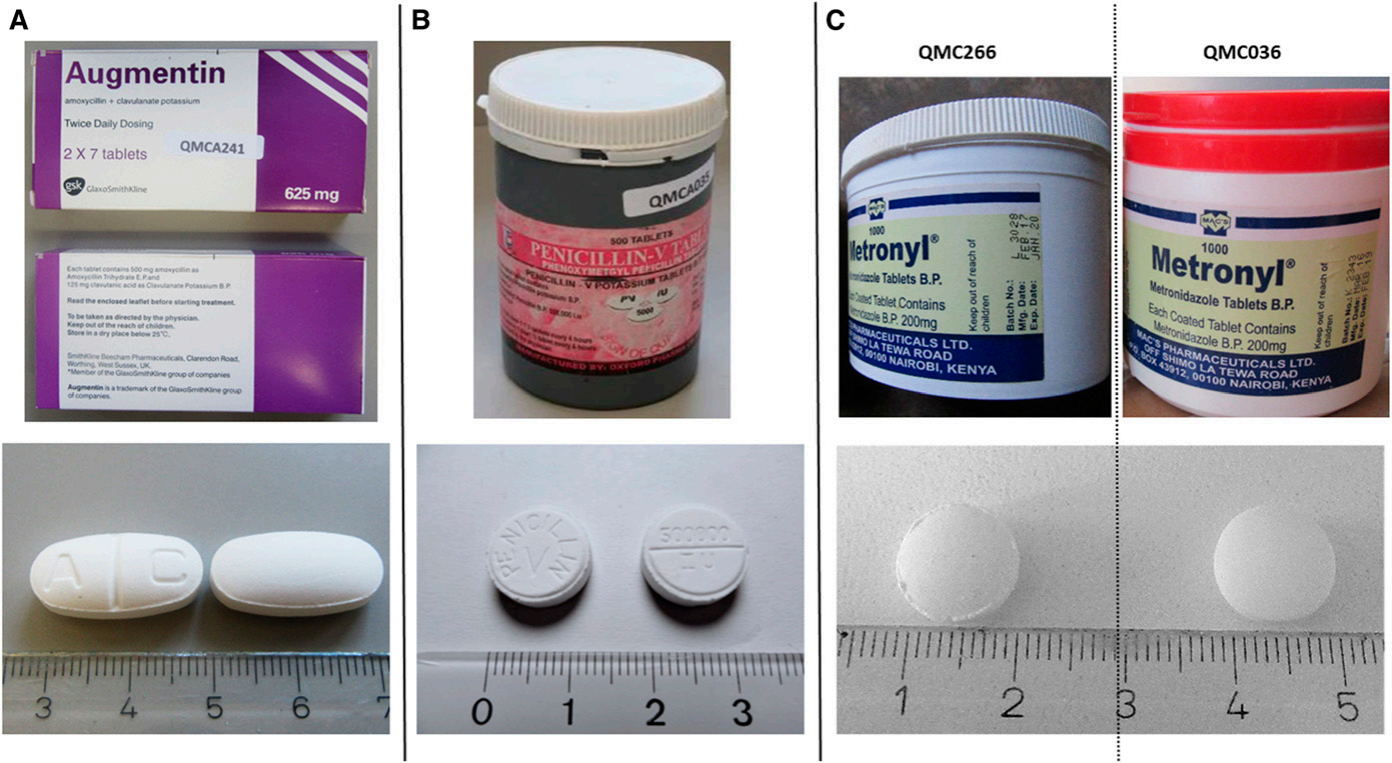
Pictures of the three samples identified as falsified medicines. (**A**) Falsified Augmentin (sample no. QMCA241), containing no detectable active pharmaceutical ingredient (API). (**B**) Falsified penicillin V tablets (sample no. QMCA035), containing 50 mg paracetamol. Note that the API is misspelled on the label. (**C**) Left: falsified Metronyl (sample no. QMC266); manufactured date: February 2017, batch no: L3028, containing 93 mg metronidazole benzoate. Right: Metronyl (sample no. QMC036); manufactured date: March 2016, batch no: K2343, complying with U.S. Pharmacopeia 41 specifications for metronidazole tablets. This figure appears in color at www.ajtmh.org.

Among the 506 medicine samples, three (0.6%) were found not to contain their declared API, and two of these even contained a different, non-declared API. These three samples are shown in Figure 4. Notably, all three of them were sold by informal vendors.

One sample (sample no. QMCA241, Figure 4A), collected in Cameroon, was labeled as “Augmentin^®^ SmithKline Beecham (amoxicillin 500 mg/clavulanic acid 125 mg tablets)” and carried a registration number used for Augmentin by the Nigerian National Agency for Food and Drug Administration and Control. Packaging and tablets appeared to be of excellent quality and gave no immediate indication of falsification. However, both Minilab TLC analysis and HPLC analysis according to USP readily showed complete absence of both stated APIs. The WHO Rapid Alert System was informed and thereupon published a Medical Product Alert about this falsification.^36^ On request by the WHO, the authors of the present article forwarded this sample to the stated manufacturer, who confirmed that this was a falsified medicine not produced by their company.

Another sample (sample no. QMCA035, Figure 4B), also collected in Cameroon, was labeled as “Penicillin-V Tablets, Oxford Pharma Co. Ltd., Belgium.” On the label, the active ingredient was incorrectly spelled as “phenoxymetgyl” rather than phenoxymethyl penicillin (Figure 4). The stated manufacturer “Oxford Pharma, Belgium” does not exist. Although the tablets appeared to have been professionally pressed and embossed, the labels and packaging were of poor quality. Both Minilab TLC analysis and HPLC analysis readily showed complete absence of the stated API but indicated the presence of another, unknown compound, and LC-HR-MS/MS analysis proved that the unknown compound was paracetamol (Supplemental Figure S1). The paracetamol content was found to be only 50 mg per tablet, clearly lower than the content of paracetamol tablets listed in the current WHO Essential Medicines List (100–500 mg).^37^ Again, the WHO Rapid Alert System was informed and published a Medical Product Alert about this falsification.^38^

A third sample (sample no. QMC266; Figure 4C) was labeled as “Metronyl^®^ Metronidazole Tablets B.P., Mac’s Pharmaceuticals Ltd., Nairobi, Kenya.” It was sold in an already opened plastic container by an informal vendor in the DR Congo. Visual inspection gave no obvious indication of falsification. However, both Minilab TLC analysis and HPLC analysis readily showed complete absence of the stated API and the presence of another, unknown compound, and LC-HR-MS/MS (Supplemental Figure S2) suggested that this compound might represent metronidazole benzoate. Subsequently, 1D and 2D NMR spectra were recorded, and a de novo structure elucidation was carried out (Supplemental Figures S3–S11). This confirmed unambiguously that the unknown compound indeed was the benzoic acid ester of metronidazole. ^1^H and ^13^C NMR spectra of the unknown compound and of a metronidazole benzoate standard were perfectly superimposable (Supplemental Figures S9 and S10). Metronidazole has a bitter taste, and the benzoic acid ester of metronidazole is sometimes used as a prodrug with more acceptable taste, both in pediatric formulations and in veterinary medicine.^39^ The metronidazole benzoate content of sample QMC266 was determined as 93 mg per tablet, in clear contrast to the labeling claim of 200 mg free metronidazole. Another batch of the same Metronyl brand had been collected in a government health facility of the DR Congo. That sample (QMC036; Figure 4C) showed an exactly identical label as QMC266, except for the different batch number and expiry date, and was found to be fully compliant with USP specifications in identity, assay, dissolution, and uniformity of dosage units. As shown in Figure 4C, the tablets of falsified sample QMC266 had the same diameter and shape (and also the same weight) as the good-quality sample of Metronyl tablets but showed ridges at the edges, indicating poor manufacturing. Possibly, the plastic container in which sample QMC266 was sold may have originally contained authentic, good-quality Metronyl tablets and may have later been filled with the falsified medicine by the informal vendor. However, this cannot be ascertained from the available information. Attempts of the local partners to find further Metronyl packages remained unsuccessful. Both the stated manufacturer and the WHO Rapid Alert System were informed about this falsified medicine. So far, no answer was received from the stated manufacturer.

All remaining 503 samples were found to contain the declared APIs. Several samples of salbutamol and glibenclamide tablets were found to contain an additional substance which was identified by LC-HR-MS/MS as the preservative methyl 4-hydroxybenzoate (methylparaben). This preservative is considered safe and acceptable, although in most countries, the presence of such a preservative must be stated in the package leaflet.

### Analysis of the quantity of the APIs.

**Figure 5.**
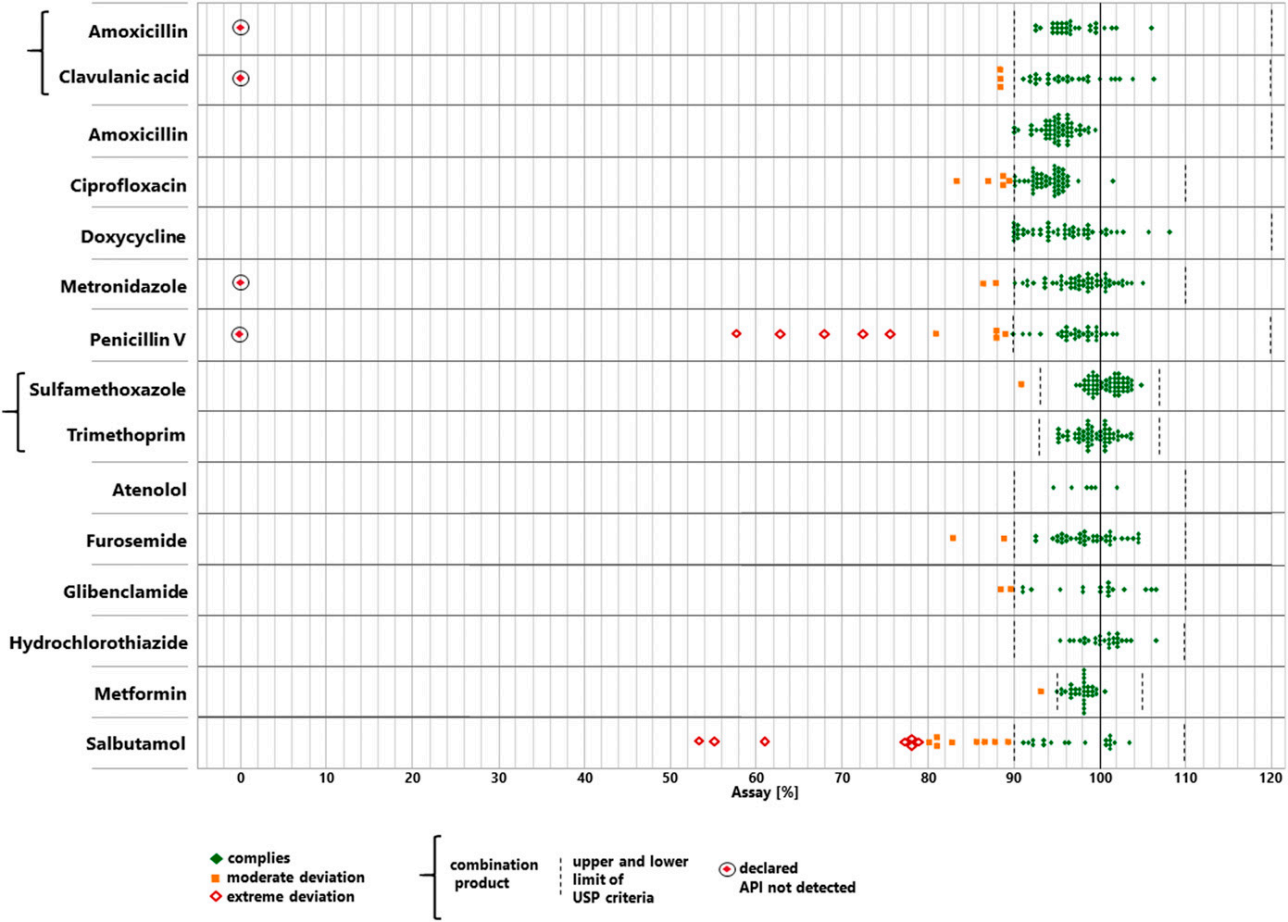
Content of the active pharmaceutical ingredient (API) determined for each sample. This figure appears in color at www.ajtmh.org.

All collected samples were analyzed for the amount of the API (“assay”). Figure 5 shows the API content determined in each of the 506 samples. Different limits for compliance are specified by USP for different APIs (Table 1), for example, 95–105% of the declared content for metformin tablets or 90–120% of the declared content for penicillin V tablets (Figure 5). Four hundred sixty-three samples (91.5%) complied with the USP specifications for assay and are depicted in Figure 5 as green symbols. Twenty-eight samples (5.5%) showed moderate deviations from the pharmacopoeial limits (i.e., deviations not exceeding 20% of the stated content) and are depicted as yellow symbols. Fifteen samples (3.0%) showed extreme deviations (i.e., deviations of more than 20% of the stated content) and are depicted as red symbols; these include the three falsified medicines described earlier (marked with black circles in Figure 5).

The highest proportions of substandard samples in the assay were observed for salbutamol tablets (24% moderate and 21% extreme deviations) and for penicillin V tablets (10% moderate and 15% extreme deviations). None of the samples with other APIs showed extreme deviations in the assay (except the two falsified products of Metronyl and Augmentin described earlier).

In total, 43 samples (8.5%) were noncompliant in the assay. Figure 3 shows the numbers of noncompliant samples separately for Cameroon and the DR Congo. Supplemental Figure S12 shows the API content determined in each of the 506 samples, analyzed by similar subgroups as used in Figure 3.

### Analysis of the dissolution of the APIs.

**Figure 6.**
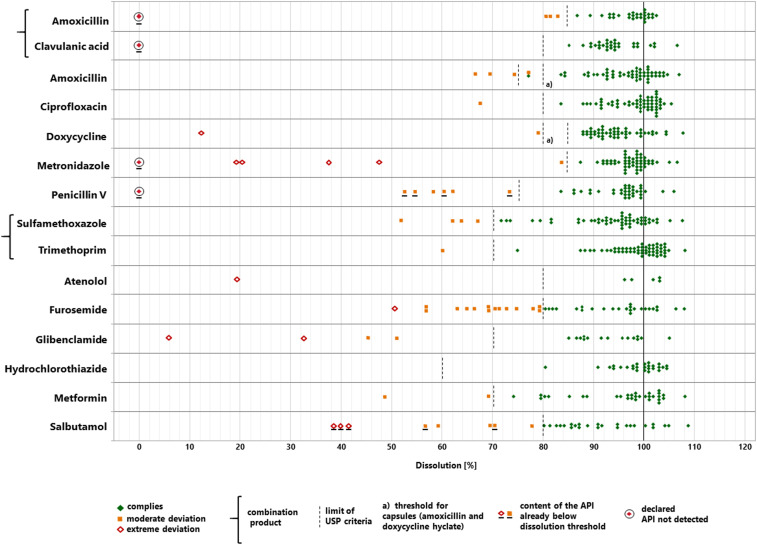
Dissolution of the active pharmaceutical ingredient (API) determined for each sample. This figure appears in color at www.ajtmh.org.

All collected samples were analyzed for the dissolution of the API according to USP 41. Figure 6 shows the dissolution results determined for each of the 506 samples. Again, USP specifies different limits for compliance (“Q values”) for different APIs. For example, USP demands for metronidazole tablets that not less than 85% of the declared API content must dissolve under the specified conditions and for hydrochlorothiazide tablets not less than 60%. In total, 447 samples (88.3%) complied with the USP specifications for dissolution and are depicted in Figure 6 as green symbols. Forty-four samples (8.7%) showed moderate deviations from the pharmacopoeial limits (i.e., an amount of dissolved API lower than, but not more than 25% lower than the pharmacopoeial limit) and are depicted as yellow symbols. Fifteen samples (3.0%) showed extreme deviations (i.e., an amount of dissolved API more than 25% lower than the pharmacopoeial limit) and are depicted as red symbols; these included the three falsified medicines described earlier. In total, 59 samples (11.7%) resulted as noncompliant in dissolution by USP 41 criteria. However, it has to be considered that 12 of these samples (including the three falsified medicines) had already been shown in assay testing to contain an API amount which was lower than the pharmacopoeial limit for dissolution. These samples are marked in Figure 6.

Dissolution failures were observed most frequently for furosemide tablets (*n* = 15), salbutamol tablets (*n* = 8), and glibenclamide tablets (*n* = 4). However, extreme deviations in dissolution were also found for doxycycline, metronidazole, atenolol, and metformin, and of course for the three falsified products described earlier. Figure 6, therefore, illustrates that noncompliance with dissolution specifications is a frequent and serious problem in many of the investigated types of medicines, even more so than noncompliance with assay specifications shown in Figure 5.

Figure 3 shows the numbers of noncompliant samples in different categories of medicines, separately for Cameroon and the DR Congo. Supplemental Figure S13 shows the dissolution results determined for each of the 506 samples, analyzed by similar subgroups as used in Figure 3.

### Uniformity of dosage units.

As explained in the Methods section, uniformity of dosage units was investigated using the test for weight variation which, according to USP 41, was applicable for 425 of the 506 samples. Of the 425 tested samples, 26 (6.1%) failed the test for uniformity (including the three falsified samples). Sixteen (3.8%) of these simultaneously failed in assay and/or dissolution, whereas 10 (2.4%) failed in uniformity testing alone.

### Combined results of compendial analyses.

From the analyzed samples, 8.5% failed in assay testing, 11.7% in dissolution testing, and 6.1% in testing for uniformity of dosage units. Obviously, a number of samples failed in more than one of the mentioned criteria. Therefore, the observed out-of-specification rate calculated from assay testing alone (i.e., 8.5%) increased to 16.2% (i.e., nearly doubled) when also dissolution was considered and to 18.6% when the uniformity of the dosage unit was considered as well.

As correctly stated in an authoritative review by the WHO,^7^ if the goal is to assess the health effects of a medicine, API content and dissolution (which affects bioavailability) are the most important quality criteria. Therefore, hereafter, we focus on assay and dissolution results.

The only API for which no sample was found to be out of specification was hydrochlorothiazide (Figure 3). Notably, of the 27 samples investigated for this API, 14 represented the originator medicine and were sold for very high prices.^22^

Especially high failure rates were observed for penicillin V, furosemide, and salbutamol (Figure 3). For penicillin V tablets, the failure rate was 50% in Cameroon. This was especially due to the four penicillin V samples stated to be produced by a certain manufacturer in China (Shandong Shenglu Pharmaceutical Co. Ltd., Sishui, China, see Supplemental Tables S1 and S3). All four of these samples showed extreme deviations in the assay. No samples from this manufacturer were found in the DR Congo.

Furosemide tablets showed a failure rate of 65% in the DR Congo. This was mainly caused by samples stated to be manufactured by Arco Pharma Pvt. Ltd., Vasai, India, and Prashi Pharma Pvt. Ltd., Mumbai, India (see Supplemental Tables S1 and S3). Eleven of the 12 furosemide samples stated to be manufactured by these two companies from India failed dissolution testing. No samples of these two manufacturers were found in Cameroon.

Salbutamol tablets showed a 66% failure rate in Cameroon largely because of the five salbutamol samples stated to be produced by a certain company in India (Medico Remedies Pvt. Ltd., Maharashtra, India, see Supplemental Tables S1 and S3), four of them even failing with extreme deviations. No medicines of this manufacturer were found in the DR Congo.

A complete list of the manufacturers stated on the labels of the investigated medicines and a summary of the analytical results obtained for the individual (stated) manufacturers are given in Supplemental Table S1. Furthermore, a complete list of all batches and brands investigated, with their stated manufacturers and the analytical results, is given in Supplemental Table S3.

### Analysis using the GPHF Minilab.

Of the 506 collected samples, 451 were analyzed by the local researchers in Cameroon and the DR Congo using the thin-layer chromatographic test and the disintegration test of the GPHF Minilab.^15^ No Minilab analysis was performed for the 49 samples from the Ituri Province in the northeast of the DR Congo because the local researcher left for another position during the time of this study, and no trained replacement could be found in time. For three samples, the small number of tablets collected allowed only for compendial analysis but not for an additional Minilab analysis. For three further samples, the required reagents for Minilab analysis had become unavailable at the local laboratory.

Notably, all three falsified medicines shown in Figure 4 were correctly reported as failing Minilab TLC analysis. These three samples were immediately reported by the local researchers and sent to Tuebingen University for confirmatory analysis, allowing a timely publication of the WHO Medical Product Alerts mentioned earlier.^36,38^

Twelve further samples were reported to fail TLC analysis. Three of these were reported to show insufficient intensity of the TLC spots, indicating an insufficient amount of the API. Two were reported to show additional spots in TLC, and for seven samples, it was not stated in which aspects the TLC test had failed.

Fifteen samples were reported to fail disintegration testing, that is, they did not disintegrate within 30 minutes in water of 37°C, following the procedure described in the GPHF Minilab manual.^15^

In total, 30 of 451 samples (6.7%) were reported to fail Minilab analysis, 15 in the TLC test and 15 in disintegration testing. No sample was reported to fail in both tests. Supplemental Figure S14 summarizes the results of the Minilab tests in the same way as Figure 3 summarizes the results of the compendial analysis.

### Comparison of the results of GPHF Minilab and compendial analysis.

Tables 2–4 compare the results of GPHF Minilab testing with those of compendial analysis according to USP 41. Minilab testing correctly identified all three samples which did not contain the stated API, resulting in 100% sensitivity and specificity for the Minilab in the identification of such falsified medicines in this study.

**Table 2:** Sensitivity and specificity of Global Pharma Health Fund Minilab testing for the prediction of the outcome of the compendial analysis according to U.S. Pharmacopeia 41: identity

		Compendial result			
		Fail	Complies	Total	
Minilab result	Fail	3	0	3	Sensitivity = $\frac{3}{3+0}$ = 100%
	Pass	0	448	448	Specificity = $\frac{448}{448+0}$ = 100%
	Total	3	448	451	

**Table 3:** Sensitivity and specificity of Global Pharma Health Fund Minilab testing for the prediction of the outcome of the compendial analysis according to U.S. Pharmacopeia 41: assay (= content of active pharmaceutical ingredient)

		Compendial result				Detection of any deviation (moderate or extreme)	Detection of extreme deviation
		Extreme deviation	Moderate deviation	Complies	Total		
Minilab result	Fail	6*	2	7	15*	Sensitivity = $\frac{6+2}{\left(6+2\right)+\left(8+24\right)}$ = 20%	Sensitivity = $\frac{6}{6+8}$ = 43%
	Pass	8	24	404	436	Specificity = $\frac{404}{404+7}$ = 98%	
	Total	14*	26	411	451*		

See text for definitions of moderate and extreme deviations.

* Includes the three falsified samples mentioned in Table 2.

**Table 4:** Sensitivity and specificity of Global Pharma Health Fund Minilab testing for the prediction of the outcome of the compendial analysis according to U.S. Pharmacopeia 41: Minilab disintegration testing versus compendial dissolution testing

		Compendial dissolution result				Detection of any deviation (moderate or extreme)	Detection of extreme deviation
		Extreme deviation	Moderate deviation	Complies	Total		
Minilab disintegration result	Fail	5	0	10	15	Sensitivity = $\frac{5+0}{\left(5+0\right)+\left(9+40\right)}$ = 9%	Sensitivity = $\frac{5}{5+9}$ = 36%
	Pass	9*	40	387	436*	Specificity = $\frac{387}{387+10}$ = 97%	
	Total	14*	40	397	451*		

See text for definitions of moderate and extreme deviations.

* Includes the three falsified samples mentioned in Table 2.

According to the GPHF Minilab manual,^15^ semiquantitative evaluation of TLC analysis is carried out by visual comparison of the spots of the sample with two spots of an authentic reference, representing 100% and 80% of the declared amount of the API, respectively. If the sample spot is considered weaker than the 80% reference spot, the sample is classified as failing and should be forwarded to confirmatory compendial analysis. Minilab testing is, therefore, not designed to detect moderate deviations from the declared API amount, that is, deviations by less than 20%. Indeed, as shown in Table 3, of 26 samples showing moderate deviations in compendial assay testing, only two had been reported to fail Minilab TLC testing. By contrast, of the 14 samples which showed extreme deviations in USP assay testing, six had been reported to fail Minilab TLC testing, resulting in 43% sensitivity of the Minilab in the detection of such medicines. Supplemental Table S2 lists all 15 samples reported to fail Minilab TLC analysis and the eight samples with extreme deviations which still were reported to pass Minilab TLC analysis, with their respective analytical results.

Testing for disintegration is a routine part of compendial medicine quality testing for solid oral dosage forms (e.g., tablets and capsules) and is performed using precisely defined equipment and conditions. The Minilab protocol includes a simplified testing method for disintegration which can be conducted without sophisticated equipment. Notably, disintegration testing measures a different endpoint than dissolution testing according to the USP. Therefore, a comparison of the results of Minilab disintegration testing with those of compendial dissolution testing is not possible in a strict sense. Nevertheless, it may still be of interest how well Minilab disintegration testing can predict the results of compendial dissolution testing. As was to be expected, the sensitivity of the Minilab in this comparison was low, that is, 9% (Table 4). The sensitivity increased to 36% if only extreme dissolution failures were considered.

In Tables 2–4, the values for specificity show the proportion of USP-compliant samples which were correctly predicted by the Minilab test as being compliant. Specificity resulted as 98% for assay and 97% for dissolution because the numbers of good-quality samples which were reported to fail Minilab analysis were low (seven samples in TLC testing and 10 samples in disintegration testing).

## DISCUSSION

### Prevalence of falsified and substandard medicines.

Of a total of 506 medicine samples collected in government and church health facilities, pharmacies, and informal vendors in Cameroon and the DR Congo, three samples (0.6%) were falsified, as evidenced by the absence of the stated API and, in two of these cases, by the presence of undeclared APIs (Figure 4). All other samples did contain the stated APIs, and visual inspection gave no indication of falsification. Obviously, a complete absence of falsified medicines must be aimed for. Nevertheless, the percentage of falsified medicines observed in this study is clearly lower than often portrayed in alarmist media reports about medicine quality in Africa. Our finding is in good accordance with the results of three large medicine quality studies in Africa, conducted by the WHO (QAMSA study),^13^ U.S. Pharmacopeial Convention,^40^ and ACT Consortium Drug Quality Program,^41^ which reported 0.2%, 0.3%, and 1.0% prevalence of falsified medicines, respectively. Two smaller studies conducted in Malawi and Togo by authors of the present article found 0.6%^42^ and 0.0%^31^ falsified medicines, respectively.

As noted in earlier studies, substandard medicines are much more frequently encountered than falsified medicines. In the present study, the percentage of medicines failing USP specifications for the assay (=content of API), for the dissolution of the API, and for the uniformity of the dosage units was 8.5%, 11.7%, and 6.1%, respectively. This is similar to the result of the WHO QAMSA study,^13^ which investigated antimalarial medicines in six African countries and reported failure rates in assay, dissolution, and uniformity of 10.9%, 15.0%, and 6.4%, respectively.

Overall, 18.6% of the medicine samples investigated in the present study did not comply with USP 41 specifications in one or several of the aforementioned three criteria, whereas 16.2% failed in the assay and/or the dissolution. This failure rate is in good agreement with the 18.7% estimate for the prevalence of SF medicines reported by Ozawa et al.^11^ from a meta-analysis of more than 40 medicine quality studies conducted in Africa. It is furthermore in reasonable agreement with the results of an authoritative review by the WHO^7^ which analyzed the results of 100 medicine quality studies, purposefully selected for their scientific quality. For studies which had used HPLC analysis (as also the present study did), that review reported an aggregated failure rate of 15.6% for medicine samples from LMICs. That review clearly stated that the included studies did not systematically test for dissolution, and our study showed that the failure rate almost doubles when dissolution is considered in addition to assay. Therefore, the reported rate of 15.6%^7^ estimated by the WHO must be expected to increase when dissolution is systematically included into the testing procedures.

As clearly visible from Figure 5, many samples which failed assay testing missed the pharmacopoeial limits only by a narrow margin. Although complete compliance of all medicines with the relevant specifications must be demanded, the public health risk posed by small deviations in the assay and/or the dissolution is probably low. Following the classification suggested by the WHO QAMSA study,^13^ we, therefore, differentiated between “moderate” and “extreme” deviations in assay and dissolution testing (see the Methods section for definitions). As depicted in Figures 5 and 6, and Supplemental Figures S12 and S13, overall 4.7% of the samples showed extreme deviations from the pharmacopoeial specifications (1.8% only in assay testing, 1.8% only in dissolution testing, and 1.2% simultaneously in assay and dissolution testing).

Figure 5 shows that except for the three falsified medicines, no sample was found to contain less than 50% of the declared content in assay testing. However, 13 of the 506 samples (2.6%) showed less than 50% dissolution of the API (in addition to the three falsified medicines). Also this observation emphasizes the importance of dissolution testing in medicine quality analysis.

### Subgroup analysis of the prevalence of SF medicines.

As explained in the Results section, we subsequently focus on the assay and dissolution results, that is, the most important criteria for the health effects of a medicine.^7^ Overall, the proportion of medicines which were out-of-specification in assay and/or dissolution was similar in Cameroon (17.2%) and the DR Congo (15.3%; *P* = 0.629) (Figure 3). However, as shown in Supplemental Figures S12 and S13, the number of samples with extreme deviations was clearly higher in Cameroon (7.8%) than in the DR Congo (1.9%; *P* = 0.0026). It is remarkable that in the northeast of the DR Congo, despite extreme poverty, political unrest, and disruptions by the Ebola epidemic, medicine quality is not worse but rather better than in the more affluent Cameroon.

As expected, medicine quality problems were most pronounced in informal vendors, with an out-of-specification rate of 28.2%. This rate was nearly identical in both countries and was significantly higher than in the three other types of outlets combined (12.3%; *P* < 0.0001). Notably, all three falsified medicines encountered in this study were sold by informal vendors, and also the rate of medicines with extreme deviations was significantly higher in informal vendors (11.3%) than in the three other categories of outlets (2.6%; *P* = 0.0003). Therefore, a well-enforced ban of medicine sales by informal vendors, as already implemented successfully in several East African countries, may represent a key intervention to reduce the problem of SF medicines.

In the DR Congo, the rate of out-of-specification medicines was similar in church health facilities (5.6%) and in government facilities (7.1%), and both values were significantly lower than that in informal vendors (28.1%; *P* = 0.0005 and *P* = 0.0099, respectively). None of the medicines in government or church health facilities showed extreme deviations. By contrast, private pharmacies showed an 18.7% failure rate, including 3.3% of medicines with extreme deviations. This indicates a lack of regulatory control of private pharmacies and their supply chains in the DR Congo.

In significant contrast to the high failure rate in medicines from private pharmacies in the DR Congo, Cameroon private pharmacies showed only a 5.7% failure rate (*P* = 0.018). Notably, of the 70 samples investigated from pharmacies in Cameroon, 47 were stated to be manufactured in Europe. As shown in our analysis of availability and prices,^22^ medicine prices in pharmacies in Cameroon were considerably higher than in other types of health facilities/outlets, and also much higher than in pharmacies in the DR Congo.

Medicines from church health facilities in Cameroon showed a 14.1% out-of-specification rate. Government health facilities in Cameroon showed an out-of-specification rate of 25.0%, similar to that found in medicines from informal vendors (28.4%). This indicates a need for improvements in medicine procurement and supply chain practices, especially of the government health services.

All authentic originator medicines investigated in this study were found to be within specifications. Of the 31 samples stated to be originator medicines, only the falsified Augmentin depicted in Figure 4 failed specifications. The failure rate of samples stated to be originator medicines was, therefore, 3%, significantly lower than the rate for (unbranded or branded) generic products (17.1%; *p* = 0.0431). Of the 506 samples investigated in this study, 78 were stated to be produced in Europe, including 30 originator medicines and 48 generic products. Of these, the falsified Augmentin and the falsified penicillin V depicted in Figure 4 failed specifications, and two branded generic products of amoxicillin/clavulanic acid which showed 88.6% of the declared amount of clavulanic acid and thereby narrowly missed the pharmacopoeial limit of 90%. The failure rate of medicines stated to be produced in Europe was, therefore, 5.1%, significantly lower than that of medicines stated to be produced in Asia (17.7%; *P* = 0.0049) and for medicines stated to be produced in Africa (22.2%; *P* = 0.0042). The difference between the medicines from Asia and Africa was not statistically significant (*P* = 0.385).

It must be emphasized that for many manufacturers from Asia and Africa, this study found most or all investigated samples to be in specifications (Supplemental Table S1). Notably, there were large manufacturers from India (e.g., Medopharm) or from China (e.g., CSPC Ouyi Pharmaceutical Co. Ltd., Shijiazhuang, China), represented by high numbers of samples in this study, whose out-of-specification rates were as low as those of the samples stated to be produced in Europe. On the other hand, there were some manufacturers, mostly represented by smaller numbers of samples in this study, with very high out-of-specification rates (see the Results section and Supplemental Table S1).

As noted in our analysis of the prices of medicines investigated in this study,^22^ medicines produced in Europe were much more expensive for the patients than medicines from Asia and Africa (i.e., nearly three times as expensive in Cameroon and nearly seven times as expensive in the DR Congo). Given the financial constraints in LMICs such as Cameroon and the DR Congo, restriction of procurement to medicines from countries with stringent regulatory authorities (i.e., mostly countries from Europe, North America, and Japan)^35^ may not be an affordable option. Rather, careful supplier qualification, that is, selection of manufacturers with a proven track record of providing good medicine quality is a key measure for quality assurance in medicine procurement. The WHO has established the Prequalification of Medicines Program to assist procurement agencies in the selection of good-quality products.^43,44^ Of the 13 types of medicines investigated in this study, three are included in the WHO Prequalification Program (ciprofloxacin, cotrimoxazole, and doxycycline). However, of 506 samples collected, only a single one (Ciplox-500^®^, Cipla, Mumbai, India) represented a WHO-prequalified product (and this was found to comply with USP specifications). To achieve a larger impact of the WHO Prequalification of Medicines Program on medicine quality in Cameroon and the DR Congo, a wider range of products may have to be included into the program, and in the procurement processes, more attention may have to be given to the selection of WHO-prequalified products.

Medicines against NCDs showed a 25% failure rate in assay and dissolution testing, significantly higher than that of antibiotics (12%; *P* = 0.0004). This difference was especially pronounced in the DR Congo (33% versus 9%; *P* < 0.0001) (Figure 3). This is alarming in view of the increasing burden of NCDs in LMICs.^45,46^ In an evaluation of cardiac drugs in different African countries, Antignac et al.^21^ also analyzed samples from the DR Congo, including atenolol, furosemide, hydrochlorothiazide, and four other cardiac medicines. They reported a prevalence of 26.7% poor-quality samples in the DR Congo, similar to the prevalence of 33.3% determined for NCD medicines in that country in the present study. Both the present survey and study by Antignac et al.^21^ found hydrochlorothiazide samples to be of good quality. As mentioned earlier, more than half of the hydrochlorothiazide samples found in the present survey represented the originator medicine Esidrex^®^ (Novartis), which were sold for very high prices in the DR Congo and Cameroon.^22^

Comparing the different storage recommendations on the packaging, medicines that carried a precise, WHO-recommended labeling statement, that is, either “Do not store above 30°C” or “Do not store above 25°C” showed a failure rate of 10%, significantly lower than those carrying a less precise recommendation or none at all (failure rate 27%; *P* < 0.0001). Possibly, suppliers giving attention to precise storage recommendations also give attention to other aspects of good manufacturing practice. However, medicines labeled “Do not store above 30°C” were not found to be better than those labeled “Do not store above 25°C” (8% versus 14% failure rate; *P* = 0.0999; not significant), indicating that this difference in labeling was not correlated with a relevant difference in quality and/or stability in the samples investigated in the present study.

### The GPHF Minilab as a screening tool for SF medicines.

Compendial (=pharmacopoeial) medicine analysis requires sophisticated equipment (usually HPLC) and highly trained personnel and, therefore, is expensive. In LMICs, the overall capacity for such analyses is limited. As a result, there is increasing worldwide interest in simple, inexpensive screening methods that will help in conducting larger post-marketing surveillance studies at an affordable cost.

So far, the most widely applied screening method in LMICs is the GPHF Minilab. The aforementioned review by the WHO,^7^ summarizing the result of 100 medicine quality studies, aggregated results for 48,218 samples. Of these, 20,010 had been investigated with the GPHF Minilab, and 5.0% of these had been reported to fail Minilab testing. By contrast, 19,809 samples had been investigated by HPLC, and 15.6% of these had been reported to fail this testing. These percentages are similar to the results of the present study, which found 6.7% of the investigated samples to fail Minilab analysis (which was carried out by local faith-based organizations in Cameroon and the DR Congo), compared with an overall 16.2% which failed the assay and/or dissolution testing according to USP41 (carried out at Tuebingen University, Tuebingen, Germany). Whereas the studies reviewed by the WHO^7^ mostly used only Minilab or only HPLC for analysis, the present study investigated 451 samples by both Minilab and HPLC, allowing a direct comparison of the results.

Minilab testing readily and reliably identified all three falsified medicines (Table 2). However, as mentioned in the Results section, Minilab is not designed to detect moderate deviations from the declared API amount, and this is clearly visible in the results shown in Table 3. Extreme deviations in API content were detected with a sensitivity of 43%.

As also explained in the Results section, disintegration and dissolution are different endpoints, and therefore, it is no surprise that samples failing USP dissolution testing were detected in the simple Minilab disintegration test with only 9% sensitivity. However, samples showing extreme dissolution failures in USP testing were detected by Minilab disintegration testing with 36% sensitivity (Table 4).

The sensitivity and specificity values determined in the present study should not be regarded as a final assessment of the analytical capacity of the Minilab because further improvements are certainly possible. For example, among the seven samples which Minilab TLC testing incorrectly reported as “failing” (Supplemental Table S2), three were cotrimoxazole samples reported to show a too weak spot of trimethoprim. Trimethoprim is the minor component of cotrimoxazole, besides the major component sulfamethoxazole. It is difficult to optimize TLC conditions in a way that allows a reliable estimation of the quantity of both components. Our study suggests that, if the trimethoprim spot appears too weak, the analysis should be repeated, applying a larger amount of both sample and reference. This, as well as a routine repetition of all “failed” Minilab analyses by another person or laboratory,^14^ is likely to further improve specificity. In addition, both sensitivity and specificity may be improved by quantification of TLC spot intensity with imaging software, for example, using a mobile phone app.^47^

Nevertheless, the results in Tables 2–4 show, besides the power of the Minilab in the detection of falsified medicines which do not contain the declared API, the limitations of the Minilab in the detection of quantitative deviations. This has also been noted in the WHO QAMSA study.^13^ The present study confirmed the well-known fact that Minilab testing cannot detect moderate deviations in medicine quality and observed that Minilab testing also missed a considerable number of samples with extreme deviations. As stated by the distributors of the Minilab,^48^ Minilab testing, therefore, should not be considered as a replacement for HPLC in the formal evaluation of pharmaceuticals. Rather, when compliance or noncompliance with compendial specifications is to be determined, compendial methods must be used. The value of screening methods, such as the Minilab, is primarily in studies in a low-resource environment, attempting to identify and eliminate as many falsified and grossly substandard medicines as possible with a limited budget. Samples failing Minilab analyses in such studies must subsequently be analyzed with compendial methods for confirmation. The rather high specificity of Minilab testing (Tables 2–4) ensures that the number of expensive compendial analyses to be performed remains limited. The costs of Minilab analyses and of compendial analyses have been estimated in two previous publications.^14,42^

Other simple and (more or less) inexpensive screening methods for medicine quality have recently been reviewed.^49^ As stated by the authors of that review, unfortunately, there is a lack of independent evaluations of most of these methods, particularly in field settings. Spectroscopic devices, especially using NIR and Raman spectroscopy, are attractive because of the ease and speed of handling. However, they require a complete library of spectra of all brands to be investigated. In the present study, which investigated only 13 medicines in only two countries, 260 different brands produced by 119 different manufacturers in 26 different countries were found. Creating and maintaining a complete library of reference spectra from such an assortment of brands and manufacturers is a formidable task, and its feasibility has yet to be demonstrated.

### Limitations of this study.

Although the sample size of our study is quite large in comparison with previous similar studies,^7,11^ the selection of medicines was limited to a small number of antibiotics and medicines against NCDs. Therefore, the results are not representative for other types of medicines, and further studies with other types of medicines, especially against NCDs, are required. In this study, two different sampling approaches had to be used: an overt approach in government and faith-based health facilities because these cannot sell a basket of prescription medicines to persons who are not patients in their facilities; and a mystery shopper approach in informal vendors (and in private pharmacies) because informal vendors would not be expected to agree to participate in a medicine quality study. Using an overt approach in government and faith-based health facilities may have potentially created a bias because staff may have preferably offered those medicines for collection which they considered to be of good quality. However, a meta-analysis by Ozawa et al.^11^ did not find evidence for a significant bias in studies with overt approaches as compared with studies with mystery shopper approaches. The selection of sampling sites was not strictly random, especially in the northeast of the DR Congo where only health zones could be included which were safe enough for travel by the study personnel. This may have led to an exclusion of health zones with potentially higher rates of SF medicines because political instability may restrict regulatory activities in these health zones.

## Supplemental tables and figures

Supplemental materials
