# Supplementary material for: Substandard and Falsified Antibiotics and Medicines against Noncommunicable Diseases in Western Cameroon and Northeastern Democratic Republic of Congo
Source: Am J Trop Med Hyg. 2020 May 11;103(2):894–908. doi: 10.4269/ajtmh.20-0184 (PMC7410427; doi:10.4269/ajtmh.20-0184)
Supplement: Supplementary file 1 [file tpmd200184.SD1.pdf]

# Supplementary PDF I:

## Contents

**Figure S1: HR - MS/MS Fragmentation pattern measured for the falsified penicillin V tablets QMCA035** ........ 2

**Figure S2: HR - MS/MS Fragmentation pattern measured for the falsified metronidazole tablets QMC266** ........ 2

**Figure S3: $^{1}$H NMR spectrum (400 MHz, $d_4$-MeOH) of the sample QMC266** ........ 3

........ 3

**Figure S4: $^{13}$C NMR spectrum (101 MHz, $d_4$-MeOH) of the sample QMC266** ........ 3

........ 3

**Figure S5: Edited $^{1}$H-$^{13}$C HSQC NMR spectrum (400 MHz, $d_4$-MeOH) of the sample QMC266** ........ 4

**Figure S6: $^{1}$H-$^{1}$H-COSY NMR spectrum (400 MHz, $d_4$-MeOH) of the sample QMC266** ........ 5

**Figure S7: $^{1}$H-$^{13}$C-HMBC NMR spectrum (400 MHz, $d_4$-MeOH) of the sample QMC266** ........ 6

........ 6

**Figure S8: $^{1}$H-$^{15}$N-HMBC NMR spectrum (400 MHz, $d_4$-MeOH) of the sample QMC266** ........ 7

**Figure S9: Superimposed $^{1}$H NMR spectra (400 MHz, $d_4$-MeOH) of a metronidazole benzoate standard and the sample QMC266** ........ 8

........ 8

**Figure S10: Superimposed $^{13}$C NMR spectra (101 MHz, $d_4$-MeOH) of a metronidazole benzoate standard and the sample QMC266** ........ 8

**Figure S11: NMR Results for metronidazole benzoate in sample QMC266 collected in the DR Congo** ........ 9

**Figure S12: Content of the active pharmaceutical ingredient determined for each sample, sorted by different categories** ........ 10

**Figure S13: Dissolution of the active pharmaceutical ingredient determined for each sample, sorted by different categories** ........ 11

**Figure S14: Frequency of non-compliance in Minilab TLC and disintegration testing in different subgroups of medicines** ........ 12

**Table S1: List of stated manufacturers of samples investigated in this study, and results for USP 41 assay and dissolution testing** ........ 13

**Table S2: List of samples reported to fail GPHF Minilab TLC analysis, and of samples reported to pass GPHF Minilab TLC analysis but showing extreme deviations in USP assay testing, with their respective USP assay results** ........ 18

**Table S3: Compendial quality results for the different products and batches as stated on the packaging** ........ 19

## Figure S1: HR - MS/MS Fragmentation pattern measured for the falsified penicillin V tablets QMCA035

HR - MS/MS Fragmentation pattern measured from the falsified penicillin V samples (QMCA035) collected in the Republic of Cameroon, actually containing acetaminophen (paracetamol) The exact *m/z* of the paracetamol parent ion measured was 152.0711 (calculated 152.0706).

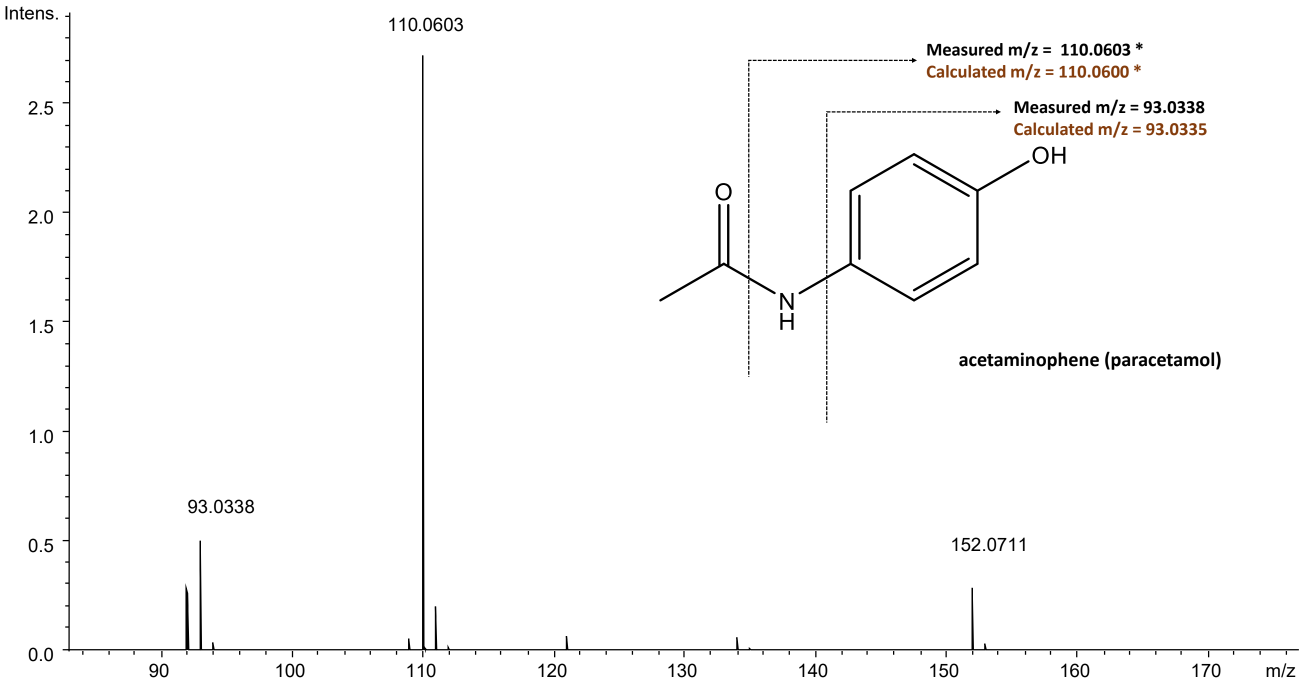


*the fragment ion with a *m/z* of 110, results from the protonated 4-aminophenol ($[H_2N\text{-}C_6H_4OH+H]^+$) formed by loss of ethone ($H_2C=C=O$).

## Figure S2: HR - MS/MS Fragmentation pattern measured for the falsified metronidazole tablets QMC266

HR - MS/MS Fragmentation pattern measured from the falsified metronidazole tablets (QMC266) collected in the DR Congo, actually containing metronidazole benzoate.

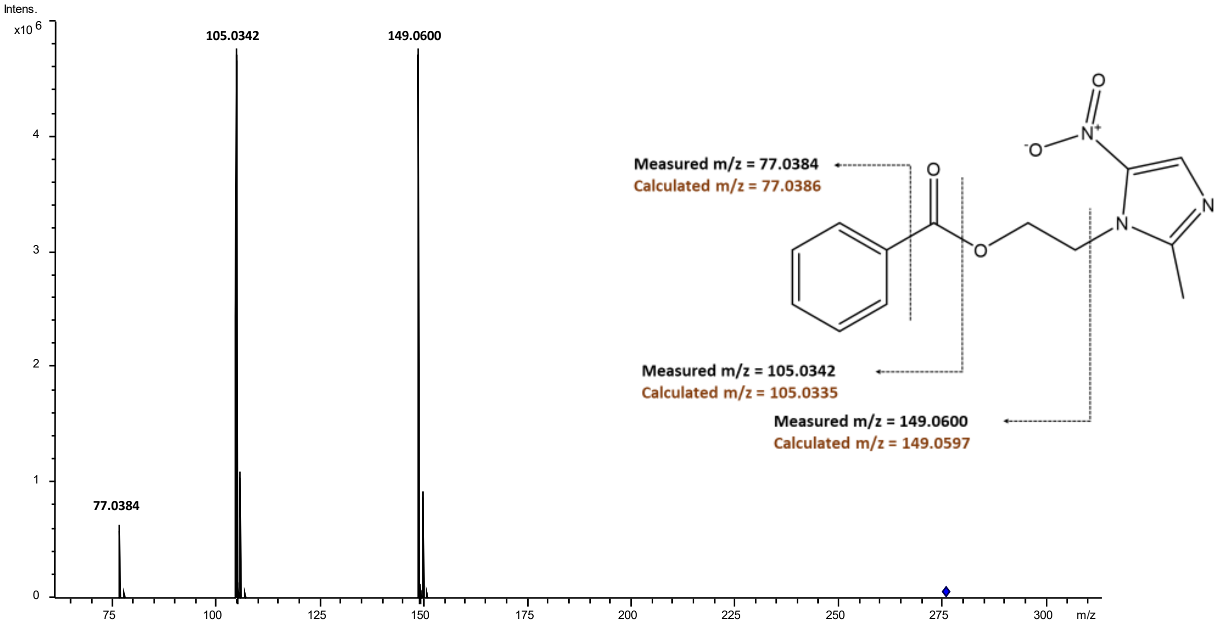

Figure S3: $^{1}$H NMR spectrum (400 MHz, $d_4$-MeOH) of the sample QMC266

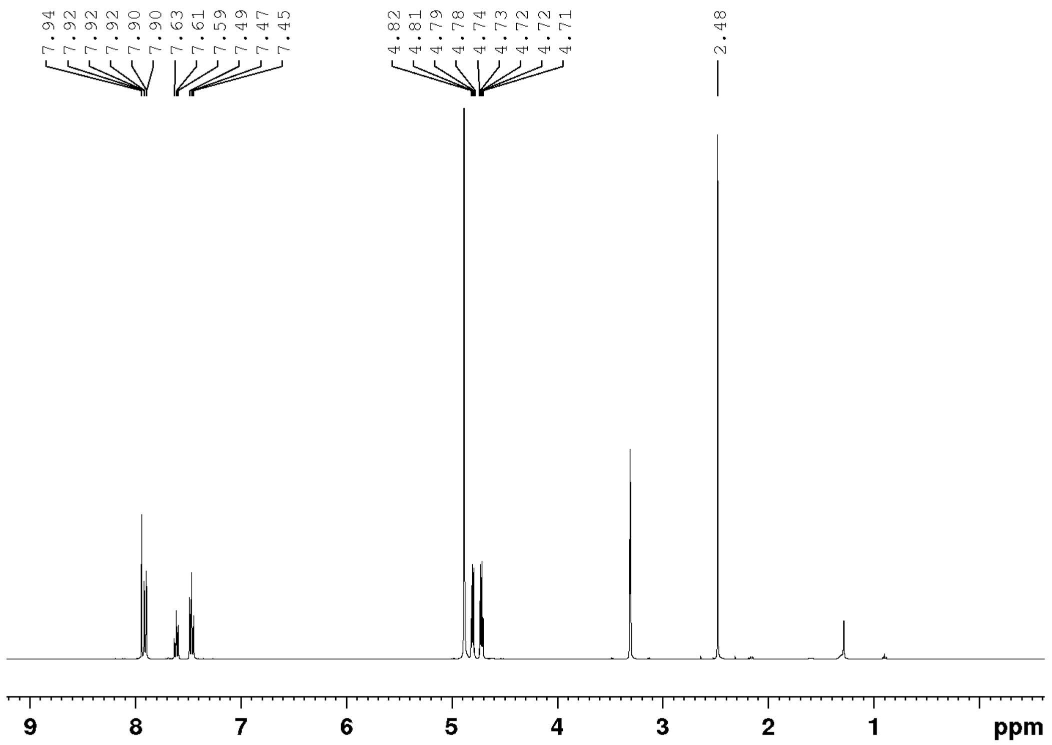


Figure S4: $^{13}$C NMR spectrum (101 MHz, $d_4$-MeOH) of the sample QMC266

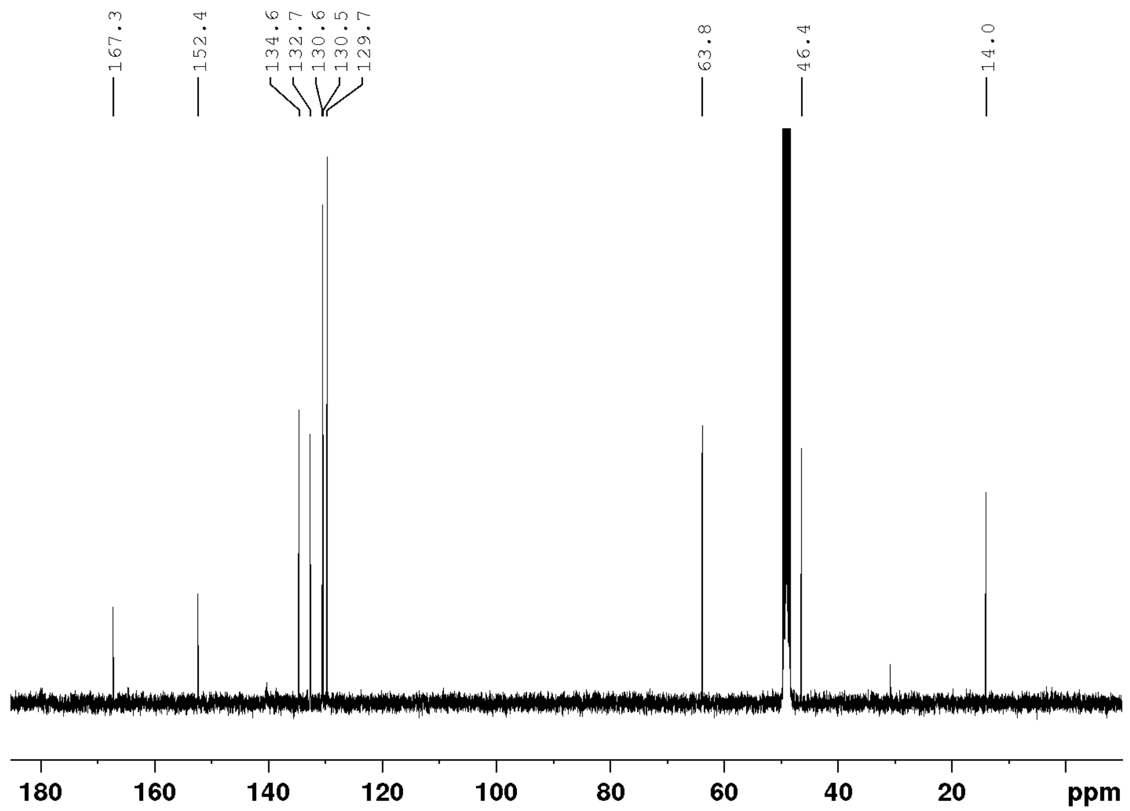

## Figure S5: Edited $^{1}H$-$^{13}C$ HSQC NMR spectrum (400 MHz, $d_4$-MeOH) of the sample QMC266

This experiment reveals which proton is directly bond to which carbon. Blue cross peaks indicate CH and $CH_3$ moieties, while red cross-peaks indicate $CH_2$-groups.

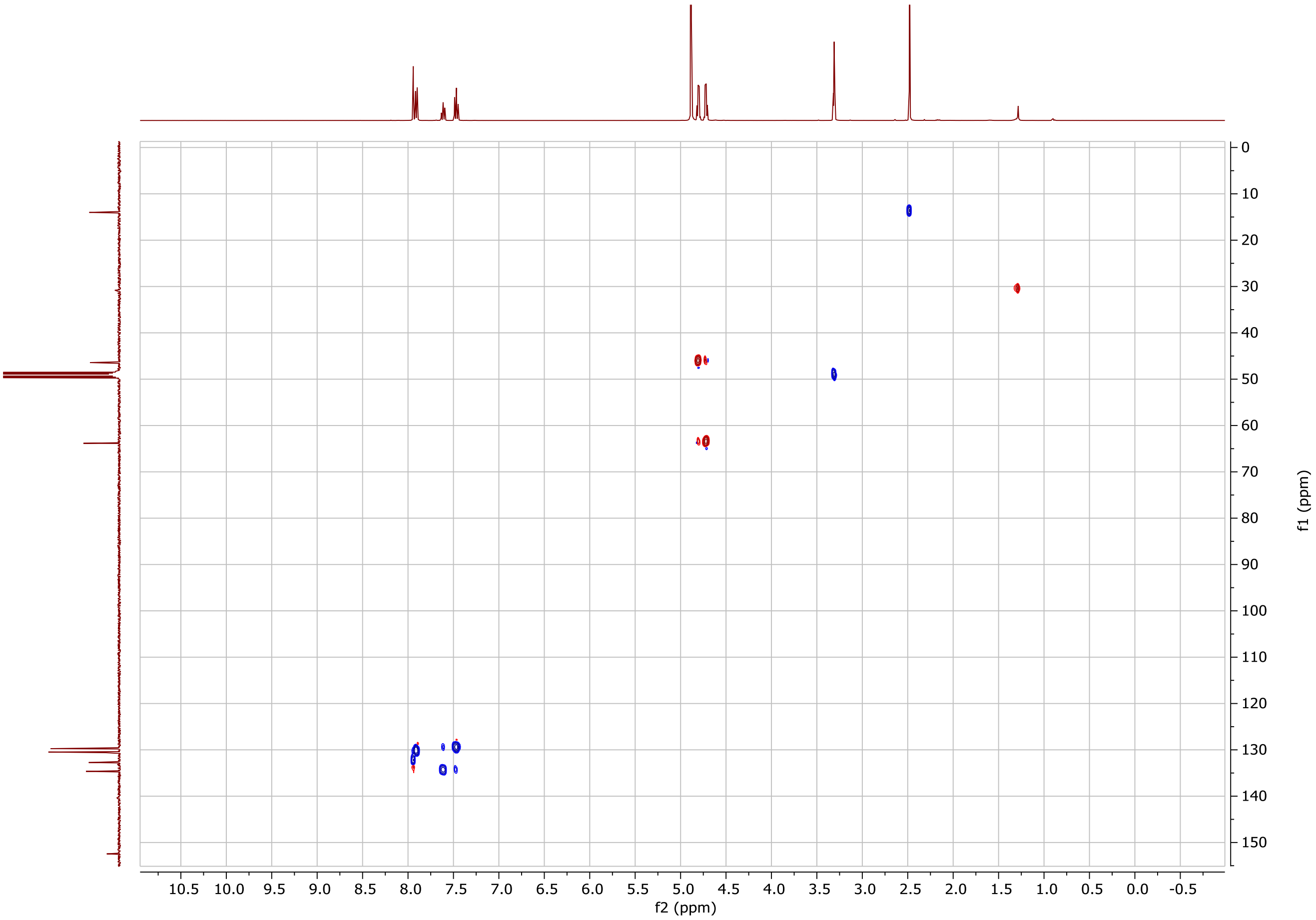

Figure S6: $^1$H-$^1$H-COSY NMR spectrum (400 MHz, $d_4$-MeOH) of the sample QMC266

Bold lines in the depicted chemical formula visualize the observed COSY-correlations.

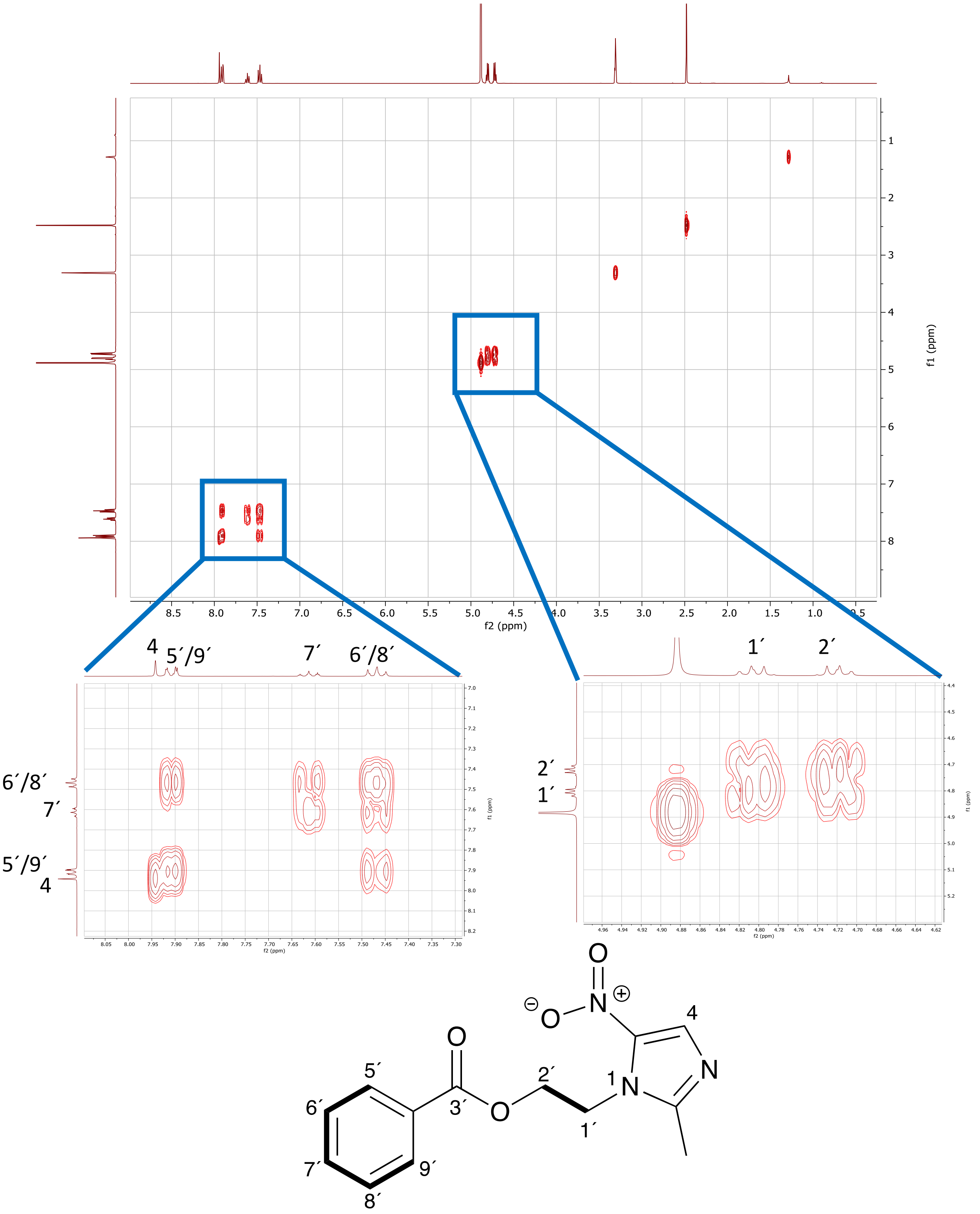

# Figure S7: $^1H$-$^{13}C$-HMBC NMR spectrum (400 MHz, $d_4$-MeOH) of the sample QMC266

Red arrows in the depicted chemical structure visualize the observed 2- and 3-bond HMBC long range correlations.

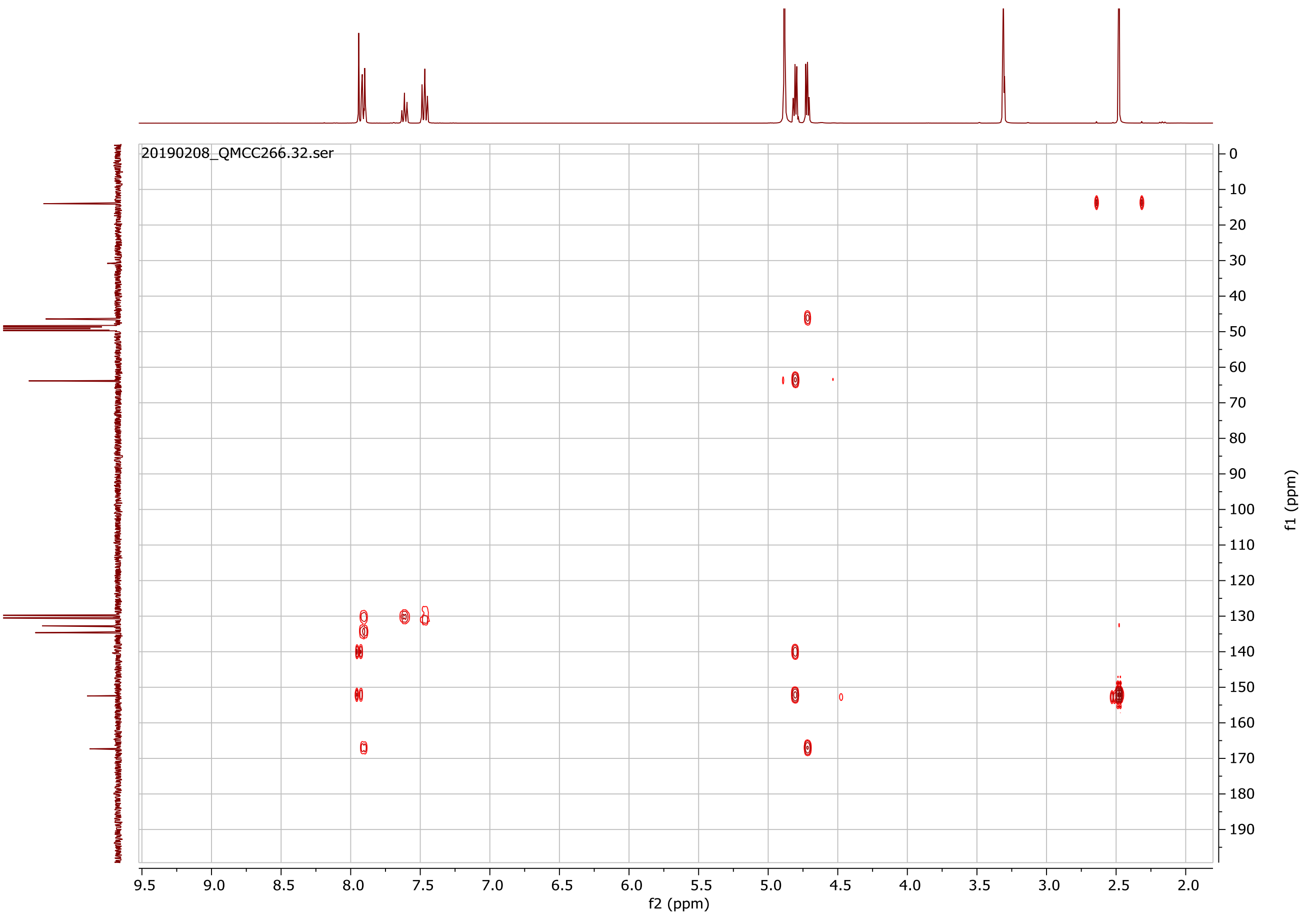


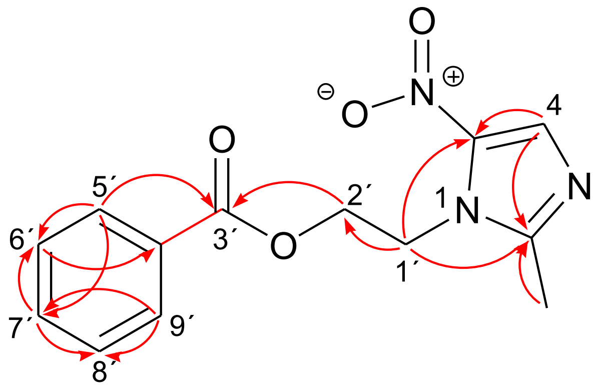

## Figure S8: $^{1}H$-$^{15}N$-HMBC NMR spectrum (400 MHz, $d_4$-MeOH) of the sample QMC266

Red arrows in the depicted chemical structure visualize the observed HMBC long range correlations.

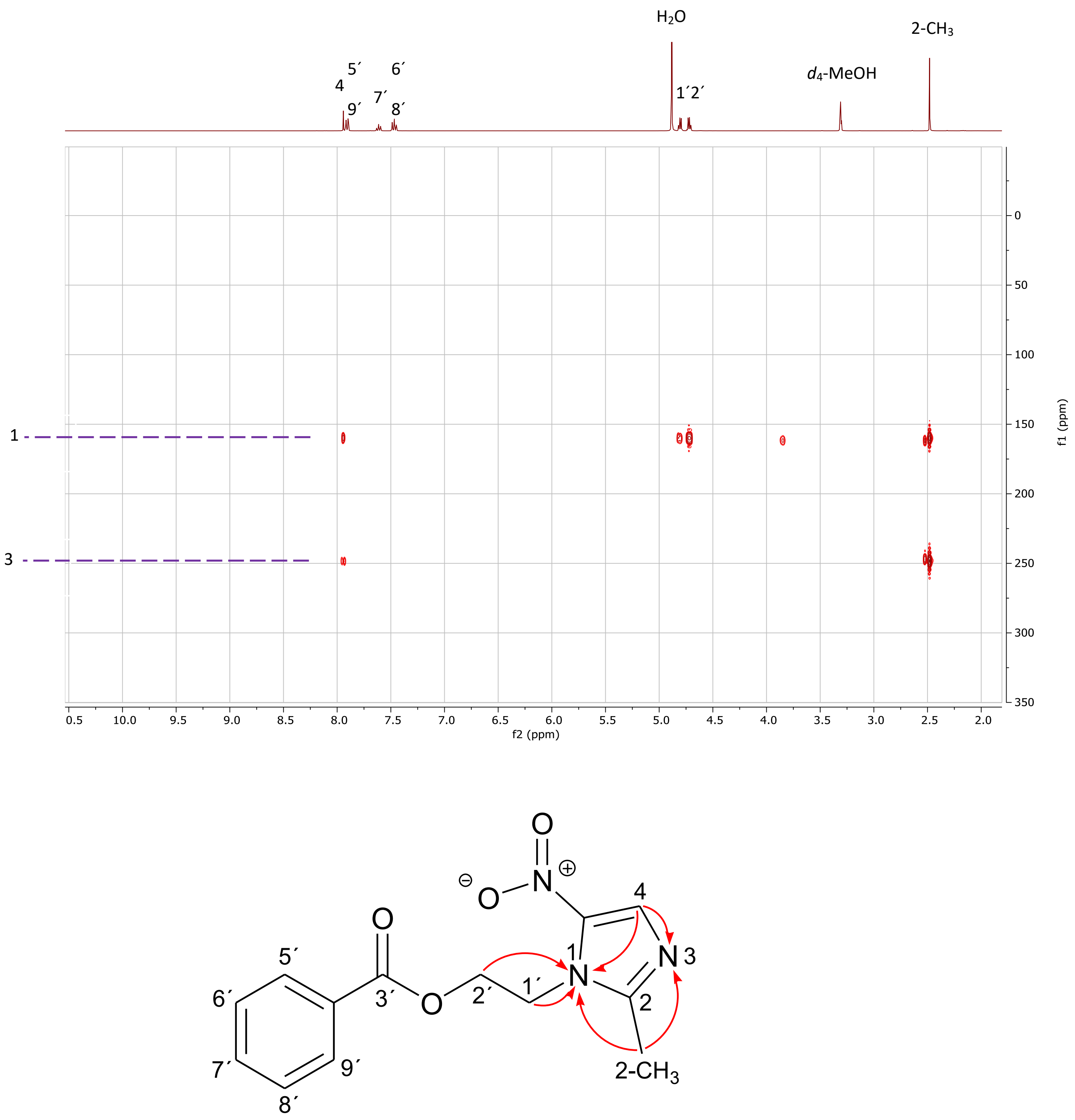

## Figure S9: Superimposed $^{1}$H NMR spectra (400 MHz, $d_4$-MeOH) of a metronidazole benzoate standard and the sample QMC266

metronidazole benzoate standard depicted in red (above) and the sample QMC266 depicted in blue (below).

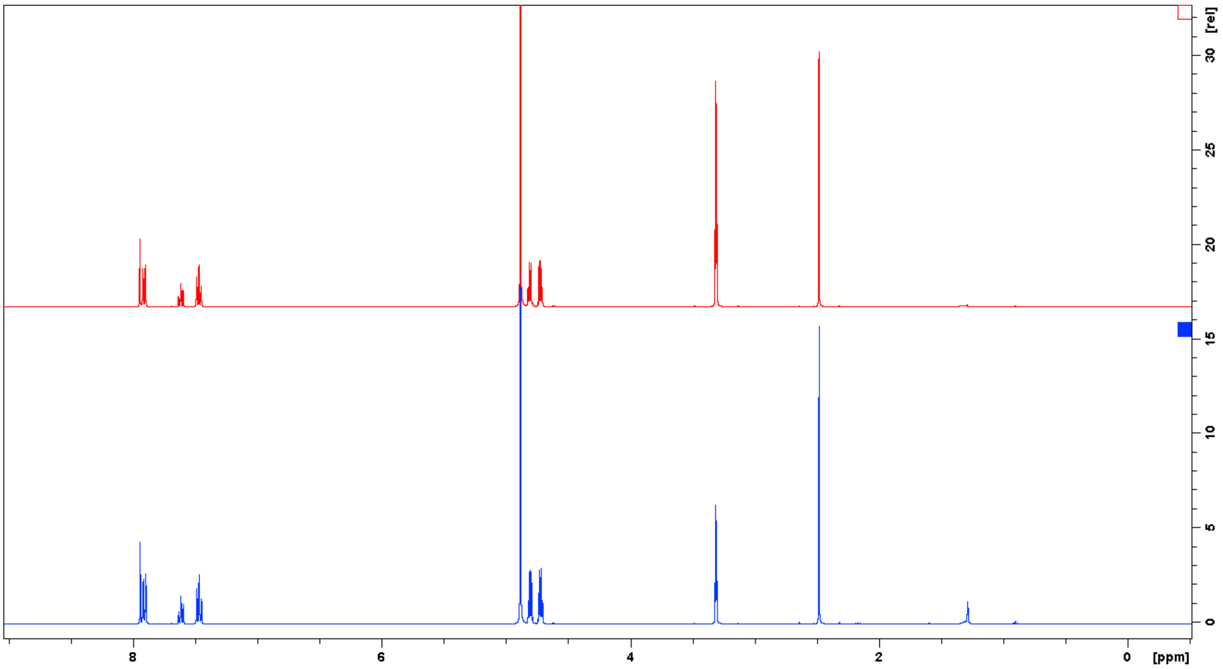


## Figure S10: Superimposed $^{13}$C NMR spectra (101 MHz, $d_4$-MeOH) of a metronidazole benzoate standard and the sample QMC266

metronidazole benzoate standard depicted in red (above) and the sample QMC266 depicted in blue (below).

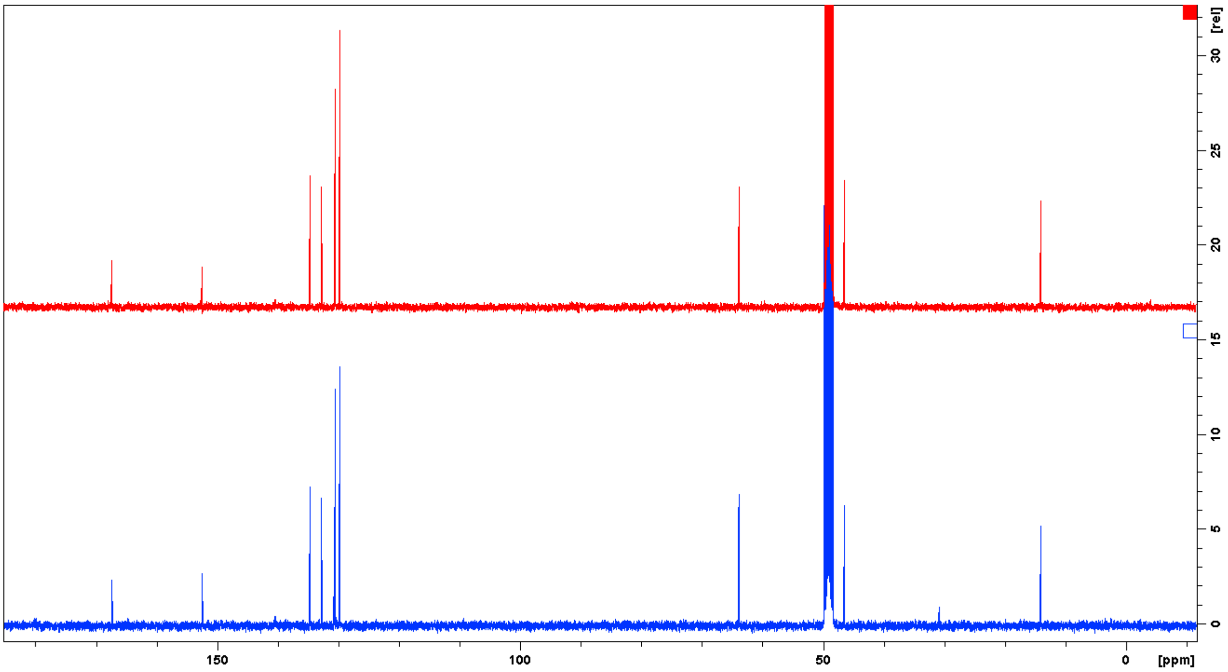

Figure S11: NMR Results for metronidazole benzoate in sample QMC266 collected in the DR Congo

| Position | $\delta_{C/N}$[a] | $\delta_H$ (integral, multiplicity)[b] | COSY | HMBC[c] |
|---|---|---|---|---|
| 1 | 160.0 $N_t$ | | | |
| 2 | 152.4 $C_q$ | | | |
| 2-$CH_3$ | 14.0 $CH_3$ | 2.48 (3H, s) | | 1, 2, 3 |
| 3 | 248.3 $N_t$ | | | |
| 4 | 132.7 CH | 7.94 (1H, s) | | 1, 2, 3, 5 |
| 5 | 140.5 $C_q$[d] | | | |
| 5-$NO_2$ | n.o.[e] | | | |
| 1' | 46.4 $CH_2$ | 4.81 (2H, m) | 2' | 1, 2', 2, 5 |
| 2' | 63.8 $CH_2$ | 4.72 (2H, m) | 1' | 1, 1', 3' |
| 3' | 167.3 CO | | | |
| 4' | 130.6 $C_q$ | | | |
| 5'/9' | 130.5 CH | 7.90+7.92 (2H, m) | 6', 8' | 3', 7', 5', 9' |
| 6'/8' | 129.7 CH | 7.47 (2H, m) | 5', 7', 9' | 4', 6', 8' |
| 7' | 134.6 CH | 7.61 (1H, m) | 6', 8' | 6´, 8' |

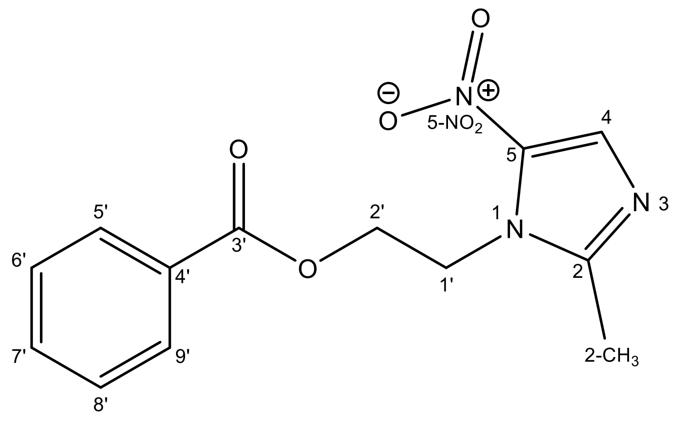


2'-(2-methyl-5-nitro-1*H*-imidazol-1-yl)ethyl benzoate

[a] Recorded at 101 MHz for $^{13}C$. $^{15}N$ NMR values were extracted from the corresponding $^{1}H$-$^{15}N$ HMBC NMR spectrum. Multiplicity determined by an edited $^{1}H$-$^{13}C$ HSQC and a DEPT135 NMR experiment.

[b] Recorded at 400 MHz.

[c] Protons showing long-range correlation with indicated carbon or nitrogen.

[d] $^{13}C$ NMR value was extracted from a $^{1}H$-$^{13}C$ HMBC NMR spectrum.

[e] Not observed.

Figure S12: Content of the active pharmaceutical ingredient determined for each sample, sorted by different categories

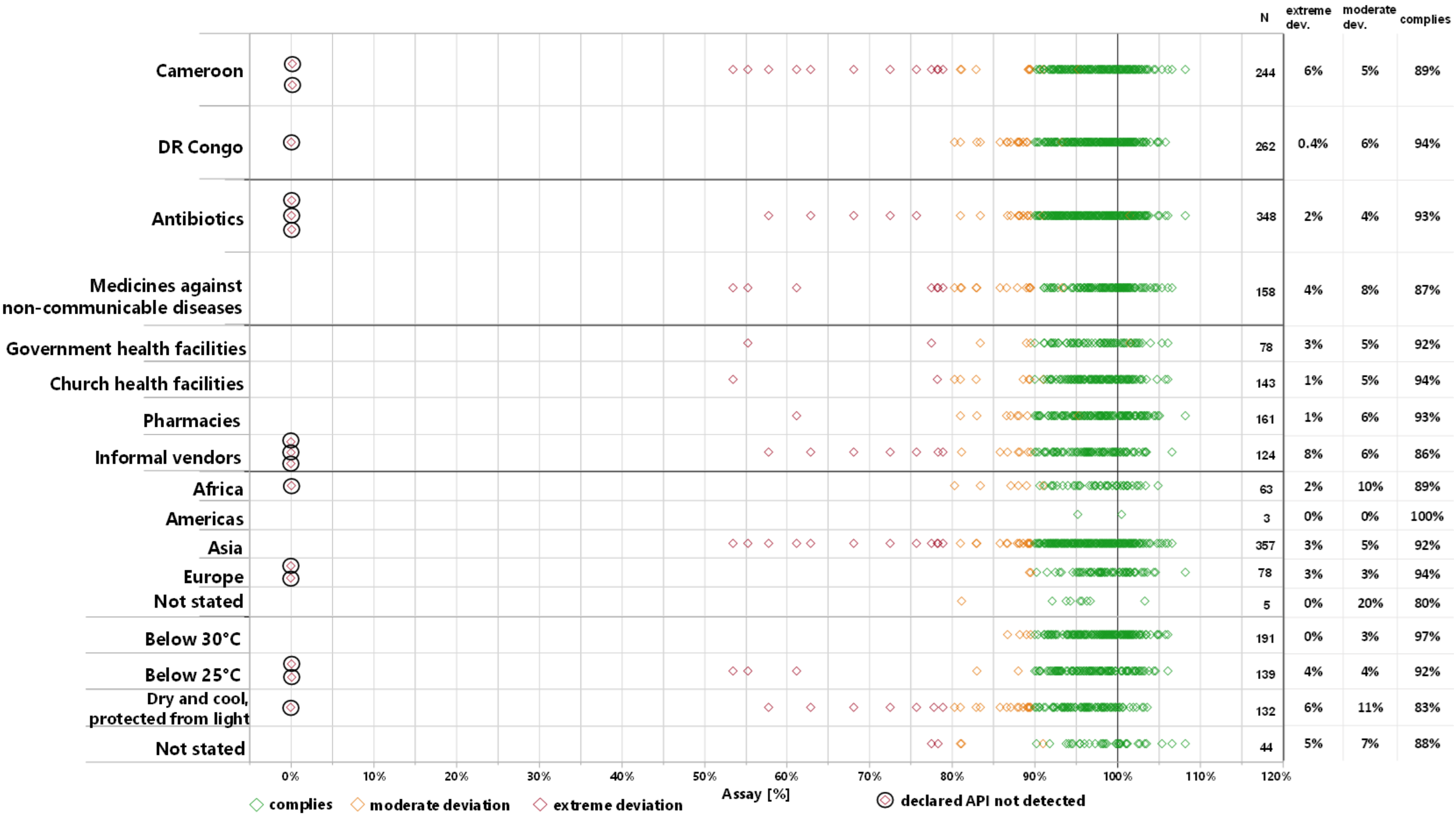

Figure S13: Dissolution of the active pharmaceutical ingredient determined for each sample, sorted by different categories

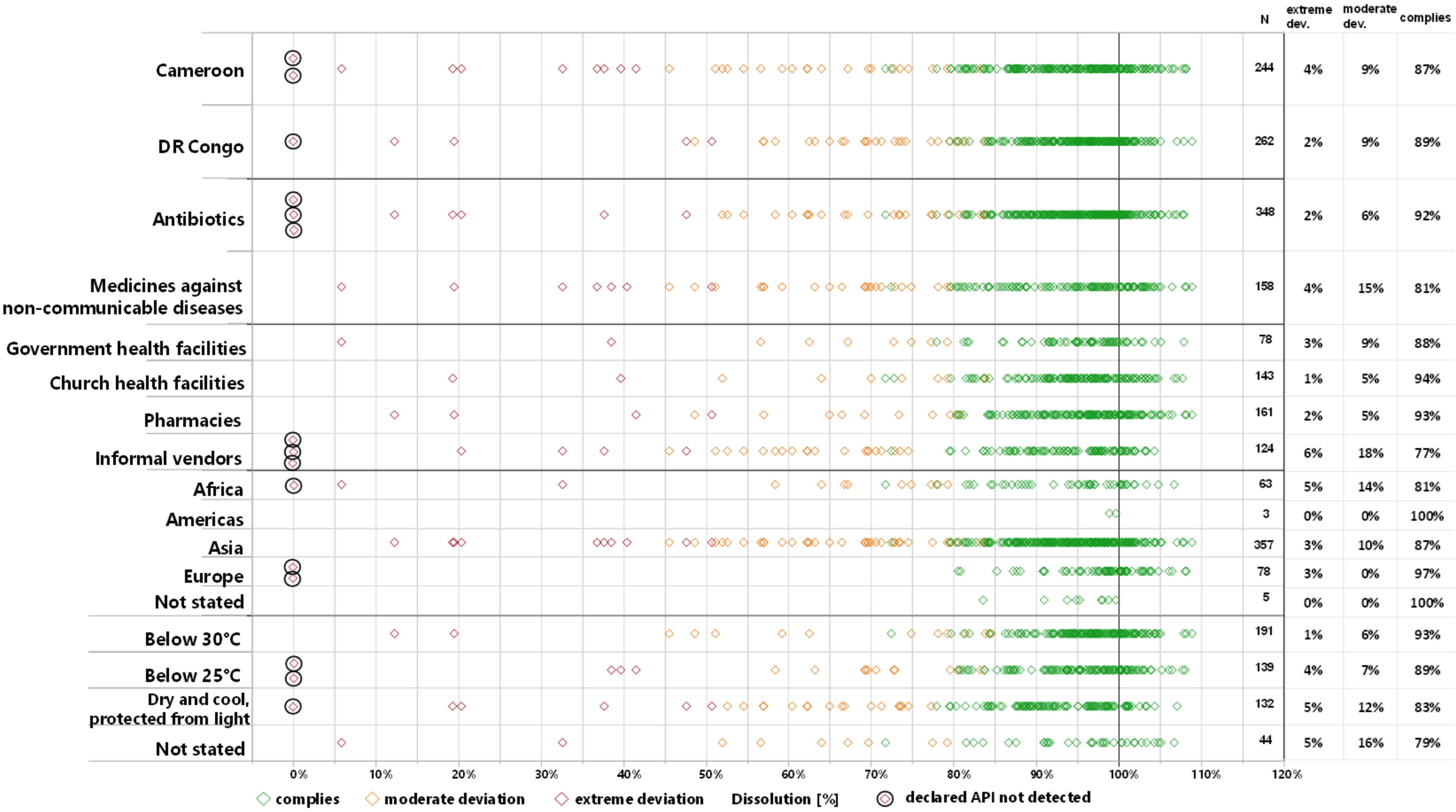

# Figure S14: Frequency of non-compliance in Minilab TLC and disintegration testing in different subgroups of medicines

| | | Cameroon | | | | | | DR Congo | | | | | |
|---|---|---|---|---|---|---|---|---|---|---|---|---|---|
| | | N total | non-compliant total N | non-compliant total [%] | non-compliant total 95% CI | only disintegration N | only TLC N | N total | non-compliant total N | non-compliant total [%] | non-compliant total 95% CI | only disintegration N | only TLC N |
| brand | Generics | 117 | 10 | 9% | [5-15] | 2 | 8 | 96 | 4 | 4% | [2-10] | 4 | 0 |
| | Branded | 108 | 11 | 10% | [6-17] | 6 | 5 | 102 | 3 | 3% | [1-8] | 2 | 1 |
| | Originator | 18 | 2 | 11% | [3-33] | 1 | 1 | 10 | 0 | 0% | | 0 | 0 |
| continent of origin | Africa | 23 | 4 | 17% | [8-42] | 2 | 2 | 30 | 1 | 3% | [1-16] | 0 | 1 |
| | Americas | 3 | 0 | 0% | | 0 | 0 | | | | | | |
| | Asia | 155 | 12 | 8% | [4-13] | 4 | 8 | 162 | 6 | 4% | [2-8] | 6 | 0 |
| | Europe | 57 | 6 | 11% | [6-23] | 3 | 3 | 16 | 0 | 0% | | 0 | 0 |
| | Not stated | 5 | 1 | 20% | [2-44] | 0 | 1 | | | | | | |
| site type | Government health facilities | 36 | 4 | 11% | [4-25] | 0 | 4 | 42 | 3 | 7% | [3-19] | 3 | 0 |
| | Church health facilities | 71 | 4 | 6% | [2-14] | 2 | 2 | 40 | 0 | 0% | | 0 | 0 |
| | Pharmacies | 70 | 7 | 10% | [5-19] | 4 | 3 | 70 | 1 | 1% | [0-8] | 1 | 0 |
| | Informal vendors | 66 | 8 | 12% | [6-22] | 3 | 5 | 56 | 3 | 5% | [2-15] | 2 | 1 |
| indication | Antibiotics | 151 | 14 | 9% | [6-15] | 5 | 9 | 157 | 6 | 4% | [2-8] | 5 | 1 |
| | MANCD | 92 | 9 | 10% | [5-18] | 4 | 5 | 51 | 1 | 2% | [0-10] | 1 | 0 |
| store at | Below 25 °C | 89 | 10 | 11% | [6-20] | 4 | 6 | 43 | 1 | 2% | [0-12] | 0 | 1 |
| | Below 30°C | 51 | 2 | 4% | [1-13] | 1 | 1 | 102 | 5 | 5% | [2-11] | 5 | 0 |
| | Dry and cool, protected from light | 61 | 6 | 10% | [5-20] | 3 | 3 | 61 | 1 | 2% | [0-9] | 1 | 0 |
| | Not stated | 42 | 5 | 12% | [5-25] | 1 | 4 | 2 | 0 | 0% | | 0 | 0 |
| active pharmaceutical ingredient | Amoxicillin and Clavulanic acid | 19 | 1 | 5% | [1-25] | 0 | 1 | 11 | 0 | 0% | | 0 | 0 |
| | Amoxicillin | 24 | 1 | 4% | [1-20] | 0 | 1 | 25 | 0 | 0% | | 0 | 0 |
| | Ciprofloxacin | 25 | 1 | 4% | [1-20] | 0 | 1 | 26 | 0 | 0% | | 0 | 0 |
| | Doxycycline | 22 | 0 | 0% | | 0 | 0 | 20 | 2 | 10% | [2.8-30] | 2 | 0 |
| | Metronidazole | 25 | 4 | 16% | [6-35] | 4 | 0 | 27 | 4 | 15% | [6-33] | 3 | 1 |
| | Penicillin V | 13 | 2 | 15% | [4-42] | 0 | 2 | 21 | 0 | 0% | | 0 | 0 |
| | Sulfamethoxazole and Trimethoprim | 23 | 5 | 22% | | 1 | 4 | 27 | 0 | 0% | | 0 | 0 |
| | Atenolol | 0 | 0 | 0% | | 0 | 0 | 5 | 1 | 20% | [4-62] | 1 | 0 |
| | Furosemide | 17 | 0 | 0% | | 0 | 0 | 18 | 0 | 0% | | 0 | 0 |
| | Glibenclamide | 19 | 0 | 0% | | 0 | 0 | 0 | 0 | 0% | | 0 | 0 |
| | Hydrochlorothiazide | 21 | 0 | 0% | | 0 | 0 | 4 | 0 | 0% | | 0 | 0 |
| | Metformin | 20 | 4 | 20% | [8-42] | 4 | 0 | 10 | 0 | 0% | | 0 | 0 |
| | Salbutamol | 15 | 5 | 33% | [15-58] | 0 | 5 | 14 | 0 | 0% | | 0 | 0 |
| | **Overall** | **243** | **23** | **9%** | **[6.4-13.8]** | **9** | **14** | **208** | **7** | **3%** | **[1.6-6.8]** | **6** | **1** |

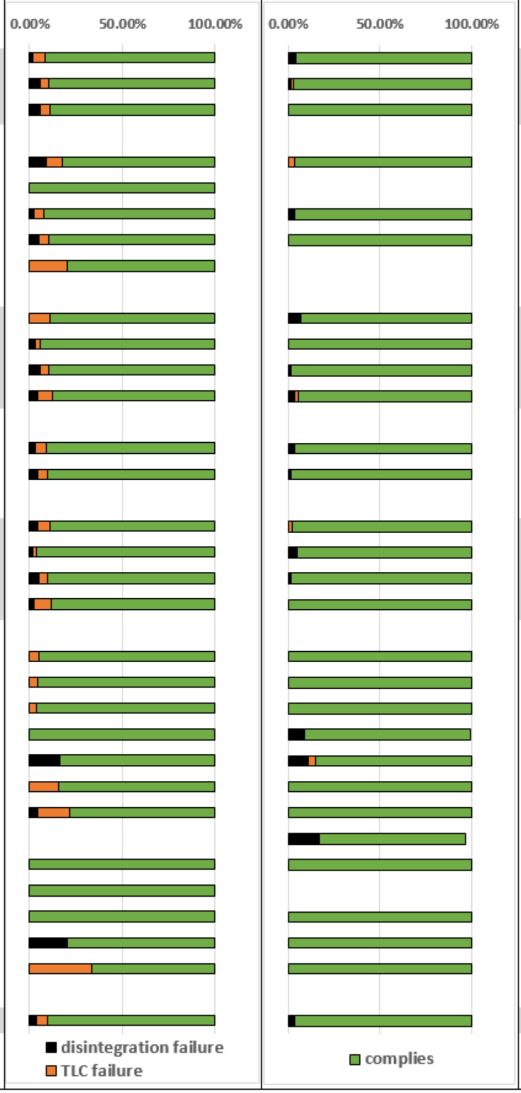


TLC= thin-layer chromatography

Table S1: List of stated manufacturers of samples investigated in this study, and results for USP 41 assay and dissolution testing

| Stated Continent of Origin | Stated Country of Origin | Stated Manufacturer | N | complies | moderate deviation | extreme deviation |
|---|---|---|---|---|---|---|
| **Africa** | Benin | Pharmaquick | 7 | 5 | 0 | 2 |
| | Burundi | Société industrielle Pharmaceutique (SIPHAR) | 1 | 1 | 0 | 0 |
| | Cameroon | Africure Pharmaceuticals Cameroon S.A. | 2 | 1 | 1 | 0 |
| | | Cinpharm ** | 3 | 2 | 1 | 0 |
| | DR Congo | Phatkin B.P. | 5 | 2 | 3 | 0 |
| | | Zenufa Laboratoire | 4 | 3 | 1 | 0 |
| | Ghana | Entrance Pharmaceuticals & Research Centre | 5 | 3 | 2 | 0 |
| | Kenya | Cosmos Limited | 1 | 1 | 0 | 0 |
| | | DAWA Limited | 7 | 6 | 1 | 0 |
| | | Elys Chemical Industries Ltd. | 3 | 2 | 1 | 0 |
| | | Laboratory & Allied Ltd. | 2 | 2 | 0 | 0 |
| | | MAC'S Pharmaceuticals Ltd. | 2 | 1 | 0 | 1 [§] |
| | | Pharmaceutical Manufacturing Co. Ltd. | 1 | 0 | 1 | 0 |
| | | Regal Pharmaceuticals Ltd. | 5 | 5 | 0 | 0 |
| | Nigeria | New Divine Favour Pharmaceutical Industries Ltd. | 1 | 1 | 0 | 0 |
| | Senegal | Wintrop Pharma Sénégal Group SANOFI | 1 | 1 | 0 | 0 |
| | Togo | Sprukfield | 4 | 4 | 0 | 0 |
| | Uganda | Kampala Pharmaceutical Industries | 7 | 7 | 0 | 0 |
| | Uganda | Rene Industries Ltd. | 2 | 2 | 0 | 0 |
| | **subtotal** | | **63** | **49** | **11** | **3** |
| **Americas** | British West Indies | Prost Pharma (France) | 2 | 2 | 0 | 0 |
| | USA | Sandoz | 1 | 1 | 0 | 0 |
| | **subtotal** | | **3** | **3** | **0** | **0** |

| Stated Continent of Origin | Stated Country of Origin | Stated Manufacturer | N | complies | moderate deviation | extreme deviation |
|---|---|---|---|---|---|---|
| **Asia** | China | Anhui Chengshi Pharmaceutical Co. Ltd | 1 | 1 | 0 | 0 |
| | | Anhui Medipharm Co. Ltd. | 1 | 0 | 1 | 0 |
| | | Chifeng Wanze Pharmaceutical Co. Ltd. | 1 | 1 | 0 | 0 |
| | | CSPC Ouyi Pharmaceutical Co. Ltd. | 22 | 21 | 1 | 0 |
| | | CSPC Zhongnuo Pharmaceuticals Co. Ltd. | 10 | 10 | 0 | 0 |
| | | Farmasino Pharmaceutical Co. Ltd | 6 | 5 | 1 | 0 |
| | | Greenfield Pharmaceuticals (Jiang Su) Co. Ltd. | 1 | 1 | 0 | 0 |
| | | Guilin Pharmaceutical Co. Ltd. | 4 | 4 | 0 | 0 |
| | | Jiangsu Pengyao Pharmaceutical Co. Ltd. | 2 | 2 | 0 | 0 |
| | | Jiangsu Ruinian Qianjin Pharm. Co.Ltd | 4 | 4 | 0 | 0 |
| | | Jiangxi Xier Kangtai Pharmaceutical Co. Ltd. | 5 | 3 | 2 | 0 |
| | | Jinzhou Jiuyang Pharmaceutical Co. Ltd | 3 | 2 | 0 | 1 |
| | | JSPY Pharmaceutical Co. Ltd. | 3 | 3 | 0 | 0 |
| | | Nanjing Baijingyu Pharmaceutical Co. Ltd. | 3 | 3 | 0 | 0 |
| | | Nanjing Sino Pharmaceutical Ltd. | 1 | 1 | 0 | 0 |
| | | Ningbo Shuangwei Pharmaceutical Co. Ltd | 6 | 6 | 0 | 0 |
| | | North China Pharmaceutical Co. Ltd. *** | 8 | 8 | 0 | 0 |
| | | Reyoung Pharmaceutical Co. Ltd. | 9 | 9 | 0 | 0 |
| | | Shandong Shenglu Pharmaceutical Co. Ltd | 4 | 0 | 0 | 4 |
| | | Shandong Xier Kangtai Pharm Co. Ltd | 1 | 1 | 0 | 0 |
| | | Shandong Yikang Pharmaceutical Co. Ltd. | 2 | 1 | 1 | 0 |
| | | Shanghai Juchen Import and Exports Co. Ltd. | 4 | 4 | 0 | 0 |
| | | Shanxi Lianbang Pharmaceutical Co. Ltd. | 2 | 2 | 0 | 0 |

| Stated Continent of Origin | Stated Country of Origin | Stated Manufacturer | N | complies | moderate deviation | extreme deviation |
|---|---|---|---|---|---|---|
| | | Sinochem Jiangsu Co. Ltd | 14 | 8 | 3 | 3 |
| | | Sishui xier Kang Pharmaceutical Co. Ltd | 1 | 0 | 0 | 1 |
| | | Yanzhou Xierkangtai pharmaceutical Co. Ltd. | 3 | 2 | 1 | 0 |
| | Hong Kong | Hongkong Prost Medicines and Health Products Co. Ltd | 3 | 3 | 0 | 0 |
| | India | Agog Pharma Ltd. | 4 | 4 | 0 | 0 |
| | | Alkem Laboratories Ltd. | 1 | 0 | 1 | 0 |
| | | Arco Pharma Pvt. Ltd | 7 | 1 | 6 | 0 |
| | | Asence Pharma Pvt. Ltd. | 6 | 4 | 2 | 0 |
| | | Astra Lifecare Pvt. Ltd. | 11 | 9 | 0 | 2 |
| | | Aura pharmaceuticals Pvt. Ltd | 9 | 5 | 4 | 0 |
| | | Aurobindo Pharma Ltd. | 1 | 0 | 1 | 0 |
| | | Axon Drugs Pvt. Ltd. | 1 | 1 | 0 | 0 |
| | | Bliss GVS Pharma Ltd. | 1 | 0 | 1 | 0 |
| | | Cadila Healthcare Ltd. | 1 | 1 | 0 | 0 |
| | | Cipla Ltd. | 1 | 1 | 0 | 0 |
| | | Ciron Drugs and Pharmaceuticals Ltd. | 2 | 2 | 0 | 0 |
| | | Combitic Global Caplet Pvt. Ltd. | 1 | 1 | 0 | 0 |
| | | Fourrts | 3 | 3 | 0 | 0 |
| | | Global Pharma Healthcare Pvt. Ltd. | 4 | 4 | 0 | 0 |
| | | Holden Medical Laboratories Pvt. Ltd. | 3 | 3 | 0 | 0 |
| | | Intermed | 1 | 1 | 0 | 0 |
| | | Ipca Laboratories Ltd. | 1 | 1 | 0 | 0 |
| | | J. B. Chemicals and Pharmaceuticals Ltd. | 1 | 1 | 0 | 0 |
| | | Kopran Limited | 2 | 2 | 0 | 0 |
| | | Leben Laboratories Pvt. Ltd | 1 | 1 | 0 | 0 |
| | | Lincoln Pharmaceuticals Ltd. | 8 | 8 | 0 | 0 |
| | | Lord Lifescience Pvt. Ltd. | 1 | 0 | 1 | 0 |
| | | Macleods Pharmaceuticals Ltd. | 4 | 4 | 0 | 0 |
| | | Mancare pharmaceutical Ltd | 4 | 4 | 0 | 0 |

| Stated Continent of Origin | Stated Country of Origin | Stated Manufacturer | N | complies | moderate deviation | extreme deviation |
|---|---|---|---|---|---|---|
| | | Maneesh Pharmaceuticals Ltd | 1 | 1 | 0 | 0 |
| | | Maxheal Laboratories Pvt. Ltd. | 2 | 1 | 1 | 0 |
| | | Maxtar Bio-Genics | 14 | 9 | 2 | 3 * |
| | | Medicamen Biotech Ltd. | 8 | 7 | 1 | 0 |
| | | Medicef Pharma | 4 | 4 | 0 | 0 |
| | | Medico Remedies Pvt. Ltd. | 5 | 0 | 1 | 4 |
| | | Medley Pharmaceuticals Ltd. | 5 | 5 | 0 | 0 |
| | | Medopharm Pvt. Ltd. | 41 | 39 | 2 | 0 |
| | | Mepro Pharmaceuticals Pvt. Ltd. | 2 | 2 | 0 | 0 |
| | | Micro Labs Ltd. | 4 | 4 | 0 | 0 |
| | | Milan Laboratories (India) Pvt. Ltd | 6 | 5 | 1 | 0 |
| | | Nem Laboratories Pvt. Ltd. | 1 | 1 | 0 | 0 |
| | | not stated | 1 | 1 | 0 | 0 |
| | | Osaka Pharmaceuticals Pvt. Ltd. | 3 | 1 | 2 | 0 |
| | | PIL Pharmaceuticals Pvt. Ltd. | 1 | 1 | 0 | 0 |
| | | Prashi Pharma Pvt. Ltd | 6 | 1 | 4 | 1 |
| | | Shalina Laboratories Pvt. Ltd. | 1 | 1 | 0 | 0 |
| | | Sparsh Bio-Tech Pvt. Ltd. | 7 | 7 | 0 | 0 |
| | | Strides Arcolab Limited | 23 | 21 | 2 | 0 |
| | | Strides Shasun Limited | 11 | 9 | 2 | 0 |
| | | Triveni Formulations Limited | 1 | 1 | 0 | 0 |
| | | Ultra Care International | 2 | 2 | 0 | 0 |
| | | UMEDICA Laboratories | 1 | 1 | 0 | 0 |
| | | Zee Laboratories | 1 | 1 | 0 | 0 |
| | | ZIM Laboratories Ltd. | 1 | 1 | 0 | 0 |
| | Sultanat of Oman | National pharmaceutical industries | 1 | 1 | 0 | 0 |
| | Turkey | Bilim Pharmaceuticals | 1 | 1 | 0 | 0 |
| | **subtotal** | | **357** | **294** | **44** | **19** |
| **Europe** | Austria | Sandoz | 10 | 10 | 0 | 0 |
| | Belgium | Merck | 3 | 3 | 0 | 0 |
| | | Oxford Pharma | 1 | 0 | 0 | 1 § |
| | Cyprus | Medochemie Ltd. | 2 | 2 | 0 | 0 |

| Stated Continent of Origin | Stated Country of Origin | Stated Manufacturer | N | complies | moderate deviation | extreme deviation |
|---|---|---|---|---|---|---|
| | | Remedica Ltd | 2 | 2 | 0 | 0 |
| | France | Famar Lyon | 1 | 1 | 0 | 0 |
| | | Glaxo Welcome Production | 3 | 3 | 0 | 0 |
| | | Laboratoires Bailleul | 1 | 1 | 0 | 0 |
| | | Laboratoire Bailly-Creat | 6 | 6 | 0 | 0 |
| | | Sanofi-Winthrop Industrie | 6 | 6 | 0 | 0 |
| | Germany | Aspen Bad Oldesloe GmbH | 1 | 1 | 0 | 0 |
| | | Berlin Chemie | 1 | 1 | 0 | 0 |
| | | Denk Pharma GmbH & Co. KG | 12 | 10 | 2 | 0 |
| | | Salutas Pharma GmbH | 2 | 2 | 0 | 0 |
| | Italy | Errekappa Euroterapici S.p.A | 1 | 1 | 0 | 0 |
| | | Laboratori Guidotti S.p.A | 1 | 1 | 0 | 0 |
| | Spain | Ferrer Internacional S.A. | 3 | 3 | 0 | 0 |
| | | Novartis Farmacéutica S.A. | 14 | 14 | 0 | 0 |
| | Sweden | Bluefish Pharmaceuticals AD | 1 | 1 | 0 | 0 |
| | United Kingdom | SmithKline Beecham Pharmaceuticals | 1 | 0 | 0 | 1 § |
| | | Sonmart Pharma (UK) | 6 | 6 | 0 | 0 |
| | **subtotal** | | **78** | **74** | **2** | **2** |
| **not stated** | not stated | Cinpharm ** | 3 | 3 | 0 | 0 |
| | | not stated | 2 | 1 | 1 | 0 |
| | **subtotal** | | **6** | **5** | **1** | **0** |
| **total** | | | **506** | **424** | **58** | **24** |

* Two of these three samples had been expired at the date of collection.

** Cinpharm recently became a Cameroonian company. However, three samples did not state the country of manufacture, therefore these three samples were listed in the category “not stated”.

*** The name of this manufacturer was given on different samples as "North China Pharmaceutical Co. Ltd.", or as "NCPC, PRC", or as "NCPC North Best". Since all of them appear to have the same contact address, they were considered in this study as a single manufacturer.

§ Falsified medicine; poor quality can not be attributed to the stated manufacturer.

Table S2: List of samples reported to fail GPHF Minilab TLC analysis, and of samples reported to pass GPHF Minilab TLC analysis but showing extreme deviations in USP assay testing, with their respective USP assay results

| Sample ID | API | USP assay classification | USP assay result [%] |
|---|---|---|---|
| **1) Samples reported to fail GPHF Minilab TLC analysis:** | | | |
| QMCA241 | Amoxicillin / clavulanic acid | extreme deviation | 0% / 0% |
| QMC266 | Metronidazole | extreme deviation | 0% |
| QMCA035 | Penicillin V | extreme deviation | 0% |
| QMCA001 | Salbutamol | extreme deviation | 54% |
| QMCA025 | Salbutamol | extreme deviation | 55% |
| QMCA215 | Salbutamol | extreme deviation | 61% |
| QMCA072 | Salbutamol | deviation | 81% |
| QMCA074 | Sulfamethoxazole / Trimethoprim | deviation | 91% / 95% |
| QMCA019 | Sulfamethoxazole / Trimethoprim | complies | 99% / 98% |
| QMCA212 | Sulfamethoxazole / Trimethoprim | complies | 102% / 100% |
| QMCA082 | Sulfamethoxazole / Trimethoprim | complies | 103% / 100% |
| QMCA032 | Amoxicillin | complies | 92% |
| QMCA210 | Penicillin V | complies | 93% |
| QMCA084 | Salbutamol | complies | 94% |
| QMCA184 | Ciprofloxacin | complies | 95% |
| **2) Samples reported to pass GPHF Minilab TLC analysis but showing extreme deviations in USP assay testing:** | | | |
| QMCA253 | Penicillin V | extreme deviation | 58% |
| QMCA107 | Penicillin V | extreme deviation | 68% |
| QMCA244 | Penicillin V | extreme deviation | 73% |
| QMCA177 | Penicillin V | extreme deviation | 76% |
| QMCA168 | Salbutamol | extreme deviation | 78% |
| QMCA179 | Salbutamol | extreme deviation | 78% |
| QMCA191 | Salbutamol | extreme deviation | 78% |
| QMCA239 | Salbutamol | extreme deviation | 79% |

## Table S3: Compendial quality results for the different products and batches as stated on the packaging

See separate pdf file
