# Supplementary material for: Substandard and Falsified Antibiotics and Medicines against Noncommunicable Diseases in Western Cameroon and Northeastern Democratic Republic of Congo
Source: Am J Trop Med Hyg. 2020 May 11;103(2):894–908. doi: 10.4269/ajtmh.20-0184 (PMC7410427; doi:10.4269/ajtmh.20-0184)
Supplement: Supplementary file 2 [file tpmd200184.SD2.pdf]

Table S3: List of all batches and brands investigated in this study, with their stated manufacturers and analytical results for assay and dissolution

Note: Medicines were collected in health facilities, i.e. at the point of care, and it is unknown whether the manufacturers' storage recommendation have been complied with from the time of manufacture until the time of sample collection. Changes in medicines quality may have occurred due to inappropriate transport and storage conditions, and non-compliance with USP specifications is therefore not necessarily due to substandard manufacturing or packaging. However, the quality results listed below reflect what patients receive in the investigated health facilities.

* Two of these three samples had been expired at the date of collection.

** Cinpharm recently became a Cameroonian company. However, three samples did not state the country of manufacture, therefore these three samples were listed in the category "not stated".

*** The name of this manufacturer was given on different samples as "North China Pharmaceutical Co. Ltd.", or as "NCPC, PRC", or as "NCPC North Best". Since all of them appear to have the same contact address, they were considered in this study as a single manufacturer.

[§] Falsified medicine; poor quality can not be attributed to the stated manufacturer.

							**USP assay**			**USP dissolution**			**USP assay and dissolution combined**		
**Stated continent of origin**	**Stated country of origin**	**Stated manufacturer**	**INN**	**Stated product name**	**Batchnumber**	**N**	**complies**	**deviation**	**extreme deviation**	**complies 2**	**deviation2**	**extreme deviation 2**	**complies 3**	**deviation 3**	**extreme deviation 3**
Africa	Benin	Pharmaquick	Furosemide	Furosemide Pharmaquick	991300	1	1	0	0	1	0	0	1	0	0
Africa	Benin	Pharmaquick	Glibenclamide	Glibenclamid Pharmaquick	965500	2	2	0	0	0	0	2	0	0	2
Africa	Benin	Pharmaquick	Hydrochlorothiazide	Hydrochlorothiazide Pharmaquick	992701	2	2	0	0	2	0	0	2	0	0
Africa	Benin	Pharmaquick	Hydrochlorothiazide	Hydrochlorothiazide Pharmaquick	992801	2	2	0	0	2	0	0	2	0	0
Africa	Burundi	Société industrielle Pharmaceutique (SIPHAR)	Doxycycline	Siphadox 100	SDC-001	1	1	0	0	1	0	0	1	0	0
Africa	Cameroon	Africure Pharmaceuticals Cameroon S.A.	Doxycycline	Doxycycline Capsules BP	4517001	2	2	0	0	1	1	0	1	1	0
Africa	Cameroon	Cinpharm **	Amoxicillin	Cinamox	18026	1	1	0	0	0	1	0	0	1	0
Africa	Cameroon	Cinpharm **	Sulfa/Trimet	Cincotrim	16001	1	1	0	0	1	0	0	1	0	0
Africa	Cameroon	Cinpharm **	Ciprofloxacin	Proloxcin	16001	1	1	0	0	1	0	0	1	0	0
Africa	DRC	Phatkin B.P.	Amoxicillin	Amoxin 250	02-16	1	1	0	0	1	0	0	1	0	0
Africa	DRC	Phatkin B.P.	Amoxicillin	Amoxin 500	09-16	1	1	0	0	0	1	0	0	1	0
Africa	DRC	Phatkin B.P.	Ciprofloxacin	Ciprokin-500	03-17	1	1	0	0	1	0	0	1	0	0
Africa	DRC	Phatkin B.P.	Ciprofloxacin	Ciprokin-500	17-17	1	0	1	0	1	0	0	0	1	0
Africa	DRC	Phatkin B.P.	Penicillin V	Peni-V	04-17	1	0	1	0	0	1	0	0	1	0
Africa	DRC	Zenufa Laboratoire	Ciprofloxacin	Ciproz 500	16T-141	1	0	1	0	1	0	0	0	1	0
Africa	DRC	Zenufa Laboratoire	Furosemide	Zenamide	14T-35	1	1	0	0	1	0	0	1	0	0
Africa	DRC	Zenufa Laboratoire	Furosemide	Zenamide	15T-75	1	1	0	0	1	0	0	1	0	0
Africa	DRC	Zenufa Laboratoire	Metronidazole	Zenogyl 250	16T-98	1	1	0	0	1	0	0	1	0	0
Africa	Ghana	Entrance Pharmaceuticals & Research Centre	Sulfa/Trimet	Co-Trimoxazole	NT17127	1	0	1	0	0	1	0	0	1	0
Africa	Ghana	Entrance Pharmaceuticals & Research Centre	Sulfa/Trimet	Co-Trimoxazole	NT17208	1	1	0	0	0	1	0	0	1	0
Africa	Ghana	Entrance Pharmaceuticals & Research Centre	Glibenclamide	Glibenclamide	NT17149	1	1	0	0	1	0	0	1	0	0
Africa	Ghana	Entrance Pharmaceuticals & Research Centre	Metronidazole	Metronidazole	NT17122	1	1	0	0	1	0	0	1	0	0
Africa	Ghana	Entrance Pharmaceuticals & Research Centre	Metronidazole	Metronidazole	NT17145	1	1	0	0	1	0	0	1	0	0
Africa	Kenya	Cosmos Limited	Sulfa/Trimet	Cosatrim	60446	1	1	0	0	1	0	0	1	0	0
Africa	Kenya	DAWA Limited	Metronidazole	Eflaron 250	1607130	2	2	0	0	2	0	0	2	0	0
Africa	Kenya	DAWA Limited	Furosemide	Frusemide	1605057	1	0	1	0	0	1	0	0	1	0
Africa	Kenya	DAWA Limited	Amoxicillin	Moxacil-250	1707321	1	1	0	0	1	0	0	1	0	0
Africa	Kenya	DAWA Limited	Amoxicillin	Moxacil-500	1706107	1	1	0	0	1	0	0	1	0	0

							USP assay			USP dissolution			USP assay and dissolution combined		
Stated continent of origin	Stated country of origin	Stated manufacturer	INN	Stated product name	Batchnumber	N	complies	deviation	extreme deviation	complies 2	deviation2	extreme deviation 2	complies 3	deviation 3	extreme deviation 3
Africa	Kenya	DAWA Limited	Salbutamol	Sabulin	1504062	1	1	0	0	1	0	0	1	0	0
Africa	Kenya	DAWA Limited	Salbutamol	Sabulin	1608101	1	1	0	0	1	0	0	1	0	0
Africa	Kenya	Elys Chemical Industries Ltd.	Sulfa/Trimet	CO-TRI	4E46	1	1	0	0	1	0	0	1	0	0
Africa	Kenya	Elys Chemical Industries Ltd.	Furosemide	Frusemide	4G68	1	1	0	0	1	0	0	1	0	0
Africa	Kenya	Elys Chemical Industries Ltd.	Furosemide	Frusemide	5H102	1	1	0	0	0	1	0	0	1	0
Africa	Kenya	Laboratory & Allied Ltd.	Amoxicillin	Kemoxyl 250	66735	1	1	0	0	1	0	0	1	0	0
Africa	Kenya	Laboratory & Allied Ltd.	Sulfa/Trimet	Lecotrim	67056	1	1	0	0	1	0	0	1	0	0
Africa	Kenya	MAC'S Pharmaceuticals Ltd.	Metronidazole	Metronyl	K2343	1	1	0	0	1	0	0	1	0	0
Africa	Kenya	MAC'S Pharmaceuticals Ltd.	Metronidazole	Metronyl	L3028	1	0	0	1 [§]	0	0	1 [§]	0	0	1 [§]
Africa	Kenya	Pharmaceutical Manufacturing Co. Ltd.	Salbutamol	Astalin	15-02040	1	0	1	0	0	1	0	0	1	0
Africa	Kenya	Regal Pharmaceuticals Ltd.	Penicillin V	Unipen	151876	1	1	0	0	1	0	0	1	0	0
Africa	Kenya	Regal Pharmaceuticals Ltd.	Penicillin V	Unipen 250	170093	1	1	0	0	1	0	0	1	0	0
Africa	Kenya	Regal Pharmaceuticals Ltd.	Penicillin V	Unipen 250	170888	1	1	0	0	1	0	0	1	0	0
Africa	Kenya	Regal Pharmaceuticals Ltd.	Penicillin V	Unipen 250	170890	1	1	0	0	1	0	0	1	0	0
Africa	Kenya	Regal Pharmaceuticals Ltd.	Sulfa/Trimet	Unitrim	160732	1	1	0	0	1	0	0	1	0	0
Africa	Nigeria	New Divine Favour Pharmaceutical Industries Ltd.	Doxycycline	New Divine Doxycycline Capsules	17	1	1	0	0	1	0	0	1	0	0
Africa	Senegal	Wintrop Pharma Sénégal Group SANOFI	Metronidazole	Flagyl 500	9705	1	1	0	0	1	0	0	1	0	0
Africa	Togo	Sprukfield	Sulfa/Trimet	Co-Trimoxazole	AT15001	2	2	0	0	2	0	0	2	0	0
Africa	Togo	Sprukfield	Sulfa/Trimet	Co-Trimoxazole	AT15007	1	1	0	0	1	0	0	1	0	0
Africa	Togo	Sprukfield	Sulfa/Trimet	Co-Trimoxazole	I3617	1	1	0	0	1	0	0	1	0	0
Africa	Uganda	Kampala Pharmaceutical Industries	Doxycycline	Azudox	2517	1	1	0	0	1	0	0	1	0	0
Africa	Uganda	Kampala Pharmaceutical Industries	Amoxicillin	Kam Amoxy Capsules	2417	1	1	0	0	1	0	0	1	0	0
Africa	Uganda	Kampala Pharmaceutical Industries	Sulfa/Trimet	Kam Cotri	1816	1	1	0	0	1	0	0	1	0	0
Africa	Uganda	Kampala Pharmaceutical Industries	Salbutamol	Kam Vent	0217	1	1	0	0	1	0	0	1	0	0
Africa	Uganda	Kampala Pharmaceutical Industries	Salbutamol	Kam Vent	0417	1	1	0	0	1	0	0	1	0	0
Africa	Uganda	Kampala Pharmaceutical Industries	Salbutamol	Kam Vent	0617	1	1	0	0	1	0	0	1	0	0
Africa	Uganda	Kampala Pharmaceutical Industries	Salbutamol	Kam Vent	0716	1	1	0	0	1	0	0	1	0	0
Africa	Uganda	Rene Industries Ltd.	Doxycycline	Doxyren	00217	1	1	0	0	1	0	0	1	0	0
Africa	Uganda	Rene Industries Ltd.	Sulfa/Trimet	Renetrim	04617	1	1	0	0	1	0	0	1	0	0
Americas	British West Indies	Prost Pharma (France)	Amoxicillin	Amoxdels-500	160952	1	1	0	0	1	0	0	1	0	0
Americas	British West Indies	Prost Pharma (France)	Sulfa/Trimet	Cotrimo-480mg	170610	1	1	0	0	1	0	0	1	0	0
Americas	USA	Sandoz	Furosemide	Furosemide	FT4986	1	1	0	0	1	0	0	1	0	0

							USP assay			USP dissolution			USP assay and dissolution combined		
Stated continent of origin	Stated country of origin	Stated manufacturer	INN	Stated product name	Batchnumber	N	complies	deviation	extreme deviation	complies 2	deviation2	extreme deviation 2	complies 3	deviation 3	extreme deviation 3
Asia	China	Anhui Chengshi Pharmaceutical Co. Ltd	Metronidazole	Metronidazole Tablets	170627	1	1	0	0	1	0	0	1	0	0
Asia	China	Anhui Medipharm Co. Ltd.	Ciprofloxacin	Cipro 500	1704581	1	0	1	0	1	0	0	0	1	0
Asia	China	Chifeng Wanze Pharmaceutical Co. Ltd.	Metronidazole	Metazol	X6021	1	1	0	0	1	0	0	1	0	0
Asia	China	CSPC Ouyi Pharmaceutical Co. Ltd.	Ciprofloxacin	Ciprofloxacine Tablets USP 500 mg	527160901	1	1	0	0	1	0	0	1	0	0
Asia	China	CSPC Ouyi Pharmaceutical Co. Ltd.	Ciprofloxacin	Ciprofloxacine Tablets USP 500 mg	527170206	1	1	0	0	0	1	0	0	1	0
Asia	China	CSPC Ouyi Pharmaceutical Co. Ltd.	Ciprofloxacin	Ciprofloxacine Tablets USP 500 mg	527170207	1	1	0	0	1	0	0	1	0	0
Asia	China	CSPC Ouyi Pharmaceutical Co. Ltd.	Ciprofloxacin	Ciprofloxacine Tablets USP 500 mg	784150901	1	1	0	0	1	0	0	1	0	0
Asia	China	CSPC Ouyi Pharmaceutical Co. Ltd.	Ciprofloxacin	Ciprofloxacine Tablets USP 500 mg	784150902	1	1	0	0	1	0	0	1	0	0
Asia	China	CSPC Ouyi Pharmaceutical Co. Ltd.	Ciprofloxacin	Ciprofloxacine Tablets USP 500 mg	784150904	1	1	0	0	1	0	0	1	0	0
Asia	China	CSPC Ouyi Pharmaceutical Co. Ltd.	Ciprofloxacin	Ciprofloxacine Tablets USP 500 mg	784160201	1	1	0	0	1	0	0	1	0	0
Asia	China	CSPC Ouyi Pharmaceutical Co. Ltd.	Ciprofloxacin	Ciprofloxacine Tablets USP 500 mg	784160501	1	1	0	0	1	0	0	1	0	0
Asia	China	CSPC Ouyi Pharmaceutical Co. Ltd.	Ciprofloxacin	Ciprofloxacine Tablets USP 500 mg	784161002	1	1	0	0	1	0	0	1	0	0
Asia	China	CSPC Ouyi Pharmaceutical Co. Ltd.	Sulfa/Trimet	Cotrimoxazole Tablets B.P	541141102	1	1	0	0	1	0	0	1	0	0
Asia	China	CSPC Ouyi Pharmaceutical Co. Ltd.	Sulfa/Trimet	Cotrimoxazole Tablets B.P	541150603	1	1	0	0	1	0	0	1	0	0
Asia	China	CSPC Ouyi Pharmaceutical Co. Ltd.	Sulfa/Trimet	Cotrimoxazole Tablets B.P	541161201	2	2	0	0	2	0	0	2	0	0
Asia	China	CSPC Ouyi Pharmaceutical Co. Ltd.	Doxycycline	Doxycycline Hyclate tablets USP	503150911	1	1	0	0	1	0	0	1	0	0
Asia	China	CSPC Ouyi Pharmaceutical Co. Ltd.	Doxycycline	Doxycycline Hyclate tablets USP	6140911	1	1	0	0	1	0	0	1	0	0
Asia	China	CSPC Ouyi Pharmaceutical Co. Ltd.	Metronidazole	Metronidazole 250 mg tables BP	825151102	1	1	0	0	1	0	0	1	0	0
Asia	China	CSPC Ouyi Pharmaceutical Co. Ltd.	Metronidazole	Metronidazole 250 mg tables BP	825160701	1	1	0	0	1	0	0	1	0	0
Asia	China	CSPC Ouyi Pharmaceutical Co. Ltd.	Metronidazole	Metronidazole 250 mg tables BP	825160702	1	1	0	0	1	0	0	1	0	0
Asia	China	CSPC Ouyi Pharmaceutical Co. Ltd.	Metronidazole	Metronidazole 250 mg tables BP	82516202	1	1	0	0	1	0	0	1	0	0
Asia	China	CSPC Ouyi Pharmaceutical Co. Ltd.	Metronidazole	Metronidazole 250 mg tables BP	825170302	1	1	0	0	1	0	0	1	0	0
Asia	China	CSPC Ouyi Pharmaceutical Co. Ltd.	Metronidazole	Metronidazole 250 mg tables BP	825170306	1	1	0	0	1	0	0	1	0	0

							USP assay			USP dissolution			USP assay and dissolution combined		
Stated continent of origin	Stated country of origin	Stated manufacturer	INN	Stated product name	Batchnumber	N	complies	deviation	extreme deviation	complies 2	deviation2	extreme deviation 2	complies 3	deviation 3	extreme deviation 3
Asia	China	CSPC Ouyi Pharmaceutical Co. Ltd.	Doxycycline	Unidoxy	160820	1	1	0	0	1	0	0	1	0	0
Asia	China	CSPC Zhongnuo Pharmaceuticals Co. Ltd.	Amoxicillin	Amoxicillin Capsules BP	706170381	1	1	0	0	1	0	0	1	0	0
Asia	China	CSPC Zhongnuo Pharmaceuticals Co. Ltd.	Amoxicillin	Amoxicillin Tablets USP	677150802	1	1	0	0	1	0	0	1	0	0
Asia	China	CSPC Zhongnuo Pharmaceuticals Co. Ltd.	Amoxicillin	Amoxicillin Tablets USP	678150103	1	1	0	0	1	0	0	1	0	0
Asia	China	CSPC Zhongnuo Pharmaceuticals Co. Ltd.	Amoxicillin	Amoxicillin Tablets for Oral Suspension	797160904	1	1	0	0	1	0	0	1	0	0
Asia	China	CSPC Zhongnuo Pharmaceuticals Co. Ltd.	Amoxicillin	Amoxicillin Tablets for Oral Suspension	797160908	2	2	0	0	2	0	0	2	0	0
Asia	China	CSPC Zhongnuo Pharmaceuticals Co. Ltd.	Amoxicillin	Amoxy-500	B6011	1	1	0	0	1	0	0	1	0	0
Asia	China	CSPC Zhongnuo Pharmaceuticals Co. Ltd.	Amoxicillin	Amoxy-500	B6012	1	1	0	0	1	0	0	1	0	0
Asia	China	CSPC Zhongnuo Pharmaceuticals Co. Ltd.	Penicillin V	Phenoxymethylpenicillin Tablets BP	688151109	2	2	0	0	2	0	0	2	0	0
Asia	China	Farmasino Pharmaceutical Co. Ltd	Amoxicillin	Amoxyn-500	160777	1	1	0	0	0	1	0	0	1	0
Asia	China	Farmasino Pharmaceutical Co. Ltd	Doxycycline	Doxiciclina	W160507	3	3	0	0	3	0	0	3	0	0
Asia	China	Farmasino Pharmaceutical Co. Ltd	Metronidazole	Mefagyl	SU20110076	1	1	0	0	1	0	0	1	0	0
Asia	China	Farmasino Pharmaceutical Co. Ltd	Penicillin V	Peni-V	W160939	1	1	0	0	1	0	0	1	0	0
Asia	China	Greenfield Pharmaceuticals (Jiang Su) Co. Ltd.	Ciprofloxacin	Cipromax Fort 500	173121091	1	1	0	0	1	0	0	1	0	0
Asia	China	Guilin Pharmaceutical Co. Ltd.	Sulfa/Trimet	Co-Trimoxazole USP	XN150764	1	1	0	0	1	0	0	1	0	0
Asia	China	Guilin Pharmaceutical Co. Ltd.	Sulfa/Trimet	Co-Trimoxazole USP	XN150766	1	1	0	0	1	0	0	1	0	0
Asia	China	Guilin Pharmaceutical Co. Ltd.	Sulfa/Trimet	Sulfamethoxazole and trimethoprim	XN150932	1	1	0	0	1	0	0	1	0	0
Asia	China	Jiangsu Pengyao Pharmaceutical Co. Ltd.	Ciprofloxacin	Ciprofloxacine Tablets USP	1510241	1	1	0	0	1	0	0	1	0	0
Asia	China	Jiangsu Pengyao Pharmaceutical Co. Ltd.	Metronidazole	Metronidazole Tablets BP	1608262	1	1	0	0	1	0	0	1	0	0
Asia	China	Jiangsu Ruinian Qianjin Pharm. Co.Ltd	Doxycycline	Doxycycline Sprukfield	141110	2	2	0	0	2	0	0	2	0	0
Asia	China	Jiangsu Ruinian Qianjin Pharm. Co.Ltd	Ciprofloxacin	Zeprox-500	170116	2	2	0	0	2	0	0	2	0	0
Asia	China	Jiangxi Xier Kangtai Pharmaceutical Co. Ltd.	Amoxicillin	Amoxycillin Capsules	150866	1	1	0	0	1	0	0	1	0	0
Asia	China	Jiangxi Xier Kangtai Pharmaceutical Co. Ltd.	Glibenclamide	Deominal	170303	2	2	0	0	0	2	0	0	2	0

							USP assay			USP dissolution			USP assay and dissolution combined		
Stated continent of origin	Stated country of origin	Stated manufacturer	INN	Stated product name	Batchnumber	N	complies	deviation	extreme deviation	complies 2	deviation2	extreme deviation 2	complies 3	deviation 3	extreme deviation 3
Asia	China	Jiangxi Xier Kangtai Pharmaceutical Co. Ltd.	Doxycycline	Surelife Doxycyline	161109	2	2	0	0	2	0	0	2	0	0
Asia	China	Jinzhou Jiuyang Pharmaceutical Co. Ltd	Metronidazole	Metronidazole Tablets	T20160801	2	2	0	0	2	0	0	2	0	0
Asia	China	Jinzhou Jiuyang Pharmaceutical Co. Ltd	Metronidazole	Metronidazole Tablets B.P. 250mg	T21	1	1	0	0	0	0	1	0	0	1
Asia	China	JSPY Pharmaceutical Co. Ltd.	Ciprofloxacin	Ciproin - 750	160422	1	1	0	0	1	0	0	1	0	0
Asia	China	JSPY Pharmaceutical Co. Ltd.	Ciprofloxacin	Ciproinh - 500	160713	1	1	0	0	1	0	0	1	0	0
Asia	China	JSPY Pharmaceutical Co. Ltd.	Metronidazole	Metrole-500	150205	1	1	0	0	1	0	0	1	0	0
Asia	China	Nanjing Baijingyu Pharmaceutical Co. Ltd.	Doxycycline	Doxycycline Hyclate Tablets USP	DHA15007	1	1	0	0	1	0	0	1	0	0
Asia	China	Nanjing Baijingyu Pharmaceutical Co. Ltd.	Doxycycline	Doxycycline Hyclate Tablets USP	DHA17001	1	1	0	0	1	0	0	1	0	0
Asia	China	Nanjing Baijingyu Pharmaceutical Co. Ltd.	Sulfa/Trimet	Sulfamethoxazole and Trimethoprim Tablets USP	TSH15051	1	1	0	0	1	0	0	1	0	0
Asia	China	Nanjing Sino Pharmaceutical Ltd.	Amoxicillin	Amoxicillin	160103	1	1	0	0	1	0	0	1	0	0
Asia	China	Ningbo Shuangwei Pharmaceutical Co. Ltd	Amoxicillin	Amoxzem	161025	1	1	0	0	1	0	0	1	0	0
Asia	China	Ningbo Shuangwei Pharmaceutical Co. Ltd	Amoxicillin	Amoxzem	161212	1	1	0	0	1	0	0	1	0	0
Asia	China	Ningbo Shuangwei Pharmaceutical Co. Ltd	Amoxicillin	Amoxzem Tab.	151121	2	2	0	0	2	0	0	2	0	0
Asia	China	Ningbo Shuangwei Pharmaceutical Co. Ltd	Amoxicillin	Amoxzem Tab.	170617	1	1	0	0	1	0	0	1	0	0
Asia	China	Ningbo Shuangwei Pharmaceutical Co. Ltd	Metronidazole	Metrozem-500	171034	1	1	0	0	1	0	0	1	0	0
Asia	China	North China Pharmaceutical Co. Ltd.***	Penicillin V	Phenoxymethylpencillin 250mg BP	150923	1	1	0	0	1	0	0	1	0	0
Asia	China	North China Pharmaceutical Co. Ltd.***	Penicillin V	Phenoxymethylpenicillin Tablets 250mg	160334	2	2	0	0	2	0	0	2	0	0
Asia	China	North China Pharmaceutical Co. Ltd.***	Penicillin V	Phenoxymethylpenicillin Tablets BP	160405	1	1	0	0	1	0	0	1	0	0
Asia	China	North China Pharmaceutical Co. Ltd.***	Penicillin V	Phenoxymethylpenicillin Tablets BP	160906	1	1	0	0	1	0	0	1	0	0
Asia	China	North China Pharmaceutical Co. Ltd.***	Penicillin V	Phenoxymethylpenicillin Tablets BP	160907	2	2	0	0	2	0	0	2	0	0
Asia	China	North China Pharmaceutical Co. Ltd.***	Penicillin V	Phenoxymethylpenicilline	C6007	1	1	0	0	1	0	0	1	0	0
Asia	China	Reyoung Pharmaceutical Co. Ltd.	Amoxicillin	Amoxiciline Ubigen	163131260	1	1	0	0	1	0	0	1	0	0
Asia	China	Reyoung Pharmaceutical Co. Ltd.	Amoxicillin	Amoxiciline Ubigen	173132202	1	1	0	0	1	0	0	1	0	0
Asia	China	Reyoung Pharmaceutical Co. Ltd.	Amoxicillin	Amoxicillin Capsules BP	153132033	1	1	0	0	1	0	0	1	0	0

							USP assay			USP dissolution			USP assay and dissolution combined		
Stated continent of origin	Stated country of origin	Stated manufacturer	INN	Stated product name	Batchnumber	N	complies	deviation	extreme deviation	complies 2	deviation2	extreme deviation 2	complies 3	deviation 3	extreme deviation 3
Asia	China	Reyoung Pharmaceutical Co. Ltd.	Amoxicillin	Amoxicillin Capsules BP	163132143	1	1	0	0	1	0	0	1	0	0
Asia	China	Reyoung Pharmaceutical Co. Ltd.	Amoxicillin	Amoxyn-500	160863	1	1	0	0	1	0	0	1	0	0
Asia	China	Reyoung Pharmaceutical Co. Ltd.	Amoxicillin	Amoxyn-500	P6064	1	1	0	0	1	0	0	1	0	0
Asia	China	Reyoung Pharmaceutical Co. Ltd.	Ciprofloxacin	Ciprofloxacin Tablets	163121042	1	1	0	0	1	0	0	1	0	0
Asia	China	Reyoung Pharmaceutical Co. Ltd.	Ciprofloxacin	Ciprofloxacin Tablets	163121044	2	2	0	0	2	0	0	2	0	0
Asia	China	Shandong Shenglu Pharmaceutical Co. Ltd	Penicillin V	Penicillin V	20160919	3	0	0	3	0	3	0	0	0	3
Asia	China	Shandong Shenglu Pharmaceutical Co. Ltd	Penicillin V	Transglobe Pen Tabs	170310	1	0	0	1	0	1	0	0	0	1
Asia	China	Shandong Xier Kangtai Pharm Co. Ltd	Metformin	Jeo-Phage Tablets	1610110	1	1	0	0	1	0	0	1	0	0
Asia	China	Shandong Yikang Pharmaceutical Co. Ltd.	Penicillin V	Penicillin V Potassium 250mg	170322	2	1	1	0	2	0	0	1	1	0
Asia	China	Shanghai Juchen Import and Exports Co. Ltd.	Amoxicillin	Konmoxy Capsules	173131530	1	1	0	0	1	0	0	1	0	0
Asia	China	Shanghai Juchen Import and Exports Co. Ltd.	Amoxicillin	Konmoxy Capsules	173131532	1	1	0	0	1	0	0	1	0	0
Asia	China	Shanghai Juchen Import and Exports Co. Ltd.	Amoxicillin	Konmoxy Capsules	173131533	1	1	0	0	1	0	0	1	0	0
Asia	China	Shanghai Juchen Import and Exports Co. Ltd.	Metronidazole	Metronidazole Tablets	171287	1	1	0	0	1	0	0	1	0	0
Asia	China	Shanxi Lianbang Pharmaceutical Co. Ltd.	Doxycycline	Doxycycline Capsules	160565	2	2	0	0	2	0	0	2	0	0
Asia	China	Sinochem Jiangsu Co. Ltd	Amoxicillin	Amoxicillin 500mg	170710	1	1	0	0	0	1	0	0	1	0
Asia	China	Sinochem Jiangsu Co. Ltd	Amoxicillin	Amoxicillin Capsules B.P	161011	2	2	0	0	2	0	0	2	0	0
Asia	China	Sinochem Jiangsu Co. Ltd	Ciprofloxacin	Ciprofloxacine Tablets USP	160283	1	1	0	0	1	0	0	1	0	0
Asia	China	Sinochem Jiangsu Co. Ltd	Ciprofloxacin	Ciprofloxacine Tablets USP	1605603	4	3	1	0	4	0	0	3	1	0
Asia	China	Sinochem Jiangsu Co. Ltd	Ciprofloxacin	Ciprolif-500	170751	2	1	1	0	2	0	0	1	1	0
Asia	China	Sinochem Jiangsu Co. Ltd	Metronidazole	Metronidazole GP	170717	1	1	0	0	0	0	1	0	0	1
Asia	China	Sinochem Jiangsu Co. Ltd	Metronidazole	Metronidazole GP	171201	2	2	0	0	0	0	2	0	0	2
Asia	China	Sinochem Jiangsu Co. Ltd	Penicillin V	Peni-V	170504	1	1	0	0	1	0	0	1	0	0
Asia	China	Sishui xier Kang Pharmaceutical Co.Ltd	Penicillin V	Penicillin V Potassium - 5000,000	160718	1	0	0	1	0	1	0	0	0	1
Asia	China	Yanzhou Xierkangtai pharmaceutical Co. Ltd.	Amoxicillin	Amoxicillin	S37	1	1	0	0	1	0	0	1	0	0
Asia	China	Yanzhou Xierkangtai pharmaceutical Co. Ltd.	Doxycycline	Doxycycline	S06	1	1	0	0	1	0	0	1	0	0
Asia	China	Yanzhou Xierkangtai pharmaceutical Co. Ltd.	Penicillin V	Penicillin VK Tablets	S20170329	1	0	1	0	1	0	0	0	1	0
Asia	Hong Kong	Hongkong Prost Medicines And Health Products Co. Ltd.	Hydrochlorothiazide	Hydrochlorothiazide	160815	3	3	0	0	3	0	0	3	0	0

							USP assay			USP dissolution			USP assay and dissolution combined		
**Stated continent of origin**	**Stated country of origin**	**Stated manufacturer**	**INN**	**Stated product name**	**Batchnumber**	**N**	**complies**	**deviation**	**extreme deviation**	**complies 2**	**deviation2**	**extreme deviation 2**	**complies 3**	**deviation 3**	**extreme deviation 3**
Asia	India	Agog Pharma Ltd.	Doxycycline	Agodox	C55016	1	1	0	0	1	0	0	1	0	0
Asia	India	Agog Pharma Ltd.	Doxycycline	Agodox	C73018	1	1	0	0	1	0	0	1	0	0
Asia	India	Agog Pharma Ltd.	Sulfa/Trimet	Co-Trimoxazole Tablets BP Trimago	T64108	1	1	0	0	1	0	0	1	0	0
Asia	India	Agog Pharma Ltd.	Sulfa/Trimet	Co-Trimoxazole Tablets BP Trimago	T71155	1	1	0	0	1	0	0	1	0	0
Asia	India	Alkem Laboratories Ltd.	Amoxi/Clav	Acinet	6150096	1	1	0	0	0	1	0	0	1	0
Asia	India	Arco Pharma Pvt. Ltd	Furosemide	Frusema	562E	1	1	0	0	0	1	0	0	1	0
Asia	India	Arco Pharma Pvt. Ltd	Furosemide	Frusema	618E	3	3	0	0	1	2	0	1	2	0
Asia	India	Arco Pharma Pvt. Ltd	Furosemide	Frusema	619E	3	3	0	0	0	3	0	0	3	0
Asia	India	Asence Pharma Pvt. Ltd.	Furosemide	Furosemide Tabrad	T-799002	2	2	0	0	2	0	0	2	0	0
Asia	India	Asence Pharma Pvt. Ltd.	Metronidazole	Metronidazole 500	T-800003	1	1	0	0	1	0	0	1	0	0
Asia	India	Asence Pharma Pvt. Ltd.	Furosemide	Tafuros 40	AC25701	2	2	0	0	1	1	0	1	1	0
Asia	India	Asence Pharma Pvt. Ltd.	Amoxi/Clav	Tamclav 1G	PT7088	1	1	0	0	0	1	0	0	1	0
Asia	India	Astra Lifecare Pvt. Ltd.	Salbutamol	Asbutol-P4	023	1	1	0	0	1	0	0	1	0	0
Asia	India	Astra Lifecare Pvt. Ltd.	Doxycycline	Asdoxin	617	1	1	0	0	0	0	1	0	0	1
Asia	India	Astra Lifecare Pvt. Ltd.	Ciprofloxacin	Asflox-500	463	3	3	0	0	3	0	0	3	0	0
Asia	India	Astra Lifecare Pvt. Ltd.	Furosemide	Asix	028	1	1	0	0	1	0	0	1	0	0
Asia	India	Astra Lifecare Pvt. Ltd.	Furosemide	Asix	031	1	1	0	0	1	0	0	1	0	0
Asia	India	Astra Lifecare Pvt. Ltd.	Metronidazole	Astrogyl	497	1	1	0	0	1	0	0	1	0	0
Asia	India	Astra Lifecare Pvt. Ltd.	Penicillin V	As-V	185	1	1	0	0	1	0	0	1	0	0
Asia	India	Astra Lifecare Pvt. Ltd.	Penicillin V	As-V	187	1	1	0	0	1	0	0	1	0	0
Asia	India	Astra Lifecare Pvt. Ltd.	Atenolol	Hyperlok-100	025	1	1	0	0	0	0	1	0	0	1
Asia	India	Aura pharmaceuticals Pvt. Ltd	Sulfa/Trimet	Cotrimex-480	01	1	1	0	0	0	1	0	0	1	0
Asia	India	Aura pharmaceuticals Pvt. Ltd	Metronidazole	Megyl	006	2	2	0	0	2	0	0	2	0	0
Asia	India	Aura pharmaceuticals Pvt. Ltd	Metronidazole	Megyl	009	1	1	0	0	1	0	0	1	0	0
Asia	India	Aura pharmaceuticals Pvt. Ltd	Salbutamol	Salbutamol Tablets BP	001	5	2	3	0	5	0	0	2	3	0
Asia	India	Aurobindo Pharma Ltd.	Amoxi/Clav	Koact 625	EL5016026-D	1	1	0	0	0	1	0	0	1	0
Asia	India	Axon Drugs Pvt. Ltd.	Metformin	Asur-850	16ASU01	1	1	0	0	1	0	0	1	0	0
Asia	India	Bliss GVS Pharma Ltd.	Metformin	BGMET 850	BMT004	1	0	1	0	0	1	0	0	1	0
Asia	India	Cadila Healthcare Ltd.	Atenolol	Catenol 100	GR2742	1	1	0	0	1	0	0	1	0	0
Asia	India	Cipla Ltd.	Ciprofloxacin	Ciplox-500	ID55812	1	1	0	0	1	0	0	1	0	0
Asia	India	Ciron Drugs and Pharmaceuticals Ltd.	Metformin	Shalformin	5E01015	2	2	0	0	2	0	0	2	0	0
Asia	India	Combitic Global Caplet Pvt. Ltd.	Doxycycline	Doxynol 200	CDY-13	1	1	0	0	1	0	0	1	0	0
Asia	India	Fourrts	Sulfa/Trimet	Co-Trimoxazole Tablets BP Megatrim	C1796	1	1	0	0	1	0	0	1	0	0
Asia	India	Fourrts	Doxycycline	Doxycycline Hyclate Tablets USP	E1193	1	1	0	0	1	0	0	1	0	0
Asia	India	Fourrts	Metformin	METFIL	C0335	1	1	0	0	1	0	0	1	0	0

							USP assay			USP dissolution			USP assay and dissolution combined		
Stated continent of origin	Stated country of origin	Stated manufacturer	INN	Stated product name	Batchnumber	N	complies	deviation	extreme deviation	complies 2	deviation2	extreme deviation 2	complies 3	deviation 3	extreme deviation 3
Asia	India	Global Pharma Healthcare Pvt. Ltd.	Hydrochlorothiazide	Hydrochlorothiazide comprimes BP	TE399	4	4	0	0	4	0	0	4	0	0
Asia	India	Holden Medical Laboratories Pvt. Ltd.	Atenolol	Atenolol Tablets BP	HE15C28	1	1	0	0	1	0	0	1	0	0
Asia	India	Holden Medical Laboratories Pvt. Ltd.	Ciprofloxacin	Ciprofloxacin Tablets USP	HE16D39	1	1	0	0	1	0	0	1	0	0
Asia	India	Holden Medical Laboratories Pvt. Ltd.	Glibenclamide	Glibenclamide Tablets B.P	HE15L66	1	1	0	0	1	0	0	1	0	0
Asia	India	Intermed	Amoxi/Clav	Amoxicillin and Clavulanate Potassium Tablets	QTN02	1	1	0	0	1	0	0	1	0	0
Asia	India	Ipca Laboratories Ltd.	Amoxi/Clav	Rapiclav-1g	CIJ177040	1	1	0	0	1	0	0	1	0	0
Asia	India	J. B. Chemicals and Pharmaceuticals Ltd.	Metronidazole	Unique's Metrogyl 200	AM56004	1	1	0	0	1	0	0	1	0	0
Asia	India	Kopran Limited	Amoxicillin	AMYN-250	S3646054	1	1	0	0	1	0	0	1	0	0
Asia	India	Kopran Limited	Sulfa/Trimet	Trim - 480	K3806011	1	1	0	0	1	0	0	1	0	0
Asia	India	Leben Laboratories Pvt. Ltd	Doxycycline	Doxyleb	C137	1	1	0	0	1	0	0	1	0	0
Asia	India	Lincoln Pharmaceuticals Ltd.	Doxycycline	Alldox	AA5006	1	1	0	0	1	0	0	1	0	0
Asia	India	Lincoln Pharmaceuticals Ltd.	Doxycycline	Alldox	AA7001	1	1	0	0	1	0	0	1	0	0
Asia	India	Lincoln Pharmaceuticals Ltd.	Ciprofloxacin	CEEPRO-500	DY6028	1	1	0	0	1	0	0	1	0	0
Asia	India	Lincoln Pharmaceuticals Ltd.	Ciprofloxacin	Ciprofloxacine Ubigen	GK6007	1	1	0	0	1	0	0	1	0	0
Asia	India	Lincoln Pharmaceuticals Ltd.	Ciprofloxacin	Ciprofloxacine Ubigen	GK7010	1	1	0	0	1	0	0	1	0	0
Asia	India	Lincoln Pharmaceuticals Ltd.	Sulfa/Trimet	Cotrimoxazole Ubigen	GM6006	2	2	0	0	2	0	0	2	0	0
Asia	India	Lincoln Pharmaceuticals Ltd.	Sulfa/Trimet	Sulphatrim	NE6004	1	1	0	0	1	0	0	1	0	0
Asia	India	Lord Lifescience Pvt. Ltd.	Salbutamol	Salbesone	HONO	1	0	1	0	1	0	0	0	1	0
Asia	India	Macleods Pharmaceuticals Ltd.	Ciprofloxacin	Coflox-500	FCF657A	1	1	0	0	1	0	0	1	0	0
Asia	India	Macleods Pharmaceuticals Ltd.	Ciprofloxacin	Coflox-500	FCF659A	1	1	0	0	1	0	0	1	0	0
Asia	India	Macleods Pharmaceuticals Ltd.	Sulfa/Trimet	Co-trimoxazole Tablets BP 480mg	1708	1	1	0	0	1	0	0	1	0	0
Asia	India	Macleods Pharmaceuticals Ltd.	Sulfa/Trimet	Co-trimoxazole Tablets BP 480mg	HTF713A	1	1	0	0	1	0	0	1	0	0
Asia	India	Mancare pharmaceutical Ltd	Furosemide	Frunmide	TPF03	1	1	0	0	1	0	0	1	0	0
Asia	India	Mancare pharmaceutical Ltd	Furosemide	Frunmide	TRI18	1	1	0	0	1	0	0	1	0	0
Asia	India	Mancare pharmaceutical Ltd	Furosemide	Lancize	TRF28	1	1	0	0	1	0	0	1	0	0
Asia	India	Mancare pharmaceutical Ltd	Furosemide	Lancize	TRF32	1	1	0	0	1	0	0	1	0	0
Asia	India	Maneesh Pharmaceuticals Ltd	Doxycycline	Doxycycline Tablets	S01	1	1	0	0	1	0	0	1	0	0
Asia	India	Maxheal Laboratories Pvt. Ltd.	Salbutamol	Salbutamol	SW7003	1	0	1	0	0	1	0	0	1	0
Asia	India	Maxheal Laboratories Pvt. Ltd.	Ciprofloxacin	Wincip-500	WC6001	1	1	0	0	1	0	0	1	0	0
Asia	India	Maxtar Bio-Genics	Sulfa/Trimet	Cotrimoxazole Pextran_SS	MT4T-1601	2	2	0	0	0	2	0	0	2	0
Asia	India	Maxtar Bio-Genics	Metformin	Maxformin-500	MT3M-1602	1	1	0	0	1	0	0	1	0	0
Asia	India	Maxtar Bio-Genics	Metformin	Maxformin-500	MT3M-1607	1	1	0	0	1	0	0	1	0	0
Asia	India	Maxtar Bio-Genics	Metformin	Maxformin-500	MXTEJ1701	1	1	0	0	1	0	0	1	0	0

							USP assay			USP dissolution			USP assay and dissolution combined		
**Stated continent of origin**	**Stated country of origin**	**Stated manufacturer**	**INN**	**Stated product name**	**Batchnumber**	**N**	**complies**	**deviation**	**extreme deviation**	**complies 2**	**deviation2**	**extreme deviation 2**	**complies 3**	**deviation 3**	**extreme deviation 3**
Asia	India	Maxtar Bio-Genics	Metronidazole	Metzole-500	MT7T-1601	2	2	0	0	2	0	0	2	0	0
Asia	India	Maxtar Bio-Genics	Salbutamol	Salbutamol Comprimes BP	MTSA-1402	3*	0	0	3*	0	0	3*	0	0	3*
Asia	India	Maxtar Bio-Genics	Salbutamol	Salbutamol Comprimes BP	MTSA-1602	4	4	0	0	4	0	0	4	0	0
Asia	India	Medicamen Biotech Ltd.	Ciprofloxacin	Ciprofloxacine USP 500 mg	NT6698	1	1	0	0	1	0	0	1	0	0
Asia	India	Medicamen Biotech Ltd.	Doxycycline	Doxycycline Hyclate	NT7540	1	1	0	0	1	0	0	1	0	0
Asia	India	Medicamen Biotech Ltd.	Glibenclamide	Glibenclamide	NT5047	1	1	0	0	1	0	0	1	0	0
Asia	India	Medicamen Biotech Ltd.	Glibenclamide	Glibenclamide	NT5048	2	2	0	0	2	0	0	2	0	0
Asia	India	Medicamen Biotech Ltd.	Metformin	Metformin	NT5524	1	1	0	0	1	0	0	1	0	0
Asia	India	Medicamen Biotech Ltd.	Metformin	Metformin	NT5525	1	1	0	0	0	1	0	0	1	0
Asia	India	Medicamen Biotech Ltd.	Metronidazole	Metronidazole	NT5371	1	1	0	0	1	0	0	1	0	0
Asia	India	Medicef Pharma	Amoxi/Clav	Arauclav	ET16G014	1	1	0	0	1	0	0	1	0	0
Asia	India	Medicef Pharma	Amoxi/Clav	Moxyclav	ET16E008	1	1	0	0	1	0	0	1	0	0
Asia	India	Medicef Pharma	Amoxi/Clav	Moxyclav	ET16G010	1	1	0	0	1	0	0	1	0	0
Asia	India	Medicef Pharma	Amoxi/Clav	Moxyclav	ET16G020	1	1	0	0	1	0	0	1	0	0
Asia	India	Medico Remedies Pvt. Ltd.	Salbutamol	Salbutamol Tablets BP	SAU513	2	0	0	2	0	2	0	0	0	2
Asia	India	Medico Remedies Pvt. Ltd.	Salbutamol	Salbutamol Tablets BP	SAU537	1	0	0	1	1	0	0	0	0	1
Asia	India	Medico Remedies Pvt. Ltd.	Salbutamol	Salbutamol Tablets BP	SAU602	1	0	1	0	0	1	0	0	1	0
Asia	India	Medico Remedies Pvt. Ltd.	Salbutamol	Salbutamol Tablets BP	SAU630	1	0	0	1	1	0	0	0	0	1
Asia	India	Medley Pharmaceuticals Ltd.	Ciprofloxacin	Ecoflox-500	D60130	1	1	0	0	1	0	0	1	0	0
Asia	India	Medley Pharmaceuticals Ltd.	Ciprofloxacin	Ecoflox-500	D60184	1	1	0	0	1	0	0	1	0	0
Asia	India	Medley Pharmaceuticals Ltd.	Ciprofloxacin	Ecoflox-500	D60246	1	1	0	0	1	0	0	1	0	0
Asia	India	Medley Pharmaceuticals Ltd.	Ciprofloxacin	Ecoflox-500	D60263	1	1	0	0	1	0	0	1	0	0
Asia	India	Medley Pharmaceuticals Ltd.	Ciprofloxacin	Ecoflox-500	D60445	1	1	0	0	1	0	0	1	0	0
Asia	India	Medopharm Pvt. Ltd.	Amoxi/Clav	Amoxicillin 500mg + Clavulanic acid 125mg BP	F456733	1	1	0	0	1	0	0	1	0	0
Asia	India	Medopharm Pvt. Ltd.	Amoxicillin	Amoxicillin Gelules	1475017	1	1	0	0	1	0	0	1	0	0
Asia	India	Medopharm Pvt. Ltd.	Amoxicillin	Amoxicillin Tablets USP 250	15329002	1	1	0	0	1	0	0	1	0	0
Asia	India	Medopharm Pvt. Ltd.	Amoxicillin	Amoxicillin Tablets USP 500	16144002	1	1	0	0	1	0	0	1	0	0
Asia	India	Medopharm Pvt. Ltd.	Amoxicillin	Amoxicillin Tablets USP 500	16363002	1	1	0	0	1	0	0	1	0	0
Asia	India	Medopharm Pvt. Ltd.	Ciprofloxacin	Ciprofloxacine 500 mg USP	5E 101	1	1	0	0	1	0	0	1	0	0
Asia	India	Medopharm Pvt. Ltd.	Ciprofloxacin	Ciprofloxacine Comprimes USP	217090001	1	1	0	0	1	0	0	1	0	0
Asia	India	Medopharm Pvt. Ltd.	Ciprofloxacin	Ciprofloxacine Comprimes USP	6C66	1	1	0	0	1	0	0	1	0	0
Asia	India	Medopharm Pvt. Ltd.	Ciprofloxacin	Ciprofloxacine Comprimes USP	6C67	2	2	0	0	2	0	0	2	0	0
Asia	India	Medopharm Pvt. Ltd.	Amoxi/Clav	Clavumoccid	16213003	1	1	0	0	1	0	0	1	0	0
Asia	India	Medopharm Pvt. Ltd.	Amoxi/Clav	Cledomox 562.5	17361003	1	0	1	0	1	0	0	0	1	0
Asia	India	Medopharm Pvt. Ltd.	Amoxi/Clav	Co-amoxiclav	1680002	1	1	0	0	1	0	0	1	0	0
Asia	India	Medopharm Pvt. Ltd.	Sulfa/Trimet	Co-trimoxazole BP	4J32	1	1	0	0	1	0	0	1	0	0
Asia	India	Medopharm Pvt. Ltd.	Sulfa/Trimet	Co-trimoxazole BP	4J34	1	1	0	0	1	0	0	1	0	0
Asia	India	Medopharm Pvt. Ltd.	Sulfa/Trimet	Co-trimoxazole BP	4MB107	1	1	0	0	1	0	0	1	0	0
Asia	India	Medopharm Pvt. Ltd.	Sulfa/Trimet	Co-trimoxazole BP	6MD354	1	1	0	0	1	0	0	1	0	0
Asia	India	Medopharm Pvt. Ltd.	Sulfa/Trimet	Co-trimoxazole BP	6MD360	1	1	0	0	1	0	0	1	0	0
Asia	India	Medopharm Pvt. Ltd.	Sulfa/Trimet	Co-trimoxazole BP	6MD364	2	2	0	0	2	0	0	2	0	0
Asia	India	Medopharm Pvt. Ltd.	Sulfa/Trimet	Co-trimoxazole BP	6MG195	1	1	0	0	1	0	0	1	0	0

							USP assay			USP dissolution			USP assay and dissolution combined		
Stated continent of origin	Stated country of origin	Stated manufacturer	INN	Stated product name	Batchnumber	N	complies	deviation	extreme deviation	complies 2	deviation2	extreme deviation 2	complies 3	deviation 3	extreme deviation 3
Asia	India	Medopharm Pvt. Ltd.	Sulfa/Trimet	Co-trimoxazole BP	6MG198	1	1	0	0	1	0	0	1	0	0
Asia	India	Medopharm Pvt. Ltd.	Sulfa/Trimet	Co-trimoxazole USP	XN150772	1	1	0	0	1	0	0	1	0	0
Asia	India	Medopharm Pvt. Ltd.	Doxycycline	Doxycycline Hyclate USP	4MJ124	1	1	0	0	1	0	0	1	0	0
Asia	India	Medopharm Pvt. Ltd.	Doxycycline	Doxycycline Hyclate USP	5MH47	1	1	0	0	1	0	0	1	0	0
Asia	India	Medopharm Pvt. Ltd.	Doxycycline	Doxycycline Hyclate USP	5MJ146	2	2	0	0	2	0	0	2	0	0
Asia	India	Medopharm Pvt. Ltd.	Penicillin V	Fenoximetilpenicilina	1208524	1	1	0	0	1	0	0	1	0	0
Asia	India	Medopharm Pvt. Ltd.	Furosemide	Furosemid BP	4MJ129	1	1	0	0	1	0	0	1	0	0
Asia	India	Medopharm Pvt. Ltd.	Doxycycline	Generic Plus Doxycycline Hyclate 100mg USP	6MF123	2	2	0	0	2	0	0	2	0	0
Asia	India	Medopharm Pvt. Ltd.	Metformin	Metformin Tablets 500 mg BP	7MA42	1	1	0	0	1	0	0	1	0	0
Asia	India	Medopharm Pvt. Ltd.	Metronidazole	Metronidazole	4MJ164	1	1	0	0	1	0	0	1	0	0
Asia	India	Medopharm Pvt. Ltd.	Metronidazole	Metronidazole 250 mg BP	5B07	1	1	0	0	1	0	0	1	0	0
Asia	India	Medopharm Pvt. Ltd.	Metronidazole	Metronidazole 250 mg BP	5F42	1	1	0	0	0	1	0	0	1	0
Asia	India	Medopharm Pvt. Ltd.	Metronidazole	Metronidazole 250 mg BP	5MA91	2	2	0	0	2	0	0	2	0	0
Asia	India	Medopharm Pvt. Ltd.	Metronidazole	Metronidazole 250 mg BP	5ME187	1	1	0	0	1	0	0	1	0	0
Asia	India	Medopharm Pvt. Ltd.	Salbutamol	Salbutamol Tablets BP	6MF93	2	2	0	0	2	0	0	2	0	0
Asia	India	Medopharm Pvt. Ltd.	Salbutamol	Salbutamol Tablets BP	6MF94	2	2	0	0	2	0	0	2	0	0
Asia	India	Mepro Pharmaceuticals Pvt. Ltd.	Ciprofloxacin	Ciprofloxacin	UCP224	1	1	0	0	1	0	0	1	0	0
Asia	India	Mepro Pharmaceuticals Pvt. Ltd.	Doxycycline	Doxycycline	UDH220	1	1	0	0	1	0	0	1	0	0
Asia	India	Micro Labs Ltd.	Furosemide	Furosemide 40mg BP	FRIH0077	4	4	0	0	4	0	0	4	0	0
Asia	India	Milan Laboratories (India) Pvt. Ltd	Sulfa/Trimet	Co-Trimoxazole	MG16041	1	1	0	0	1	0	0	1	0	0
Asia	India	Milan Laboratories (India) Pvt. Ltd	Amoxicillin	Miloxy 250	MP17005	1	1	0	0	1	0	0	1	0	0
Asia	India	Milan Laboratories (India) Pvt. Ltd	Amoxicillin	Miloxy 250	MP17069	1	1	0	0	1	0	0	1	0	0
Asia	India	Milan Laboratories (India) Pvt. Ltd	Amoxicillin	Miloxy 250	MP17210	1	1	0	0	1	0	0	1	0	0
Asia	India	Milan Laboratories (India) Pvt. Ltd	Amoxicillin	Miloxy 250	MP17258	1	1	0	0	1	0	0	1	0	0
Asia	India	Milan Laboratories (India) Pvt. Ltd	Penicillin V	Penicillin-Tablets	MP0268	1	0	1	0	1	0	0	0	1	0
Asia	India	Nem Laboratories Pvt. Ltd.	Furosemide	Frusemide	FRS615	1	1	0	0	1	0	0	1	0	0
Asia	India	not stated	Amoxi/Clav	Oxynic	B1730	1	1	0	0	1	0	0	1	0	0
Asia	India	Osaka Pharmaceuticals Pvt. Ltd.	Glibenclamide	Transglobe glibenclamide	6A038	3	1	2	0	3	0	0	1	2	0
Asia	India	PIL Pharmaceuticals Pvt. Ltd.	Amoxi/Clav	Co-amoxiclav Tablets BP 625mg	AAGB6027	1	1	0	0	1	0	0	1	0	0
Asia	India	Prashi Pharma Pvt. Ltd	Furosemide	Frusemide	FR-01	2	2	0	0	0	1	1	0	1	1
Asia	India	Prashi Pharma Pvt. Ltd	Furosemide	Frusemide	FR-02	2	2	0	0	0	2	0	0	2	0
Asia	India	Prashi Pharma Pvt. Ltd	Furosemide	Frusemide	FR-03	1	1	0	0	0	1	0	0	1	0
Asia	India	Prashi Pharma Pvt. Ltd	Metronidazole	Metro 250	MT-133	1	1	0	0	1	0	0	1	0	0
Asia	India	Shalina Laboratories Pvt. Ltd.	Sulfa/Trimet	Sulfatrim	J7007	1	1	0	0	1	0	0	1	0	0
Asia	India	Sparsh Bio-Tech Pvt. Ltd.	Amoxicillin	HIPEN	HC225	1	1	0	0	1	0	0	1	0	0

							USP assay			USP dissolution			USP assay and dissolution combined		
Stated continent of origin	Stated country of origin	Stated manufacturer	INN	Stated product name	Batchnumber	N	complies	deviation	extreme deviation	complies 2	deviation2	extreme deviation 2	complies 3	deviation 3	extreme deviation 3
Asia	India	Sparsh Bio-Tech Pvt. Ltd.	Amoxicillin	HIPEN	HC232	1	1	0	0	1	0	0	1	0	0
Asia	India	Sparsh Bio-Tech Pvt. Ltd.	Penicillin V	Speniv Tablets 250	PT448	1	1	0	0	1	0	0	1	0	0
Asia	India	Sparsh Bio-Tech Pvt. Ltd.	Penicillin V	Speniv Tablets 250	PT457	1	1	0	0	1	0	0	1	0	0
Asia	India	Sparsh Bio-Tech Pvt. Ltd.	Penicillin V	Speniv Tablets 250	PT460	1	1	0	0	1	0	0	1	0	0
Asia	India	Sparsh Bio-Tech Pvt. Ltd.	Penicillin V	Speniv Tablets 250	PT467	2	2	0	0	2	0	0	2	0	0
Asia	India	Strides Arcolab Limited	Amoxicillin	Amoxicillin Tablets	AG-044	1	1	0	0	1	0	0	1	0	0
Asia	India	Strides Arcolab Limited	Amoxicillin	Amoxicillin Tablets	AG-064	1	1	0	0	1	0	0	1	0	0
Asia	India	Strides Arcolab Limited	Ciprofloxacin	Ciprofloxacine Tablets USP	7750797	1	1	0	0	1	0	0	1	0	0
Asia	India	Strides Arcolab Limited	Ciprofloxacin	Ciprofloxacine Tablets USP	7750816	1	1	0	0	1	0	0	1	0	0
Asia	India	Strides Arcolab Limited	Sulfa/Trimet	Co-trimoxazole Tablets BP	7750175	1	1	0	0	1	0	0	1	0	0
Asia	India	Strides Arcolab Limited	Sulfa/Trimet	Co-trimoxazole Tablets BP	7750676	1	1	0	0	1	0	0	1	0	0
Asia	India	Strides Arcolab Limited	Sulfa/Trimet	Co-trimoxazole Tablets BP	7750677	1	1	0	0	1	0	0	1	0	0
Asia	India	Strides Arcolab Limited	Sulfa/Trimet	Co-trimoxazole Tablets BP	7750714	1	1	0	0	1	0	0	1	0	0
Asia	India	Strides Arcolab Limited	Sulfa/Trimet	Co-trimoxazole Tablets BP	7750718	1	1	0	0	1	0	0	1	0	0
Asia	India	Strides Arcolab Limited	Sulfa/Trimet	Co-trimoxazole Tablets BP	7750719	1	1	0	0	1	0	0	1	0	0
Asia	India	Strides Arcolab Limited	Furosemide	Frusemide BP	7351588	2	1	1	0	1	1	0	0	2	0
Asia	India	Strides Arcolab Limited	Metformin	Metformin Tablets BP	7351219	1	1	0	0	1	0	0	1	0	0
Asia	India	Strides Arcolab Limited	Metformin	Metformin Tablets BP	7351823	1	1	0	0	1	0	0	1	0	0
Asia	India	Strides Arcolab Limited	Metformin	Metformin Tablets BP	7351824	1	1	0	0	1	0	0	1	0	0
Asia	India	Strides Arcolab Limited	Metronidazole	Metronidazole Comprimes BP	7750163	1	1	0	0	1	0	0	1	0	0
Asia	India	Strides Arcolab Limited	Metronidazole	Metronidazole Comprimes BP	7750581	2	2	0	0	2	0	0	2	0	0
Asia	India	Strides Arcolab Limited	Metronidazole	Metronidazole Comprimes BP	7750973	1	1	0	0	1	0	0	1	0	0
Asia	India	Strides Arcolab Limited	Metronidazole	Metronidazole Comprimes BP	7751013	1	1	0	0	1	0	0	1	0	0
Asia	India	Strides Arcolab Limited	Metronidazole	Metronidazole Comprimes BP	7751017	1	1	0	0	1	0	0	1	0	0
Asia	India	Strides Arcolab Limited	Metronidazole	Metronidazole Comprimes BP	7751018	1	1	0	0	1	0	0	1	0	0
Asia	India	Strides Arcolab Limited	Metronidazole	Metronidazole Comprimes BP	7751038	1	1	0	0	1	0	0	1	0	0
Asia	India	Strides Shasun Limited	Ciprofloxacin	Ciprofloxacine	7352249	1	1	0	0	1	0	0	1	0	0
Asia	India	Strides Shasun Limited	Doxycycline	Doxycycline Gelules BP	7750636	4	4	0	0	4	0	0	4	0	0
Asia	India	Strides Shasun Limited	Metformin	Metformin	7352132	2	2	0	0	2	0	0	2	0	0
Asia	India	Strides Shasun Limited	Metronidazole	Metrosim-200	7351898	1	0	1	0	1	0	0	0	1	0
Asia	India	Strides Shasun Limited	Metronidazole	Metrosim-200	7352023	1	1	0	0	1	0	0	1	0	0
Asia	India	Strides Shasun Limited	Metronidazole	Metrosim-200	7352053	1	1	0	0	1	0	0	1	0	0
Asia	India	Strides Shasun Limited	Metronidazole	Metrosim-200	7352173	1	0	1	0	1	0	0	0	1	0
Asia	India	Triveni Formulations Limited	Doxycycline	Doxycycline Capsules B.P	WF607	1	1	0	0	1	0	0	1	0	0
Asia	India	Ultra Care International	Sulfa/Trimet	Cotrimoxazole Tablets B.P	UT035	2	2	0	0	2	0	0	2	0	0
Asia	India	UMEDICA Laboratories	Glibenclamide	Glibenclamide	NB502	1	1	0	0	1	0	0	1	0	0
Asia	India	Zee Laboratories	Amoxicillin	Monamox-250 DT	416-170	1	1	0	0	1	0	0	1	0	0
Asia	India	ZIM Laboratories Ltd.	Atenolol	Atenolol Tablets BP	FO38J601	1	1	0	0	1	0	0	1	0	0
Asia	Sultanat of Oman	National Pharmaceutical Industries Co. (SAOG)	Ciprofloxacin	Omacip 500	2016312	1	1	0	0	1	0	0	1	0	0
Asia	Turkey	Bilim Pharmaceuticals	Amoxi/Clav	Klacin BID	16256320A	1	1	0	0	1	0	0	1	0	0
Europe	Austria	Sandoz	Amoxicillin	Amoxycillin Sandoz	GM3744	1	1	0	0	1	0	0	1	0	0
Europe	Austria	Sandoz	Amoxicillin	Amoxycillin Sandoz	HD4437	1	1	0	0	1	0	0	1	0	0
Europe	Austria	Sandoz	Amoxicillin	Amoxycillin Sandoz	HD4445	1	1	0	0	1	0	0	1	0	0
Europe	Austria	Sandoz	Amoxi/Clav	Curam 625	FL5158	1	1	0	0	1	0	0	1	0	0
Europe	Austria	Sandoz	Penicillin V	Ospen	GM5718	1	1	0	0	1	0	0	1	0	0

							USP assay			USP dissolution			USP assay and dissolution combined		
Stated continent of origin	Stated country of origin	Stated manufacturer	INN	Stated product name	Batchnumber	N	complies	deviation	extreme deviation	complies 2	deviation2	extreme deviation 2	complies 3	deviation 3	extreme deviation 3
Europe	Austria	Sandoz	Penicillin V	Ospen	GY5549	2	2	0	0	2	0	0	2	0	0
Europe	Austria	Sandoz	Penicillin V	Ospen	HC8534	1	1	0	0	1	0	0	1	0	0
Europe	Austria	Sandoz	Penicillin V	Ospen	HK8732	1	1	0	0	1	0	0	1	0	0
Europe	Austria	Sandoz	Penicillin V	Starpen	GH3937	1	1	0	0	1	0	0	1	0	0
Europe	Belgium	Merck	Metformin	Glucophage	18664	1	1	0	0	1	0	0	1	0	0
Europe	Belgium	Merck	Metformin	Glucophage	18670	1	1	0	0	1	0	0	1	0	0
Europe	Belgium	Merck	Metformin	Glucophage	F0471	1	1	0	0	1	0	0	1	0	0
Europe	Belgium	Oxford Pharma	Penicillin V	Penicillin-V Tablets	190	1	0	0	1[§]	0	0	1[§]	0	0	1[§]
Europe	Cyprus	Medochemie Ltd.	Amoxi/Clav	Moxiclav 1g	PE042	1	1	0	0	1	0	0	1	0	0
Europe	Cyprus	Medochemie Ltd.	Amoxi/Clav	Moxiclav 625mg	P9H020	1	1	0	0	1	0	0	1	0	0
Europe	Cyprus	Remedica Ltd	Metformin	Glyformin 500	67721	1	1	0	0	1	0	0	1	0	0
Europe	Cyprus	Remedica Ltd	Metformin	Glyformin 500	68397	1	1	0	0	1	0	0	1	0	0
Europe	France	Famar Lyon	Metformin	Glucophage 500 mg	F0554	1	1	0	0	1	0	0	1	0	0
Europe	France	Glaxo Welcome Production	Amoxi/Clav	Augmentin Adultes	2478	1	1	0	0	1	0	0	1	0	0
Europe	France	Glaxo Welcome Production	Amoxi/Clav	Augmentin Adultes	HN8F	2	2	0	0	2	0	0	2	0	0
Europe	France	Laboratoire Bailly-Creat	Sulfa/Trimet	Cotrim Fort	CR479	1	1	0	0	1	0	0	1	0	0
Europe	France	Laboratoire Bailly-Creat	Metronidazole	Creazol	124	1	1	0	0	1	0	0	1	0	0
Europe	France	Laboratoire Bailly-Creat	Doxycycline	Doxycreat	45	1	1	0	0	1	0	0	1	0	0
Europe	France	Laboratoire Bailly-Creat	Doxycycline	Doxycreat	47	1	1	0	0	1	0	0	1	0	0
Europe	France	Laboratoire Bailly-Creat	Doxycycline	Doxycreat	50	1	1	0	0	1	0	0	1	0	0
Europe	France	Laboratoire Bailly-Creat	Doxycycline	Doxycreat	51	1	1	0	0	1	0	0	1	0	0
Europe	France	Laboratoires Bailleul	Doxycycline	Tolexine Ge	T1701500	1	1	0	0	1	0	0	1	0	0
Europe	France	Sanofi-Winthrop Industrie	Glibenclamide	Daonil	6LP5A	1	1	0	0	1	0	0	1	0	0
Europe	France	Sanofi-Winthrop Industrie	Glibenclamide	Daonil	7M74A	1	1	0	0	1	0	0	1	0	0
Europe	France	Sanofi-Winthrop Industrie	Glibenclamide	Daonil	7M74E	1	1	0	0	1	0	0	1	0	0
Europe	France	Sanofi-Winthrop Industrie	Furosemide	Lasilix 40 mg	6NV5A	1	1	0	0	1	0	0	1	0	0
Europe	France	Sanofi-Winthrop Industrie	Furosemide	Lasilix 40 mg	7KF7A	1	1	0	0	1	0	0	1	0	0
Europe	France	Sanofi-Winthrop Industrie	Furosemide	Lasilix 40 mg	7M33F	1	1	0	0	1	0	0	1	0	0
Europe	Germany	Aspen Bad Oldesloe GmbH	Salbutamol	Ventoline	G3415	1	1	0	0	1	0	0	1	0	0
Europe	Germany	Berlin Chemie	Sulfa/Trimet	Berlocid	61001	1	1	0	0	1	0	0	1	0	0
Europe	Germany	Denk Pharma GmbH & Co. KG	Amoxi/Clav	AmoxiClav-Denk	19694	1	1	0	0	1	0	0	1	0	0
Europe	Germany	Denk Pharma GmbH & Co. KG	Amoxi/Clav	AmoxiClav-Denk	20014	1	1	0	0	1	0	0	1	0	0
Europe	Germany	Denk Pharma GmbH & Co. KG	Amoxi/Clav	AmoxiClav-Denk	20517	1	0	1	0	1	0	0	0	1	0
Europe	Germany	Denk Pharma GmbH & Co. KG	Amoxi/Clav	AmoxiClav-Denk	20518	2	1	1	0	2	0	0	1	1	0
Europe	Germany	Denk Pharma GmbH & Co. KG	Atenolol	Atenolol Denk	3231	1	1	0	0	1	0	0	1	0	0
Europe	Germany	Denk Pharma GmbH & Co. KG	Metformin	Metformin Denk	19965	1	1	0	0	1	0	0	1	0	0
Europe	Germany	Denk Pharma GmbH & Co. KG	Metformin	Metformin Denk	20384	1	1	0	0	1	0	0	1	0	0
Europe	Germany	Denk Pharma GmbH & Co. KG	Metformin	Metformin Denk	95H	1	1	0	0	1	0	0	1	0	0

							USP assay			USP dissolution			USP assay and dissolution combined		
Stated continent of origin	Stated country of origin	Stated manufacturer	INN	Stated product name	Batchnumber	N	complies	deviation	extreme deviation	complies 2	deviation2	extreme deviation 2	complies 3	deviation 3	extreme deviation 3
Europe	Germany	Denk Pharma GmbH & Co. KG	Metformin	Metformin Denk	9C7	2	2	0	0	2	0	0	2	0	0
Europe	Germany	Denk Pharma GmbH & Co. KG	Metformin	Metformin Denk	9DE	1	1	0	0	1	0	0	1	0	0
Europe	Germany	Salutas Pharma GmbH	Hydrochlorothiazide	Novartis Access Hydrochlorothiazide	GN5244	2	2	0	0	2	0	0	2	0	0
Europe	Italy	Errekappa Euroterapici S.p.A	Atenolol	Atenolol	0008639	1	1	0	0	1	0	0	1	0	0
Europe	Italy	Laboratori Guidotti S.p.A	Metformin	Metforal	58042	1	1	0	0	1	0	0	1	0	0
Europe	Spain	Ferrer Internacional S.A.	Glibenclamide	Glidiabet	J010	1	1	0	0	1	0	0	1	0	0
Europe	Spain	Ferrer Internacional S.A.	Glibenclamide	Glidiabet	J011	1	1	0	0	1	0	0	1	0	0
Europe	Spain	Ferrer Internacional S.A.	Glibenclamide	Glidiabet	J012	1	1	0	0	1	0	0	1	0	0
Europe	Spain	Novartis Farmacéutica S.A.	Hydrochlorothiazide	Esidrex	B1789	1	1	0	0	1	0	0	1	0	0
Europe	Spain	Novartis Farmacéutica S.A.	Hydrochlorothiazide	Esidrex	BA453	1	1	0	0	1	0	0	1	0	0
Europe	Spain	Novartis Farmacéutica S.A.	Hydrochlorothiazide	Esidrex	BJ475	2	2	0	0	2	0	0	2	0	0
Europe	Spain	Novartis Farmacéutica S.A.	Hydrochlorothiazide	Esidrex	BL800	1	1	0	0	1	0	0	1	0	0
Europe	Spain	Novartis Farmacéutica S.A.	Hydrochlorothiazide	Esidrex	BL801	2	2	0	0	2	0	0	2	0	0
Europe	Spain	Novartis Farmacéutica S.A.	Hydrochlorothiazide	Esidrex	BR630	1	1	0	0	1	0	0	1	0	0
Europe	Spain	Novartis Farmacéutica S.A.	Hydrochlorothiazide	Esidrex	BR631	1	1	0	0	1	0	0	1	0	0
Europe	Spain	Novartis Farmacéutica S.A.	Hydrochlorothiazide	Esidrex	BT359	1	1	0	0	1	0	0	1	0	0
Europe	Spain	Novartis Farmacéutica S.A.	Hydrochlorothiazide	Esidrex	BT900	1	1	0	0	1	0	0	1	0	0
Europe	Spain	Novartis Farmacéutica S.A.	Hydrochlorothiazide	Esidrex	BV229	1	1	0	0	1	0	0	1	0	0
Europe	Spain	Novartis Farmacéutica S.A.	Hydrochlorothiazide	Esidrex	BV384	1	1	0	0	1	0	0	1	0	0
Europe	Spain	Novartis Farmacéutica S.A.	Hydrochlorothiazide	Esidrex	BV831	1	1	0	0	1	0	0	1	0	0
Europe	Sweden	Bluefish Pharmaceuticals AD	Metformin	Metformina Bluefish	5150657	1	1	0	0	1	0	0	1	0	0
Europe	United Kingdom	SmithKline Beecham Pharmaceuticals	Amoxi/Clav	Augmentin	562626	1	0	0	1 [§]	0	0	1 [§]	0	0	1 [§]
Europe	United Kingdom	Sonmart Pharma (UK)	Doxycycline	Doxycycline Capsules	170821	2	2	0	0	2	0	0	2	0	0
Europe	United Kingdom	Sonmart Pharma (UK)	Metformin	Metformin Tablets	170820	2	2	0	0	2	0	0	2	0	0
Europe	United Kingdom	Sonmart Pharma (UK)	Metronidazole	Metronidazole Tablets 250mg	170832	1	1	0	0	1	0	0	1	0	0
Europe	United Kingdom	Sonmart Pharma (UK)	Amoxicillin	Sonmamox Amoxicilline 500mg	170801	1	1	0	0	1	0	0	1	0	0

							USP assay			USP dissolution			USP assay and dissolution combined		
**Stated continent of origin**	**Stated country of origin**	**Stated manufacturer**	**INN**	**Stated product name**	**Batchnumber**	**N**	**complies**	**deviation**	**extreme deviation**	**complies 2**	**deviation2**	**extreme deviation 2**	**complies 3**	**deviation 3**	**extreme deviation 3**
not stated	not stated	Cinpharm **	Amoxi/Clav	Cinclamox	DW3311	2	2	0	0	2	0	0	2	0	0
not stated	not stated	Cinpharm **	Amoxi/Clav	Cinclamox	DW3312	1	1	0	0	1	0	0	1	0	0
not stated	not stated	not stated	Amoxicillin	Filmox 500	SAECB002	1	1	0	0	1	0	0	1	0	0
not stated	not stated	not stated	Salbutamol	not stated	not stated	1	0	1	0	1	0	0	0	1	0
